# Fluid and ion transfer across the blood–brain and blood–cerebrospinal fluid barriers; a comparative account of mechanisms and roles

**DOI:** 10.1186/s12987-016-0040-3

**Published:** 2016-10-31

**Authors:** Stephen B. Hladky, Margery A. Barrand

**Affiliations:** Department of Pharmacology, University of Cambridge, Tennis Court Road, Cambridge, CB2 1PD UK

**Keywords:** Blood–brain barrier, Choroid plexus, Brain interstitial fluid, Cerebrospinal fluid, Fluid secretion, pH regulation, Potassium regulation, Sodium transport, Potassium transport, Chloride transport, Bicarbonate transport, Tight junctions, Water channels, Paracellular transport, Transcellular transport, Ion transporters, Ion channels, Electroneutrality, Endothelial, Epithelial, Neurovascular unit, Astrocyte endfeet

## Abstract

The two major interfaces separating brain and blood have different primary roles. The choroid plexuses secrete cerebrospinal fluid into the ventricles, accounting for most net fluid entry to the brain. Aquaporin, AQP1, allows water transfer across the apical surface of the choroid epithelium; another protein, perhaps GLUT1, is important on the basolateral surface. Fluid secretion is driven by apical Na^+^-pumps. K^+^ secretion occurs via net paracellular influx through relatively leaky tight junctions partially offset by transcellular efflux. The blood–brain barrier lining brain microvasculature, allows passage of O_2_, CO_2_, and glucose as required for brain cell metabolism. Because of high resistance tight junctions between microvascular endothelial cells transport of most polar solutes is greatly restricted. Because solute permeability is low, hydrostatic pressure differences cannot account for net fluid movement; however, water permeability is sufficient for fluid secretion with water following net solute transport. The endothelial cells have ion transporters that, if appropriately arranged, could support fluid secretion. Evidence favours a rate smaller than, but not much smaller than, that of the choroid plexuses. At the blood–brain barrier Na^+^ tracer influx into the brain substantially exceeds any possible net flux. The tracer flux may occur primarily by a paracellular route. The blood–brain barrier is the most important interface for maintaining interstitial fluid (ISF) K^+^ concentration within tight limits. This is most likely because Na^+^-pumps vary the rate at which K^+^ is transported out of ISF in response to small changes in K^+^ concentration. There is also evidence for functional regulation of K^+^ transporters with chronic changes in plasma concentration. The blood–brain barrier is also important in regulating HCO_3_^−^ and pH in ISF: the principles of this regulation are reviewed. Whether the rate of blood–brain barrier HCO_3_^−^ transport is slow or fast is discussed critically: a slow transport rate comparable to those of other ions is favoured. In metabolic acidosis and alkalosis variations in HCO_3_^−^ concentration and pH are much smaller in ISF than in plasma whereas in respiratory acidosis variations in pH_ISF_ and pH_plasma_ are similar. The key similarities and differences of the two interfaces are summarized.

## Background

**Fig. 1:**
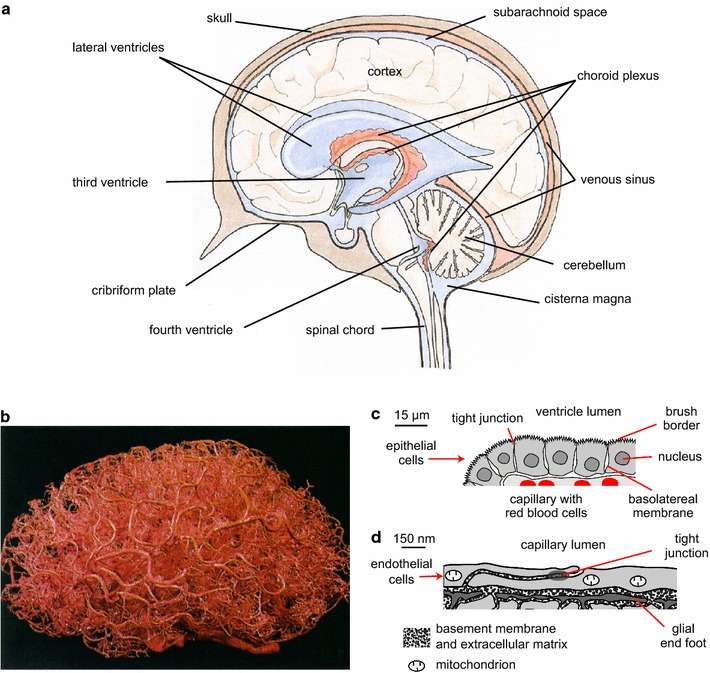
Locations and functions of the choroid plexuses and the blood brain barrier. **a** The choroid plexuses are discrete structures located in the cerebral ventricles, which are filled with cerebrospinal fluid. Fluid can flow from the ventricles into the cisterna magna and from there to the subarachnoid spaces of the brain and spinal cord. **b** A cast of the vascular system of a human brain. The blood–brain barrier, which comprises the lining of the smallest and most numerous branches of the vascular system, the microvessels, is present almost everywhere in the brain. **c** Diagram of a cross section of part of a single villus of a choroid plexus as would be seen by light microscopy. The apical brush border is well separated from most of the basolateral membrane. **d** Diagram of a cross section of a microvessel wall and adjacent parenchyma as could be seen in an electron micrograph. Note the difference in scales in **c** and **d**. **a**–**d** are reproduced with permission: **a** from [26] as relabelled in [15], **b** from [536] (promotional and commercial use of the material in print, digital or mobile device format is prohibited without the permission from the publisher Wolters Kluwer. Please contact healthpermissions@wolterskluwer.com for further information), **c**, **d** from [15]. For an image of part of a choroid plexus see Fig. 5

The inorganic ions in brain fluids ultimately derive from the peripheral circulation. They are delivered across either of two major blood–brain interfaces: the choroid plexuses situated within the ventricles and the blood–brain barrier lining the blood vessels dispersed throughout the brain parenchyma (see Fig. 1). The relative contributions of these two interfaces to production of brain fluids and to regulation of their ionic compositions are the key issues discussed in this review.

### Overview of locations and functions of the choroid plexuses and the blood–brain barrier

The choroid plexuses constitute the interface between blood and cerebrospinal fluid (CSF) in the ventricles. There are four such plexuses protruding into the ventricles, one in each of the lateral ventricles, one in the IIIrd and one in the IVth ventricle (see Fig. 1a). As seen in the light microscope each choroid plexus has a frond-like shape with many villi, each with a layer of cuboidal epithelial cells overlying blood microvessels of the fenestrated type (see Fig. 1c). Even on this scale the epithelial layer appears to have a large surface area. Furthermore the apical brush border and basolateral in foldings make the actual membrane area of the epithelial cells much greater still (see [1, 2]). As described in detail by Cserr [3] and more recently by Damkier et al. [4] the epithelial layer has all the hallmarks of a “leaky” secretory epithelium designed to produce a large volume of nearly isosmotic fluid.

The blood–brain barrier is in some ways a more complicated structure than the choroid plexus. It separates blood from interstitial fluid (ISF) and cells of the brain parenchyma. The barrier, so-called because it greatly restricts movements of many substances between brain and blood, consists of the endothelial lining of almost the entirety of the brain microvascular network (see Fig. 1b). However, the transfers the barrier permits are at least as important as those it hinders. It is ideally placed both to deliver substrates for brain cell metabolism and to remove the corresponding wastes. It is also important in regulating ISF ionic composition.

The lining of the brain microvessels differs from that of peripheral vessels in that the endothelial cells are joined together by tight junctions that greatly restrict free, paracellular movement of substances (see Fig. 1d), the exceptions to this being the parts of the vasculature supplying the choroid plexuses and the circumventricular organs. The permeability of the blood–brain barrier to ions such as Na^+^ and Cl^−^ is low, not much larger than the permeability of many cell membranes (see Sect. 4). While the low passive permeability to these ions as judged from unidirectional tracer fluxes may reflect primarily paracellular movements, the net fluxes can still reflect transcellular transport through the cells (see Sect. 4.3.4). The endothelial layer is surrounded by a basement membrane and pericytes all closely enveloped by astrocyte (glial) endfeet (see Fig. 1d) [5–7]. The pericytes have a contractile function (see Sect. 2.3) as well as a role in inducing and maintaining barrier properties [8–10]. There are also nerve cells close by within the parenchyma. This whole assembly is called the neurovascular unit. All the various components of the neurovascular unit may influence fluid movement into and out of the brain but the major elements to be considered are endothelial cells and astrocytes.

The astrocyte endfeet are connected together by gap junctions but the clefts between them are not sealed by tight junctions and thus are routes for passage of water and solutes including markers as large as horseradish peroxidase [11, 12] between the basement membrane and the interstitial spaces. However, there is evidence that movement through the clefts can be slow compared to that along the basement membrane and that, at least under some circumstances the endfoot layer can present a major barrier to transport between the blood and the brain parenchyma [13, 14]. The extent to which the astrocytes and pericytes cover the endothelial tube has been calculated by Mathiisen et al. [5] from serial sections of the CA1 layer of rat hippocampus. They found that the clefts available for diffusion away from the tube to the interstitium occupy only 0.3% of the surface area.

Consideration of the structures and locations of the choroid plexuses and the blood–brain barrier suggests that they fulfil different roles in fluid regulation. The choroid plexuses are well defined structures located within the ventricles surrounded by the fluid they secrete. This positions them to provide the brain as a whole with a fluid of controlled composition that gives buoyancy and provides a route for removal of wastes by bulk flow of fluid through the routes of outflow. Bulk movement of CSF between brain and spinal cord also allows compensation for changes in blood volume within the skull during the cardiac and respiratory cycles (see discussion in [15]). The blood–brain barrier is a much more diffuse structure with parts of it close to every cell in the brain (see Fig. 1b). This is essential to its primary role in supplying O_2_, CO_2_ and glucose and removing waste products as the distance that these substances have to diffuse between blood and brain cells must be kept small (see Sect. 2). Whether it has a secondary role in providing fluid to the brain remains controversial (see Sect. 4.1).

This review is mainly concerned with transport of Na^+^, K^+^, Cl^−^, HCO_3_^−^ and water across the barriers. However consideration is also given in Sect. 2 to transfer of glucose, CO_2_, O_2_ and amino acids. The mechanisms for ion and water transport are discussed in Sects. 3 for the choroid plexuses and 4 for the blood–brain barrier. Sections 5 and 6 consider the roles of transport across both interfaces in the regulation of [K^+^] and [HCO_3_^−^] in ISF and CSF. Sect. 7 summarizes the main points of comparison between the two interfaces. Finally Sect. 8 indicates the major conclusions concerning the roles of the choroid plexuses and the blood–brain barrier and highlights areas of inadequate knowledge for future investigation.

### Previous reviews

The present review is the second part of a survey of work on the extracellular fluids of the brain. The first part [15] considered the basic processes, including secretion, filtration, diffusion and bulk flow; the use of markers (e.g. radiotracers or fluorescent molecules) to follow fluid movements; the pathways available for transfers within the brain; and recent work on the patterns of flow.

There have been a number of reviews of the topics considered in this the second part. Cserr’s “The Physiology of the Choroid Plexus” [3] and Bradbury’s “The Concept of the Blood–brain barrier” [16] are both still important resources more than 35 years after they were written. Davson and Segal’s book [17] provides encyclopaedic coverage up to the mid-1990s roughly the time when the focus of research shifted from function in vivo towards molecular and cellular mechanisms. Recent, major reviews are available for studies on the choroid plexus [2, 4], the blood–brain barrier [18, 19], and the functions of CSF and fluid movements within the brain [20, 21]. Major reviews on the transport of HCO_3_^−^ and regulation of brain extracellular fluid pH are cited in Sect. 6.

There have also been a number of reviews of related material not covered in this review including the development of the blood–brain barrier, its structural basis, the extent to which it restricts penetration by a large variety of substances, and the efforts that have been made to circumvent the barrier function. Interested readers are directed to [22–30].

### Notation and conventions for expressing concentrations, partial pressures and other values

Throughout this review, enclosing the symbol for a substance in square brackets, e.g. [HCO_3_^−^], is used to stand for the concentration of that substance as a molality defined as the number of moles per kilogram of solvent, i.e. with units mol kg^−1^. However when concentrations have been reported as molarities, mole per litre of solution with units mol l^−1^ the same symbol is used. Molality is preferable when referring to intracellular concentrations or concentrations in plasma but when referring to the extracellular fluids of the brain, which are almost protein free, either is suitable. Subscripts are used to indicate location, e.g. [HCO_3_^−^]_CSF_ is the concentration of HCO_3_^−^ in cerebrospinal fluid. A lower case “p” preceding the symbol for a substance means partial pressure, e.g. pCO_2_, is the partial pressure of CO_2_. Finally, unless otherwise stated values are scaled to those that would be found in a human with a 1400 g brain.

## Transfers of water, O_2_, CO_2_ and major nutrients between blood and brain parenchyma

The largest transfers of substances into or out of the brain parenchyma are those of water, glucose, O_2_, CO_2_ and to a lesser extent amino acids taken collectively. Most of the fluxes of these substances must be across the blood–brain barrier because the blood flow to the choroid plexuses is insufficient to supply or remove the amounts needed. Although blood flow per unit mass of tissue is much greater in the choroid plexuses than in cerebral cortex, ~9.8-fold in dogs and rabbits [31] and 2.8- to 5.5-fold in different studies in rats [32–34], the mass of the combined choroid plexuses is only a small fraction of that of the brain as a whole, 0.0012, 0.0029 and 0.0021 in dog, rabbit and rat [35]. Thus the proportion of cerebral blood flow that goes to the choroid plexuses is less than ~1% while the percentages of water, glucose and O_2_ entering the brain are much larger as described in Sects. 2.1, 2.2 and 2.3.

### Water movement at the blood–brain barrier and choroid plexuses

**Fig. 2:**
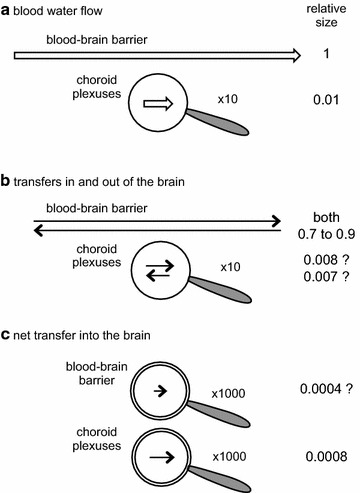
Comparison of **a** blood water flow and **b**, **c** transfers of water across the blood–brain barrier and the choroid plexuses. For the choroid plexus flow and transfers in and out of the brain are shown through a ×10 magnifying glass. The very much smaller net flows of water across both barriers are shown magnified ×1000. *Arrow* lengths are an approximate guide to sizes of the transfers. The water flow along the blood vessels, ~85% of blood flow, is ~100-fold greater than that to the choroid plexuses. Because the transfers of water across the interfaces are blood flow limited, the transfers in and out of the brain are also ~100-fold greater at the blood–brain barrier. By contrast because net transfers reflect active secretion of fluid, the very much smaller net transfer of water is almost certainly greater across the plexuses than across the blood–brain barrier

Measurements with tritiated water have shown that 70–90% of the water molecules in the blood perfusing the brain cross the blood–brain barrier and enter the brain tissues in a single pass ([chapter 4 in 16], [36–42]). If cerebral blood flow is 800 ml min^−1^ [43] then brain blood water flow, the water flow along the capillaries, is perhaps 0.85 × 800 ml min^−1^ = 680 ml min^−1^ (the rest is made up of solutes such as haemoglobin). Of that ~0.7 × 680 ml min^−1^ = ~476 ml min^−1^ = ~685 l day^−1^ enters the brain, mostly across the blood–brain barrier. This is 37,700 mol/day! However, (see Fig. 2) everywhere in the brain the influx is balanced by an almost equal efflux primarily because the very large concentration of water, ~55 M, is almost the same on the two sides of the interfaces. A figure 1000-fold smaller than the unidirectional movement, i.e. 685 ml day^−1^ rather than 685 l day^−1^, would be a high estimate of the total net movement of water into the brain per day across the blood–brain interfaces (see Sects. 3.2 and 4.1 and section 2.6 in [15]). The mechanisms for water movement are considered in Sects. 3.4.2 and 4.3.6.

There have been repeated attempts to base descriptions of CSF production and reabsorption on measurements of tracer fluxes of water [44–47] but these have been ill conceived. As explained in detail elsewhere (section 2.6 of [15]) and illustrated in Fig. 2, unidirectional tracer fluxes of water far exceed net fluxes. These measurements have never been sufficiently accurate that they could be used to determine either the magnitude or the site of net flux of water (or flow) into or out of any tissues including those of the CNS.

### O_2_ and CO_2_ transfer at the blood–brain barrier and production of metabolic water

About 90–95% [48] of the metabolism of ~0.6 mol of glucose day^−1^ in the brain [49, 50] is complete oxidation consuming ~3.3 mol day^−1^ of O_2_ and producing the same daily amounts of CO_2_ and water. The diffusion distances to and from the capillaries are small and O_2_ and CO_2_ easily diffuse across the membranes of the blood–brain barrier endothelial cells (see item (3) in Sect. 6.4.2, [51] and for early references [52]). Thus they can be transferred to and from the blood driven by their concentration gradients.

### Importance of neurovascular coupling for O_2_ and CO_2_ transfer at the blood–brain barrier

Increased neuronal activity in the brain is associated with increased blood flow, an example of functional hyperaemia common to all tissues. In the brain this is called neurovascular coupling. Blood flow in the brain can be increased by dilation of small arterioles, which are the principal site of resistance, but also more locally by dilation of capillaries brought about by changes in pericyte activity [53–55]. How the activities of these effectors are coupled to neural activity and the nature of the signals involved have been the subjects of much discussion.

During neuronal activity more O_2_ enters the brain parenchyma to supply part of that needed for increased metabolism (see [56, 57] for references). The amount of O_2_ stored within the brain is limited and even the resting rate of metabolism can deplete it within seconds. However, delivery of O_2_ in the blood is strongly “buffered” by haemoglobin in the red blood cells, and it may be that even normal blood flow is adequate to support the O_2_ requirements of activity [58]. There is little evidence that falls in pO_2_ either in arterial blood or within the brain are directly involved in stimulating the increased cerebral blood flow until these falls are substantial [59–63]. Similarly relatively large changes in [glucose]_plasma_ appear to have no effect on neurovascular coupling [64]. By contrast it is clear that even small increases in pCO_2_ of arterial blood or decreases in pH in CSF can produce marked vasodilation and decreases in pCO_2_ or increases in pH can produce vasoconstriction ([59, 61, 65–68] and clinical consequences [69]). This cerebrovascular reactivity is closely related to the oldest hypothesis of the mechanism of neurovascular coupling: that it results from the effects of acidic products of metabolism, e.g. CO_2_ and lactic acid, and the associated fall in pH [70].

Suggestions that the control of blood flow is actually not just in response to changes in pO_2_, pCO_2_ and pH began with the observation that the increase in blood flow reflects, at least in part, arteriolar dilation at some distance upstream along the blood vessels from the site of O_2_ consumption and CO_2_ release [71]. (The involvement of astrocytes in neurovascular coupling may provide the mechanism for the signals to spread from the immediate site of the neural activity to the arterioles.) It was also observed that local pO_2_ hardly changes or can actually increase, presumably as a consequence of the increased flow, rather than decrease as would be required for it to be the cause [53, 71–74]. Furthermore the change in pH during neural activity while clearly present [75] was too slow to account for the initiation of the increased blood flow [53, 71, 73, 76] and initially it could even be in the wrong direction [77]. However, it should not be forgotten that based on the evidence from the effects of changes in arterial pCO_2_ and CSF pH, the fall in pH that occurs with sustained neuronal activity is likely to have an effect to increase blood flow.

**Fig. 3:**
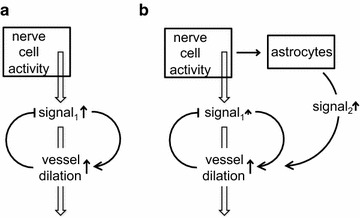
Diagram illustrating possible schemes for neurovascular coupling, i.e. regulation of blood flow changes associated with nerve activity. Two forms of control are shown, **a** simple feedback based on the signal to be regulated, e.g. pCO_2_, and **b** feedback plus feed-forward. The feed-forward element, signal_2_, possibly from astrocytes in **b**, allows blood flow to increase with smaller changes in the primary quantity to be regulated, signal_1_

There is a strong teleological argument in favour of a more complicated mechanism of control than simple feedback. With simple feedback based on monitoring pCO_2_ (or pH of the ISF, see e.g. [59]), in order to stimulate increased blood flow, the pCO_2_ would have to be increased throughout the period of increased neural activity (see Fig. 3a). Better regulation can be produced if nervous activity releases other mediators. This can be either directly from neurons or, as now thought to be more important, from astrocytes. These mediators affecting the smooth muscle of arterioles and pericytes provide a feed forward system in which the increased activity is signalled to the blood vessels independently of changes in pCO_2_ and pH (see Fig. 3b).

There are many possible regulatory signals (S_2_ in Fig. 3b) that can couple nerve activity to vasodilation and there is now an extensive literature, comprehensively reviewed elsewhere, on the roles of astrocytes, elevation of Ca^2+^ in their endfeet and the release of arachidonic acid metabolites, NO and K^+^ in neurovascular coupling [53–55, 78–88].

While it is clear that changes in blood flow are important in the supply of O_2_ and removal of CO_2_, they are thought to be less important for supplying glucose (see Sect. 2.4.1). However, recent attention has been given to the consequences of redistributing blood flow and it has been found that this can change the proportions of both O_2_ and glucose extracted from the blood [89, 90]. Variations in blood flow are unlikely to have significant effects on fluid secretion at the blood–brain barrier. This is because the net rates of ion and water transfers are relatively small compared to the rates of delivery in the blood, i.e. blood flow is not limiting.

### Glucose and amino acid transfer at the blood–brain barrier

#### Glucose

Glucose consumption by the brain [49, 50] amounts to ~0.6 mol day^−1^ almost all of which must cross the blood–brain barrier. The blood flow to the brain, ~800 ml min^−1^ [43] delivers ~400 ml min^−1^ of plasma containing ~5 mmol l^−1^ glucose, which equates to ~2 mmol min^−1^ = 2.9 mol day^−1^, which corresponds to extraction of ~0.6/2.9 × 100% = ~20% of the arriving glucose. Experimentally measured extractions for physiological [glucose]_plasma_ vary between 15 and 35% [39, 91–93].

Glucose transport is stereo-selective with that of d-glucose (usually called just “glucose”) being very rapid while that of l-glucose is slow, comparable to that for other polar solutes like sucrose and mannitol [39, 91–94]. Glucose transport across the blood–brain barrier is passive but mediated by specific, saturable GLUT1 transporters expressed in both the luminal and abluminal membranes of the endothelial cells [95–98]. Substantial amounts of the transporters are also found within the endothelial cell cytoplasm [99], presumably on vesicular membranes acting as a reservoir (see below).

The influx of glucose from blood to brain exceeds the efflux from brain to blood leaving a relatively large inward net flux, about 30% of the measured influx (see e.g. [100, 101]). At a simple level this would be expected from a lower [glucose] in ISF than in blood plasma. However, if the fluxes were by simple diffusion across a single barrier, the measured concentrations, ca. 1 mM [102] or even less [103] in ISF and at least 4 mM in blood plasma, would predict efflux less than one-fourth of influx rather than the roughly two-thirds observed. At least part of the explanation is that the fluxes occur by a saturable mechanism (as reviewed in [102]) such that the unidirectional fluxes increase less than linearly with concentration, hence the difference between influx and efflux will not be as large as for simple diffusion across a single barrier. This will have the effect of limiting the impact on the brain of changes in [glucose]_plasma_.

**Fig. 4:**
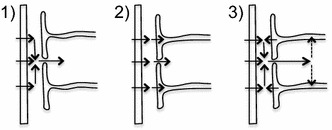
Scenarios for transport from blood into the brain. Substances are transported across the endothelial cells (*left*) into the basement membrane. In scheme (*1*) net onward transport is entirely via the clefts between the astrocyte endfeet (*right*). This may be the pattern for Na^+^ and Cl^−^. In scheme (*2*) net onward transport occurs both via the clefts and across the endfoot membrane into the astrocytes. This may be the pattern for glucose and K^+^. In scheme (*3*) net transport across the endfoot membrane is from the astrocyte into the basement membrane. The combination of substances arriving across the endothelial cells and from the astrocytes then enters the ISF via the clefts. This may be the pattern for water. There are other possible schemes, e.g. with the directions reversed which may occur when K^+^ is being transported from the brain to the blood

An additional factor is that glucose after crossing the endothelial cells may enter astrocyte endfeet rather than diffusing away through the clefts between them. At least some of this glucose can be transported back out of the endfeet and across the endothelial cells contributing to the efflux.^1^ At least three scenarios for transport from blood into the brain can be envisaged (see Fig. 4): (1) water or solute may cross the endothelium, diffuse (or be moved by flow) within the basement membrane parallel to the endothelial surface until it reaches a cleft and then diffuse or flow outwards through the cleft; (2) it could cross the endothelium and basement membrane and then enter an astrocyte endfoot; or (3) it could enter the basement membrane both from the endothelial cells and from the endfeet and leave by way of the clefts. Combinations of these are also possible. Which of the above occurs will depend upon the transporters available and on the concentrations within the basement membrane. Scenarios 1 and 3 will be considered later in conjunction with transport of Na^+^, Cl^−^ and water (see Sect. 4.6.4) and scenario 2 for transport of K^+^ (see Sect. 5). Transport of glucose is likely to be an example of scenario 2.

Part of the concentration gradient for glucose between blood and ISF leads to a net flux across the endothelial cells into the basement membrane and part must lead to transport from the basement membrane into the rest of the brain. Barros et al. [102] calculated for glucose that in order for even the full concentration difference between blood and interstitial fluid to drive diffusion at the observed rate through the clefts between the endfeet, the clefts would need to occupy at least 0.2% of the surface area. Thus in view of the finding that only 0.3% of the area is cleft (see Sect. 1.1), barely more than the minimum they calculated, it is likely that much of the glucose enters the endfeet via GLUT1. This transporter is prominently expressed in the endfeet [102, 104, 105] (see also footnote 1). The extent to which glucose is metabolized to lactic acid within astrocytes rather than being passed on directly to the neurons is beyond the scope of this review. It has been the subject of a large literature; see [57, 97, 102, 106–108] for references.

Glucose supply to the brain is increased during sustained neural activity [102, 109–111]. Barros et al. [102] discuss the evidence that this requires stimulation of both hexokinase within the brain [112] and transport across the endothelial cells (see also footnote 1). Part of the increase in transport may reflect redistribution of GLUT1 from the cytoplasm to plasma membranes in the endothelial cells [99]. Redistribution of blood-flow [90, 113] is also thought to affect the proportion of glucose extracted from the blood [94, 114].

Although this review is primarily concerned with fluid movement and transport of inorganic ions, glucose has been discussed here because so much is transported and the principal glucose carrier at the blood–brain barrier, GLUT1, may allow movement of water.

#### Amino acids

Likewise transfer of amino acids needs to be mentioned because amino acid efflux from the brain into the endothelial cells is primarily Na^+^-coupled and the Na^+^ flux involved is larger than, and in the opposite direction to, the net flux needed to support fluid secretion. As a historical note, the first demonstration of functional polarity of the blood–brain barrier was for amino acid transport [115].

The blood–brain barrier greatly restricts influx of some amino acids including the neurotransmitters glutamate and glycine, but allows rapid, passive but saturable influx of many others including all those classified as essential [92, 116, 117]. For instance from the data in [92], it is evident that more than 30% of phenylalanine arriving in the blood enters the brain. Smith and Stoll [118] list the influx rates in perfused dog brains observed from mixtures of amino acids at concentrations only a bit less than normal. The total rate of influx for those listed, 72 nmol min^−1^ g^−1^ (which scales to 145 mmol day^−1^ for a 1400 g human brain), is balanced by a nearly equal total efflux [119].^2^ This efflux is driven uphill across the abluminal membranes of the endothelial cells from the brain by the coupled movement of Na^+^ [120]. As mentioned above, this Na^+^ movement is in the opposite direction to that for net secretion of Na^+^ into the brain.

Most, perhaps almost all, of the Na^+^-linked amino acid transporters are on the abluminal side extracting amino acids from the brain [120] (but see [121, 122] and 3). The net Na^+^ flux into the endothelial cells by these Na^+^-linked transporters must be balanced by a net Na^+^ flux out of the cells via the Na^+^-pump. These fluxes cannot be ignored when considering net Na^+^ flux across the barrier, see Sect. 4.6.1.

### Transfers of glucose and amino acids across the choroid plexuses

Transport of glucose [123, 124] and amino acids [125, 126] also takes place across the choroid plexuses. For glucose the net flux can be estimated as roughly 1.4 mmol day^−1^ from the rate of formation of CSF in the ventricles, ~400 ml day^−1^ and (glucose) in the secretion, ~3 mM. This is much less than the net flux of glucose across the blood–brain barrier, about 600 mmol day^−1^. The passive glucose transporter, GLUT1 is expressed in the choroid plexuses with greater amount found in the basolateral membrane than in the apical membrane [127–132]. This suggests that it may be primarily involved either with supply of glucose for metabolism within the epithelial cells [133] or with another function such as increasing the water permeability of the basolateral membrane (see Sect. 3.4.2).

The transport of amino acids from blood can be detected from the differences in their concentrations in the arterial and venous blood supplying and draining the choroid plexuses. Transport from CSF into blood can be detected by the appearance in venous blood of tracers added to CSF in the ventricles [125, 126]. Extraction of amino acids from blood can be substantial although just as for glucose the total amounts of amino acids entering the brain are much smaller via the choroid plexuses than via the blood–brain barrier.

## Fluid secretion by the choroid plexuses

The primary function of the choroid plexuses is to produce CSF [2–4] with the rate of fluid secretion being <~20% of the blood plasma flow to the plexuses in rats (see e.g. [134]) and perhaps a lower percentage in humans.^4^ The roles of the choroid plexuses in supplying micronutrients, vitamins, Ca^2+^ and Mg^2+^ and in actively excluding or removing other substances are also important (for reviews see e.g. [2, 16, 21, 135–138]). Similarly, the plexuses play a critical role in development of the brain and the provision of growth factors (see [139] and references therein).

### Composition of fluid secreted by the choroid plexuses

The composition of recently formed CSF can be determined, at least approximately, by direct measurements of the fluid close to the choroid plexuses [140–142]. The composition of this fluid is similar but not identical (see Table 1) to that of CSF in the ventricles, i.e. a slightly hyperosmotic (perhaps by 1–5 mOsmol kg^−1^) solution of Na^+^, K^+^, Cl^−^, HCO_3_^−^ and small amounts of many other solutes like Mg^2+^, HPO_3_^2−^, glucose and amino acids (see Table 2.5 in [17] and [4] for further discussion).

### Rate of fluid secretion across the choroid plexuses

The newly secreted CSF leaves the region of the choroid plexuses and emerges from the ventricles into the cisterna magna. The time averaged outflow from the ventricles can be measured by ventriculo-cisternal perfusion and corresponds at least approximately to the rate of production of fluid by the choroid plexuses, 350–500 ml day^−1^ (see section 4.2 in [15] and [2, 143]). It is notable that this rate also corresponds within the fairly wide experimental error margins of the measurements to the total rate of CSF production as determined by continuous collection of the formed CSF from the lumbar sac (for discussion see [17] and section 3.1.3 in [15]). The contribution of fluid secretion by the blood–brain barrier to the measured rates of CSF production is considered in Sect. 4.1.

### Mechanisms of fluid secretion by the choroid plexuses

The mechanisms of secretion by the choroid plexuses have been reviewed recently and comprehensively by Damkier et al. [4] so evidence will be considered only for specific points that remain controversial or where the account differs from that in Damkier et al. It is now generally accepted that the choroid plexuses are the main source of CSF in the ventricles and that the process producing CSF is primarily secretion (i.e. driven by energy supplied from metabolism of the epithelial cells) rather than filtration (driven by energy obtained from the pressure, concentration and potential gradients imposed from outside the epithelial cells). Readers interested in the history of these issues should consult the discussions by Cserr [3], Davson and Segal [17], Damkier et al. [4] and Spector et al. [2].

Four of the simplest arguments that CSF production occurs largely as a secretion by the choroid plexuses arise from considering (a) the composition of the CSF, (b) the effects of inhibitors, (c) the effects of gene silencing, and (d) data from in vitro models of the epithelial layer. (a) The composition of CSF is not that of an ultrafiltrate. (b) The net production of CSF can be inhibited by drugs that interfere with cellular metabolism or with the coupling of metabolism to ion transport, e.g. mitochondrial uncouplers, the Na^+^-pump inhibitor ouabain [144], and the carbonic anhydrase inhibitor acetazolamide [17]. (c) It has been shown that silencing of genes for specific transporters expressed strongly in the choroid plexuses, but weakly elsewhere, reduces the secretion rate [4, 145]. (d) Choroid plexus epithelial cells when cultured as monolayers in vitro have been shown to secrete fluid robustly [146, 147].

### Maintenance of nearly isosmotic fluid secretion by the choroid plexuses

#### Comparisons with kidney proximal tubules

Parallels can be found between fluid transport by the choroid plexuses and that by the renal proximal tubule: the fluids transported are both nearly isosmotic with plasma and the rates of transport per gram of tissue appear to be comparable [148]. The principal role of each of these epithelia is to transport a substantial quantity of fluid leaving behind the “undesirables” and there are other parallels in their functions [21]. At a mechanistic level the Na^+^-pumps in both are located on the side towards which there is a net fluid flow but there are substantial differences in their handling of small ions including HCO_3_^−^, the transport of glucose and the role of paracellular transport.

It is by no means certain how water transport is linked to that of solutes in either the choroid plexus (see [4]) or the proximal tubule. However, in the latter there is clear experimental evidence from studies in which net fluxes of both NaCl and water were eliminated, that the epithelium can maintain a gradient of NaCl with the water coming as close as can be determined to osmotic equilibrium [149, 150].^5^ This does not prove the absence of active transport of water, but it does demonstrate that the rate of any such active transport (including secondary active transport via solute transporters) is not sufficient compared to osmotically driven water movements to produce a measurable gradient of osmolality. The evidence for the choroid plexus is not so clear cut because all available data come from experiments in which net fluxes of water and solutes were not eliminated and thus there were complications resulting from unstirred layers. Such complications can give the appearance of active transport of water when there is none [151]. This is considered further in footnote 5.

#### Transcellular and paracellular routes for water transfer

Osmotically driven water movement across a choroid plexus requires routes that have sufficiently high water permeability. That AQP1 water channels are important in the secretion process and thus that transcellular routes are important may be inferred from the observation that in AQP1 knock-out mice the rate of choroid plexus secretion is 25% lower than that in normal mice, with an 80% lower water permeability of the apical membrane in which most of the AQP1 is expressed [152]. However, there are far fewer aquaporins present on the basolateral side [153] and for the transcellular route the water must cross this membrane as well. The sidedness of the AQP1 distribution remains puzzling [4]. One possibility is that the water permeability of the basolateral membrane is increased by the presence of a protein or proteins other than AQP1. One candidate is the glucose carrier GLUT1 (see Sect. 2.5). This particular carrier has been shown to produce water permeability in membranes [154–157] and is highly expressed in the basolateral membrane of the choroid plexus [127–129, 132, 158].

It has not been established that the osmotic gradients and water permeability are large enough to account for the water fluxes across the choroid plexus without some form of active transport of water [4]. It is very likely, almost inevitable, that there will be some secondary active transport of water by coupling water movements to those of hydrophilic solutes including ions in their respective transporters [156, 157, 159–161]. However, there remains considerable scepticism that such internal coupling can move as much water as required or achieve the final result of a nearly isosmotic secretion (see e.g. [151, 162, 163]).

As discussed by Damkier et al. [4] water flux across the choroidal epithelium might also occur paracellularly passing through tight junctions and lateral spaces between the cells. The permeability of tight junctions to water or solutes is determined by the profile of proteins that they contain, in particular the specific forms of claudin, a family of transmembrane proteins. The claudins most highly expressed at the choroid plexuses are claudin-1, -2 and -3 together with -9, -19, and -20 [24]. Claudin-2 has been especially well studied [164, 165] and shown to form narrow (0.65–0.75 nm), water-filled cation-selective (P_Na_/P_Cl_ of 6–8) paracellular pores, selectivity being conferred by a negatively charged site within the pore. Claudin-2 is a typical component of leaky epithelia such as proximal tubule and choroid plexus that have high water-transport rates [166]. Experiments by Rosenthal et al. [166] have shown that the presence of claudin-2 in the tight junctions of an otherwise tight epithelium is associated with enhanced water flux and increased paracellular Na^+^ flow. During development, the expression of claudin-2 relative to other claudins increases in the choroid plexuses. As noted by Strazielle and coworkers [24] this parallels the increasing rate of CSF secretion. It is highly relevant that the profile of claudins expressed in the tight junctions between endothelial cells at the blood–brain barrier is different from that found between epithelial cells in the choroid plexuses and reflects the tighter barrier of the former [24].

### Expression of ion transporters

The whole profile of transporters expressed in the choroid plexus has been the subject of major transcriptome studies comparing adult and embryonic tissue [25, 167].

**Fig. 5:**
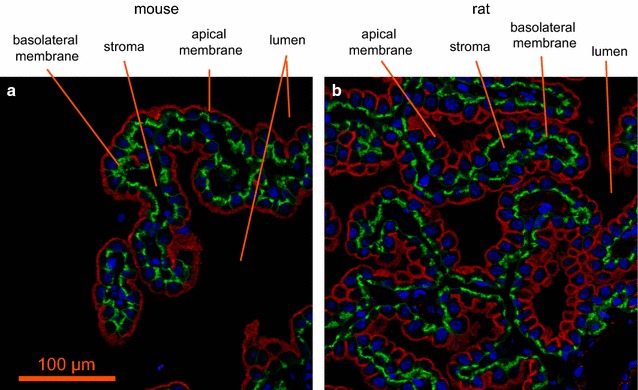
Immunofluorescence staining of ion transporters in the choroid plexus of the IVth ventricle in **a** mouse or **b** rat. The Na^+^, K^+^-ATPase (*red*) is prominent in the apical brush border of the epithelial cells facing the lumen of the ventricle. The Na^+^, HCO_3_^−^ cotransporter, NCBE/NBCn2 (*green*) is localized to the basolateral membranes of the epithelial cells facing the stroma (interstitium) in which are embedded the capillaries. Nuclei are stained with To-pro 3 DNA stain (*blue*). *Scale bar* 100 µm. Previously unpublished images provided by Dr. Jeppe Praetorius. Antibodies: Na^+^, K^+^-ATPase α1-subunit [537]; Slc4a10/Ncbe/NBCn2 [538] and To-pro 3 DNA stain (invitrogen). For a similar fluorescence image localizing NBCe2 to the brush border see [539]. For images that localize Na^+^, K^+^-ATPase to the brush border, AQP1 primarily but not exclusively to the brush border, and Ncbe/NBCn2 and AE2 to the basolateral membrane see [540, 541]

**Fig. 6:**
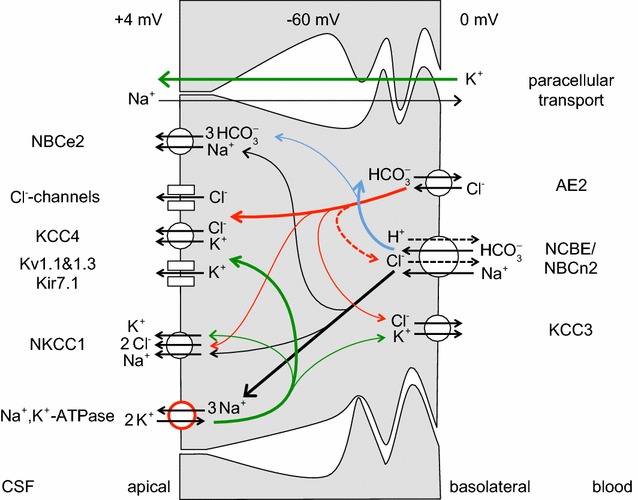
Ion transporters and transport pathways involved in normal secretion by the choroid plexus based on the description in Damkier et al. [4] with some modifications. See also [542]. On the CSF side, Na^+^, K^+^-ATPase actively transports Na^+^ out of and K^+^ into the epithelial cell, maintaining the gradients that drive the other ion movements indicated. The *red circle* used in the symbol for this pump indicates that energy for the transport is input from hydrolysis of ATP. *Arrows* within the cell indicate transfers: in *black* Na^+^, in *green* K^+^, in *red* Cl^−^, and in *blue* HCO_3_^−^. On the blood side, *dashed arrows* in the symbol for the Na^+^, HCO_3_^−^-cotransporter, NCBE/NBCn2, indicate the involvement of H^+^ and Cl^−^ if the transporter is NCBE, but not if the transporter is NBCn2. On the CSF side, transport via the Na^+^, K^+^, 2 Cl^−^-cotransporter, NKCC1, could be in either direction depending on the concentrations of Na^+^, K^+^, and Cl^−^ on the two sides of the membrane: for the concentrations in Table 1 transport is outward as shown. The electrical potential inside the cells is substantially negative while the CSF is somewhat positive relative to the fluid on the blood side of the epithelium. The source of the current that maintains the potential in the CSF may be the blood–brain barrier (see Sects. 3.7, 6.4 with its associated footnotes)

Studies on the expression of specific transporters at the RNA and protein levels in adults have been reviewed recently [4, 168]. It has been found in these studies that ion transporters are expressed in choroid plexus epithelial cells at levels sufficiently high to allow clear detailed cellular localization by immunohistochemistry as illustrated in Fig. 5. Figure 6 indicates the transporters present together with the ions they transport. From the known properties of these transporters together with careful measurements of electrical potentials and currents and from the results of techniques such as gene knockout, it is possible to describe the main features of solute transport involved in secretion as shown in the figure and described in Sect. 3.6. More extensive discussion and detailed referencing can be found in reviews by Brown et al. [169] and Damkier et al. [4].

### Summary of mechanisms for the principal species transported

The following sections are based on the scheme shown in Fig. 6, which is derived from the description and figures presented by Damkier et al. [4]. Damkier et al. should be consulted for discussion of the supporting evidence. The ion concentrations in choroid plexus found by Smith et al. [170] and Johanson and Murphy [171] are compared with those in CSF and plasma in Table 1. Of particular note are the relatively high [Na^+^]_CPcell_ and low [K^+^]_CPcell_.

**Table 1:** Ion concentrations and pH in choroid plexus, CSF and plasma of adult rats

	Ion concentrations (mmol per litre of H_2_O)			
	LV	4th V	CSF	Plasma
Na^+^	49–53 46*	55–58 56*	152–156	148–155
K^+^	95–97	87–89	2.99–3	3.9–4.6
Cl^−^	62–64 67*	63–64 67*	126–129	113–114
HCO_3_^−^	(11)	(11)	22–24	21
pH	7.06	7.04	7.33	7.44

*LV* lateral ventricle; *4th V* 4th ventricle

Data from [171] except values marked * from [170]. Values of [HCO_3_^−^] in parentheses were calculated from pH and pCO_2_

#### Pathways of Na^+^ transport

As with other secretory epithelia, the main driving force for fluid movement across the choroid plexus is provided by the Na^+^, K^+^-ATPase or Na^+^-pump. This actively transports Na^+^ out of the epithelial cells into CSF [144, 172] so reducing intracellular [Na^+^] and providing a gradient for Na^+^ influx via other transporters. Na^+^ entry from the blood side is thought to occur primarily by the Na^+^, HCO_3_^−^ cotransporter, NBCn2/NCBE. Some Na^+^ leaves the cell towards the CSF via NBCe2 (sodium bicarbonate electrogenic transporter number 2) driven outward by the coupled outward flux of 3 HCO_3_^−^ ions. (The free energy available from movement of 1 Na^+^ down its concentration gradient into the cell can carry 2 HCO_3_^−^ inwards whereas it takes movement of 3 HCO_3_^−^ out of the cell to provide the free energy needed to shift 1 Na^+^ out of the cell.) The direction of the Na^+^ flux via NKCC1 is not known with certainty (see Sect. 3.6.4): if the concentrations shown in Table 1 are correct it is outward. Although Na^+^-linked amino acid transporters are present in the apical membrane they are ignored here because their relative contribution to Na^+^ transport is thought to be small.

Na^+^ can also cross the epithelial layer via the paracellular route through tight junctions. The likely direction of the net flux from CSF to blood is indicated in Fig. 6. The claudins present in these tight junctions are expected to allow passive movement of small univalent cations and water. Thus there should be observable tracer fluxes of Na^+^ in each direction but with a net paracellular flux that is smaller than the transcellular movements. Paracellular fluxes are considered further in Sect. 3.6.4 in connection with K^+^ transport. Studies on the relative sizes of the unidirectional versus net fluxes across the choroid plexuses as a whole have produced discordant results, but it is likely that as in other leaky epithelia the unidirectional flux from blood to CSF substantially exceeds the net flux.^6^

So long as water moves easily enough to allow newly secreted CSF to be nearly isosmotic (slightly hyperosmotic with plasma, see Sect. 3.4), regulation of the rate of net Na^+^ transport is almost equivalent to regulation of the rate of CSF fluid secretion (see discussion of electroneutrality and constant osmolality in Sect. 6).

##### Is an amiloride-sensitive ion channel involved in Na^+^ transport at the choroid plexus?

Evidence for the presence of an amiloride-sensitive Na^+^ channel at the choroid plexus is conflicting. In an early study Davson and Segal [173] observed that amiloride could inhibit CSF production. However, this was seen only when it was infused at high concentration (~1.5 mM) directly into a carotid loop. Subsequently Murphy and Johanson [174] also detected inhibition with amiloride but noted explicitly that the concentration of amiloride needed (near 120 µM) was larger than required for specific inhibition of Na^+^ channels. They concluded that amiloride was acting by inhibiting a Na^+^/H^+^ exchanger rather than an epithelial Na^+^ channel, ENaC.

Histochemical techniques have also been used to look for ENaC channels in the epithelial cells. Leenen et al. [175, 176] reported the presence of alpha, beta and gamma subunits of ENaC in choroid plexus using antibodies developed by Masilamani, Knepper et al. [177]. However, these subunits appeared to be primarily in the cytoplasm and the apical brush border rather than the basolateral membrane where they would have been expected in order to mediate Na^+^ entry. In experiments to assess channel function, Leenen et al. measured ^22^Na^+^ uptake (which they called retention) into choroid plexus epithelial cells and found a decrease following application of benzamil, an amiloride derivative that is more selective at inhibiting ENaC. Based on these results they suggested that ENaC in the apical membrane might be involved in a regulated backleak of Na^+^ from CSF into the epithelial cells, which could be important in the control of secretion [178].

Others however have reported evidence against a role for ENaC. Millar and Brown, using the methods described in [179], could see no evidence of an amiloride-sensitive current. Their unpublished experiments showed that amiloride at a relatively low concentration of 10 µM (but which is still 50–100 fold greater than the expected IC_50_ for inhibition of ENaC) had no effect on the residual current (current in the absence of K^+^ and with reduced Cl^−^ currents): the conductances measured in the absence and presence of amiloride were 10.5 ± 1.4 pS/pF and 9.5 ± 0.9 pS/pF (n = 11) respectively, ([4] and Brown, personal communication). Furthermore Praetorius using his own antibodies could find beta and gamma subunits of ENaC in the choroid plexus but he was unable to confirm the presence of the alpha subunits needed for formation of ENaC channels [4].

Regardless of the resolution of this matter, there is no evidence that ENaC provides a basolateral route of entry for Na^+^ that would contribute towards secretion of fluid. The effect of amiloride observed at high concentration might conceivably be on the permeability of the paracellular pathway as suggested by Wright [180]. This possibility is discussed in more detail for the blood–brain barrier in Sect. 4.3.4.

#### Pathways of HCO_3_^−^ and Cl^−^ transport

**Fig. 7:**
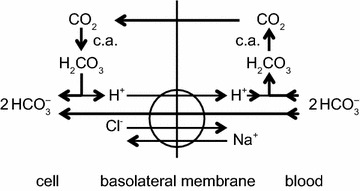
Net transport by the Cl^−^-dependent Na^+^, HCO_3_^−^-cotransporter, NCBE, in the basolateral membrane of the choroid plexus epithelial cells would be equivalent to the transport of 1 Na^+^ and 2 HCO_3_^−^ into the cell and 1 Cl^−^ out. Carbonic anhydrase catalyzes the steps indicated by c.a.

The most important anions in CSF secretion are HCO_3_^−^ and Cl^−^. HCO_3_^−^ enters the choroid plexus epithelial cells via the transporter known as either NBCn2 or NCBE (see Fig. 6). If, as indicated by the solid arrows, this transporter operates with stoichiometry of 1 Na^+^ and 1 HCO_3_^−^ moving inwards the name NBCn2 (sodium bicarbonate neutral transporter number 2) is appropriate. Alternatively if, as indicated by solid and dashed arrows, 1 Na^+^ and 1 HCO_3_^−^ move inwards and 1 H^+^ and 1 Cl^−^ outwards the name should be NCBE (sodium driven chloride bicarbonate exchanger) which effectively loads the cell with 2 HCO_3_^−^ for each Na^+^ transported. As discussed in [4], the rat gene when expressed in mouse NIH-3T3 fibroblasts behaves as Ncbe [181] while the human gene when expressed in *Xenopus laevis* oocytes behaves as NBCn2 [182]. It may be that the mode of operation is determined by the type of cell in which the gene is expressed or by the species of the gene. If it is the type of cell that is important, then expression of the human gene in human cells may produce NCBE, which is favoured by the present functional evidence (see Sect. 3.6.3). As shown in Fig. 7 with the transporter in the NCBE mode the H^+^ exported can be thought of as originating from CO_2_ conversion catalysed by carbonic anhydrase to H_2_CO_3_, which dissociates to H^+^ and HCO_3_^−^. Outside the cell the exported H^+^ reacts with HCO_3_^−^, again catalysed by carbonic anhydrase, leading to the formation of CO_2_. The net effect of one cycle of NCBE in the direction shown together with movement of CO_2_ would be influx of 1 Na^+^ and 2 HCO_3_^−^ and efflux of 1 Cl^−^.

HCO_3_^−^ leaves the epithelial cell into CSF via NBCe2 as indicated in Fig. 6 and possibly via anion selective channels. The ratio of the amounts of HCO_3_^−^ and Na^+^ in CSF is close to ~1:6, but that in newly secreted CSF is somewhat higher (see [183, 184] and for further references [185]). Coupling of Na^+^ entry via its major route to import of 1 or even 2 HCO_3_^−^ ions per Na^+^ ion means that far more HCO_3_^−^ enters the cell on the basolateral, blood side than appears in CSF. As indicated above the rest is thought to be recycled across the basolateral membrane via AE2, which mediates exchange of HCO_3_^−^ for Cl^−^.

Regulation of HCO_3_^−^ transport across the choroid plexus and its interrelations with H^+^, CO_2_ and Cl^−^ transport are considered in Sect. 6.

The transport of Cl^−^ is inextricably linked to that of HCO_3_^−^ because the only known route for net entry of Cl^−^ to the epithelial cells from the blood is an exchange of Cl^−^ for HCO_3_^−^ via AE2 (see Fig. 6). In the scheme shown, there is a ready supply of HCO_3_^−^ for exchange as the major route for Na^+^ entry is by cotransport with HCO_3_^−^. If the cotransporter is NBCn2, then a large proportion of the HCO_3_^−^ that enters would be recycled by AE2 and thus the combined effect of the NBCn2 and AE2 would be entry mainly of NaCl with a smaller amount of NaHCO_3_. If alternatively the cotransporter is NCBE, then AE2 must exchange a much larger amount of HCO_3_^−^ for Cl^−^ as it must transport enough Cl^−^ to supply both the amount secreted and the amount transferred out of the cells across the basolateral membrane by NCBE. A small amount of Cl^−^ returns to the blood via KCC3. The combination of DIDS (4,4′-diisothiocyanostilbene-2,2′-disulfonic acid), which inhibits AE2, and bumetanide, which inhibits NKCC1, reduces ^36^Cl^−^ uptake into isolated choroid plexus by 90% [186].

Cl^−^ efflux from the epithelial cells appears to involve both transporters and channels [187]. On the apical side Cl^−^ is likely to leave the epithelial cells to the CSF by cotransport with K^+^ mediated by KCC4 and via anion channels that have been observed functionally but whose molecular identities are as yet unknown (see [4]). NKCC1 has been shown to mediate large tracer fluxes in both directions. If the ion concentrations in CSF and within the epithelial cells are those shown in Table 1, then NKCC1 must also be a route for net outward Cl^−^ flux [188] (see Sect. 3.6.4).

The indirect coupling of Cl^−^ fluxes to those of Na^+^ via the combination of NBCn2/NCBE and AE2 provides an explanation for the observation that net Cl^−^ transport across the epithelium can be against its electrochemical gradient [189].

#### Role of carbonic anhydrase in HCO_3_^−^ transport

Carbonic anhydrase is important in many secretory/absorptive epithelia both for hydration of CO_2_ to form H_2_CO_3_ and for the reverse reaction. In the choroid plexus the soluble CAII isoform is known to be present within the epithelial cells and there are also membrane bound isoforms present on both basolateral and apical membranes [190–193]. Carbonic anhydrase is very likely to be involved somehow in CSF secretion because secretion is inhibited at least 50% by acetazolamide [16] and the only established action of acetazolamide is to inhibit carbonic anhydrase (possible effects on AQP4, but not on the AQP1 found in the choroid plexus, are considered in 7). How carbonic anhydrase is involved in CSF secretion is less clear.

From consideration of the transporters shown in Fig. 6, if NBCn2/NCBE imports NaHCO_3_ without export of HCl, i.e. it operates as NBCn2, hydration/dehydration is not required for transport of HCO_3_^−^ and there is no obvious role for carbonic anhydrase in this transport. However, if NBCn2/NCBE works both to import HCO_3_^−^ and to export H^+^, i.e. it operates as NCBE, then there would be an obvious role for carbonic anhydrase because hydration and dehydration are needed to supply and remove the protons at an adequate rate (see Fig. 7). Thus the available functional evidence favours operation as NCBE rather than NBCn2. A controversial alternative possibility is that carbonic anhydrase is present at the membranes of the epithelial cells as part of transport metabolons (complexes of two or more proteins at least one of which is a transporter) [194, 195]. If so, and carbonic anhydrase binding to the transporter modifies the transport function, then acetazolamide might by binding to the carbonic anhydrase inhibit the function of the transporter, accounting for the reduction in secretion.

#### Pathways for K^+^ transport

The activity of the Na^+^-pump loads K^+^ into the epithelial cells from the CSF. All other routes for K^+^ transport mediate net K^+^ efflux or in the case of NKCC1 the direction of transport is finely balanced (see below and [4] for references and discussion). Almost but not quite all of the K^+^ that enters the epithelial cells from the CSF is recycled to the CSF via some combination of KCC4, K^+^ channels and NKCC1 (see below), all of which are known to be present in the apical membrane (see Fig. 6).

The only known route of transfer of K^+^ across the basolateral membrane is KCC3 which mediates a net efflux of K^+^ from epithelial cells towards blood, i.e. [K^+^]_in_[Cl^−^]_in_/([K^+^]_out_[Cl^−^]_out_) > 1. This indicates outward transport since ratios > 1 indicate a net outward driving force while those <1 indicate inward. Net efflux across the basolateral membrane implies that the net flux across the epithelium by the transcellular route must be towards blood. This being so, because newly secreted CSF contains K^+^, this ion must get into CSF across the choroid plexus by another route. This is presumed to be the paracellular pathway with K^+^ passing through the tight junctions and lateral spaces between the epithelial cells. The conditions to allow this are met because the tight junctions in the choroid plexus are leaky to monovalent cations and the electrochemical gradient for K^+^ will drive K^+^ in the direction of the CSF.

NKCC1 in the apical membrane has been shown to mediate large tracer fluxes of Rb^+^ [188]. However, while studies in isolated choroid plexus have found that it mediates net efflux [188], other studies focussed on epithelial cell volume strongly imply that NKCC1 mediates net influx [196, 197]. The results may all correctly reflect the circumstances in which they have been measured because the net driving force, derived from the concentrations of Na^+^, K^+^ and Cl^−^, is finely balanced and NKCC1 could be transporting in either direction [4, 188]. For the concentrations in Table 1, the ratio $\frac{{\left[ {{\text{Na}}^{ + } } \right]_{\text{cell}} \left[ {{\text{K}}^{ + } } \right]_{\text{cell}} \left[ {{\text{Cl}}^{ - } } \right]_{\text{cell}} }}{{\left[ {{\text{Na}}^{ + } } \right]_{\text{CSF}} \left[ {{\text{K}}^{ + } } \right]_{\text{CSF}} \left[ {{\text{Cl}}^{ - } } \right]_{\text{CSF}} }}$ is 2.6, which indicates outward transport.

It has been suggested [198, 199] that the presence of both the Na^+^-pump and NKCC1 in the apical membrane [188] allows an uncoupling of Na^+^ and K^+^ fluxes. Thus increased [K^+^]_CSF_ could stimulate flux of K^+^ from CSF into the choroid plexus cells (or inhibit that in the opposite direction) by both of these transporters while increasing Na^+^ efflux from the cells via the pump but favouring Na^+^ influx via NKCC1. This would allow K^+^ transport to be changed without disturbing the net transport of Na^+^. It is, however, unlikely that this is the complete story because raised CSF K^+^ concentration is also likely to change the net flux of K^+^ through KCC4 and K^+^ channels in the apical membrane (see Fig. 6).

The [K^+^] of newly formed CSF is remarkably stable in the face of changes in plasma [K^+^] with the CSF concentration remaining at ~3.5 mmol kg^−1^ as plasma concentration increases from 4 to 9 mmol kg^−1^ [141, 200]. As discussed in Sect. 5 how this is achieved is only partially understood.

### Electrical current and potential difference across the choroid plexus

Ion transfer via each of the known transporters in the basolateral membrane is electrically neutral and no ion channels have been localized to this membrane [4]. If this is correct and no charge carrying mechanisms have been missed, there can be no net transcellular current and the conductance of the epithelium is determined by the permeability of the paracellular pathway through the tight junctions (see Sect. 6.4 and associated footnote for further discussion).

Ion transfer across the apical membrane via many of the transporters and channels is associated with net movement of charge. Thus the rates of transfer across this membrane should be sensitive to the apical membrane potential and the value of the potential difference across this membrane should be the value that preserves electroneutrality of the cells. Electroneutrality is discussed in Sect. 6.

## Ion and fluid transport at the blood–brain barrier

The primary functions of the blood–brain barrier are to allow ready access to the brain parenchyma of O_2_ and nutrients such as glucose and essential amino acids and ready removal from the brain of waste products like CO_2_, while at the same time providing a barrier to the movement of substances that should not be allowed to enter or leave the brain. The blood–brain barrier also plays the principal role in long-term regulation of the ionic composition of ISF. Although astrocytes are very important in short-term control of ISF ionic composition, a process sometimes called physiological buffering, they cannot set or determine the long-term composition (see e.g. Sect. 5).

Unfortunately, it has not been possible to determine the composition of the fluid, if any, secreted by the blood–brain barrier by direct sampling (compare Sect. 3.1). However, if the fluid secretion rate were known, one could infer the composition because the net fluxes of solutes and water across the blood–brain barrier plus the water produced by brain cell metabolism must replace the fluid that is lost by net outflow from the parenchyma after allowance for metabolic changes (see sections 1.4 and 4 in [15]). The fluid lost is a nearly isosmotic solution with composition very close to that of CSF. Thus the net fluid transferred across the blood–brain barrier into the brain must be either a hyperosmotic secretion or a hypoosmotic absorbate to make the net product, including the ~60 ml day^−1^ of metabolic water, nearly isosmotic (see Sect. 4.7 and 8).

### Evidence for and against fluid secretion by the blood–brain barrier

The most widely quoted value for blood–brain barrier secretion rate, 10–20% of that by the choroid plexuses, was based on washout of markers from the brain parenchyma with half-times of 6–12 h (see e.g. [201, 202] and discussion in sections 3.2 and 4.1.1 of [15]). However, this estimate has been called into question on two grounds. Firstly, the experiments were conducted under barbiturate anaesthesia, which has subsequently been shown to reduce the washout rate [203] (discussed in section 4.1.1 in [15]). This inhibition by barbiturate would have led towards underestimates of the washout rate by as much as sixfold, and thus, based on washout evidence alone, the blood–brain secretion rate could even be as large as the rate of production of CSF by the choroid plexuses. Secondly, the washout of marker might be caused by fluid arising from a source other than the blood–brain barrier. It has been suggested that such a source may be periarterial influx of CSF into the parenchyma (reviewed in [204] and [15]). This periarterial influx combined with perivenous efflux of fluid was originally proposed to explain data for the distribution of horseradish peroxidase [205]. Recently it has been championed, based on evidence obtained using in vivo imaging of the movements of fluorescent tracers, and has been termed the glymphatic circulation [206]. As discussed at length in [15] the glymphatic hypothesis is intriguing, raises important issues and explains key qualitative features of movements of substances into and out of the parenchyma. However, it is still lacking in both quantitative detail and explanations for some aspects of the data. (For example, what induces NaCl to move from the periarterial spaces, into the interstitium and then into perivenular spaces?) It is premature to describe “the glymphatic circulation” as a proven fact (see [21, 207–211] for critical views).

The effect of ignoring *net* periarterial influx of CSF, if it exists, would be to make too large the estimates based on washout data of the secretion rate at the blood–brain barrier (see sections 4.3 and 5 in [15]). No estimates are available for the magnitude of net flows by any perivascular route and thus such flows might account for all or none of the washout of markers. As a consequence of the uncertainties related to the use of barbiturate anaesthesia and to the magnitude of the flow of CSF into the brain parenchyma, other sources of evidence are needed to provide an estimate of the rate of secretion of fluid across the blood–brain barrier.

**Fig. 8:**
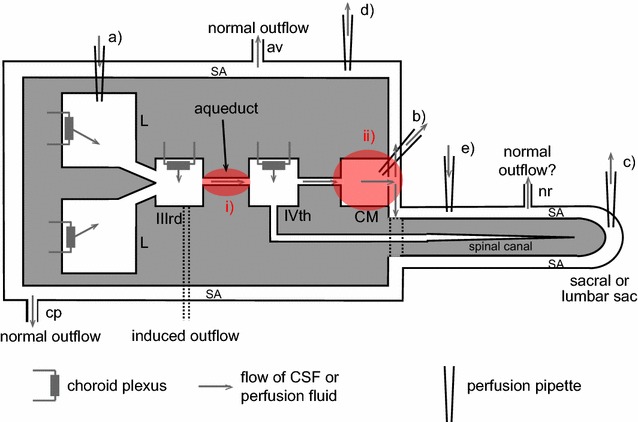
Schematic diagram of brain structures, CSF flows and perfusion pipette positions related to the perfusion studies and other investigations discussed in this section. Most of the CSF is produced by the choroid plexuses located in the lateral (L), IIIrd and IVth ventricles. Net CSF flow then normally proceeds through the cisterna magna (CM) to the subarachnoid spaces (SA), which for this purpose include the basal cisterns. Outflow from the brain occurs via a number of routes including perineural routes through the cribriform plate (cp), the arachnoid villi (av), perineural pathways at roots of nerves (nr) including those in the spinal cord, and, in addition, perivascular routes and dural lymphatics that are not shown [543, 544]. Any fluid secreted by the blood–brain barrier within the parenchyma can flow into CSF in the subarachnoid spaces or leave the brain by perivascular and perhaps perineural pathways without first mixing with the CSF that is sampled at the cisterna magna. Flows are investigated using a number of perfusion techniques. In ventriculo-cisternal perfusion, fluid is injected via a pipette or cannula at (*a*) and withdrawn at (*b*). For ventriculo-lumbar perfusion the withdrawal is at (*c*) while for ventriculo-subarachnoid perfusion at *d*. For spinal perfusion fluid is injected at (*b*) or (*e*) and withdrawn at (*c*). In non-communicating hydrocephalus as discussed in this review, the aqueduct connecting the IIIrd and IVth ventricles is blocked as indicated at (*i*). In hydrocephalus induced by injection of kaolin into the cisterna magna the block is at the cisterna magna and at its connections to the IVth ventricle and the subarachnoid spaces as indicated at (*ii*). The causative pathology in communicating hydrocephalus is unknown but outflow of CSF is somehow hindered (see Fig. 9). In kaolin induced hydrocephalus the major escape route for CSF is now thought to be along the spinal canal, through spinal parenchyma to the subarachnoid space and out via the nerve roots. In non-communicating hydrocephalus (point 3i) and possibly in communicating hydrocephalus (points 3iii) there is a route of escape of CSF from the lateral and IIIrd ventricles, indicated in the diagram as being from the IIIrd ventricle

There are six types of evidence that can be used in arguments for or against blood–brain barrier secretion of fluid. The various structures and flows discussed are indicated schematically in Fig. 8.

**Fig. 9:**
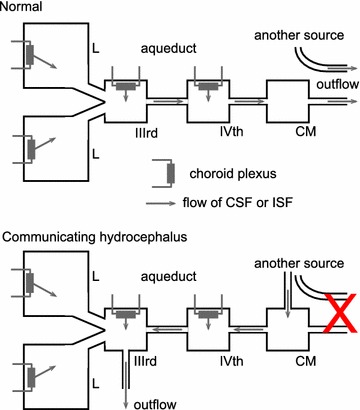
A schematic diagram of one interpretation of the differences in CSF flow in normal subjects and those with communicating hydrocephalus. In normal subjects, CSF is secreted by the choroid plexuses into the lateral (L), IIIrd and IVth ventricles. Some fluid is also secreted into the parenchyma, presumably by the blood–brain barrier. The magnitude of the net flow through the cerebral aqueduct is close to the sum of the secretions into the lateral and IIIrd ventricles. Fluid passes through the IVth ventricle and cisterna magna (CM) on its way to routes of outflow from the brain, i.e. via arachnoid villi, nerve tracts through the cribriform plate, and both perivascular and perineural pathways. At least some of these routes allow exit of fluid from the parenchyma without it ever mixing with CSF in the large cavities. In communicating hydrocephalus there is some deficit in the normal route of outflow indicated by the *red* X. The observation of reversed net flow through the cerebral aqueduct implies that another source of fluid enters CSF at some point, shown in the diagram as the cisterna magna, and, when combined with the secretion from the choroid plexus in the IVth ventricle, it equals the flow through the aqueduct. Some other pathway allowing fluid to escape from the ventricles must exist perhaps emerging from the IIIrd ventricle as shown. One possibility is flow through swollen periventricular parenchyma eventually reaching an exit route, perhaps either perivascular or perineural. Fluid exit via absorption across the blood–brain barrier is unlikely because this would require substantial alteration of barrier properties (see Sect. 5.1 with the caveats in sections 3.2 and 2.7 in [15]

1. *Observation*: if fluid is perfused through the cerebral aqueduct more comes out than goes in [212, 213].* Interpretation*: the extra fluid that crosses the ependyma lining the aqueduct must have originated somewhere and the obvious suggestion is the blood–brain barrier in the surrounding parenchyma. However, consideration needs to be given to the possibility that it might be recirculation of CSF entering the parenchyma from the subarachnoid spaces via periarterial pathways.
2. *Observation*: 3–9 months after destruction of 80–90% of the choroid plexuses in rhesus monkeys CSF production, measured by ventriculo-cisternal perfusion, is as much as 60% of the normal rate [214] (see also [215, 216] and sections 3.1.1 and 3.2 in [15]). These results have never been convincingly explained on any basis other than extrachoroidal secretion of fluid. This evidence coincides with the general clinical experience that it is difficult to alleviate hydrocephalus using choroidectomy alone which partially explains the ascendancy of shunt placement as a treatment [215, 217–219] but see [2].* Interpretation*: there is a source of CSF in addition to the choroid plexuses.
3. A third type of evidence has arisen from studies of the distribution and flow of CSF in hydrocephalus. These studies have used measurements of ventricular volumes, perfusion techniques to measure production and absorption of CSF and more recently phase contrast magnetic resonance imaging (PC-MRI) to monitor CSF flow. In the interpretation of these it is necessary to consider the sites of CSF outflow as well as those of CSF production. The arguments are summarized in the following paragraphs. For elaboration see Fig. 8, 9 and previous discussion in sections 4.2.1.1–4.2.2.2 of [15].
  i. *Observation*: when the cerebral aqueduct is blocked, indicated at (i) in Fig. 8, CSF escapes from the lateral and third ventricles.* Interpretation*: to do this it must pass by some means other than the cerebral aqueduct to a site for outflow [220, 221]. There is independent evidence that such routes exist, at least for sucrose [222].
  ii. *Observation*: when the cisterna magna and connections from the IVth ventricle to the subarachnoid space are blocked following to kaolin injection, there is diversion of CSF from the IVth ventricle into the central canal of the spinal cord leading to alternative sites of outflow [223, 224].* Interpretation*: because in kaolin hydrocephalus the outflow originates from the IVth ventricle, this model cannot be used to describe the swelling of the lateral and IIIrd ventricles in non-communicating hydrocephalus in which the aqueduct is blocked (see Fig. 8).
  iii. *Observation*: in communicating hydrocephalus there are indications from movements of impermeant markers [225–227] and from flow measurements by PC-MRI [228–230] (but see [231]) that there is reversed net flow of CSF through the cerebral aqueduct (see Fig. 9).* Interpretation*: if this is correct, there must be a source of the fluid that flows from the IVth to the IIIrd ventricle. That from the choroid plexus in the IVth ventricle is not enough (see also footnote 9) and the plausible source of the extra fluid is the blood–brain barrier. In addition as in non-communicating hydrocephalus, there must be a route from the lateral or IIIrd ventricles to a site of outflow from the brain. The PC-MRI experiments on net flow in communicating hydrocephalus remain controversial and should be revisited (see section 4.2.2.2 in [15], [231] and see also footnote 9).
4. *Observation*: spinal perfusion studies and comparisons of ventriculo-lumbar and cisternal-lumbar perfusions (see Fig. 8) have been used to estimate spinal formation and absorption of CSF. Estimates of spinal CSF formation are in effect based on the dilution of an impermeant marker as the fluid travels the length of the cord while those for absorption are based on the difference between the amounts of marker infused and recovered. In cats, dogs and rhesus monkeys [232–236] fluid absorption was easily demonstrated but these studies failed to detect spinal CSF formation.
*Interpretation*: these studies put an upper limit on the rate of ~1 µl min^−1^ which has led to the general view that there is little or no secretion of fluid in the spinal cord. To put these studies into perspective it is necessary to have an estimate of how large the rate of secretion into the spinal cord would be if there were a functionally important secretion across the blood–brain barrier: 200 ml day^−1^ or 0.1 µl g^−1^ min^−1^ is a high estimate of the secretion rate in humans with a 1400 g brain. The mass of the spinal cord of a human is only 35 g, which leads to an estimate of 3.5 µl min^−1^ for secretion into the spinal cord. The dogs, cats and rhesus monkeys used in the perfusion studies are about 10× smaller than humans which suggests that the secretion rates into the spinal cord would also be much smaller. *None of the existing studies looking for secretion of fluid into the spinal cord have been sufficiently accurate to detect the secretion that might be expected.*
5. *Observation*: the tracer influx of Na^+^ over the entire blood–brain barrier is as large as that across the choroid plexus in rats and rabbits (see Sect. 4.3.2) and is substantially larger than the net flux required for even the largest estimates of blood-barrier fluid secretion rate (see below and Sect. 4.3.5). Tracer efflux has also been measured (see Sect. 4.3.2 and associated footnote) and is similar in size to the influx.* Interpretation*: the difference between influx and efflux is so inaccurate that it cannot be used in any argument for or against the existence of a net influx of Na^+^ (and hence fluid secretion). However, because efflux is not clearly smaller than influx, and the Na^+^ influx is not much greater across the blood–brain barrier than across the choroid plexuses, the net Na^+^ flux across the blood–brain barrier is likely to be less than the net Na^+^ flux across the choroid plexus and the same will apply to the rates of fluid secretion. More accurate data are required before this argument can be made quantitative (see Sects. 4.3.2 and 4.3.5).
6. *Observation*: inhibitors of ion transporters found at the blood–brain barrier reduce the rate of development of focal oedema [19, 237–240] as if they are inhibiting fluid secretion into the region.* Interpretation*: it should be borne in mind that these same ion transporters are also found at other sites within the brain and thus the inhibitor effects on fluid accumulation might be indirect [241]. Nevertheless at present it appears that these effects are evidence that there can be secretion across the blood–brain barrier.^10^

From the in vivo evidence and the arguments considered above, there is a strong but still far from conclusive case that the blood–brain barrier normally secretes fluid. While the amounts secreted across the blood–brain barrier and choroid plexus may be of the same order, the blood flows from which they are derived differ by almost 100-fold. It is thus not at all surprising that arterio-venous concentration differences for impermeant substances can be measured for the choroid plexuses [148, 242] but not for the blood–brain barrier.

#### Net fluxes of inorganic ions across the blood–brain barrier

The net transfers of inorganic ions across the blood–brain barrier are small. These long-term average net fluxes can be calculated if the net rate of fluid loss from the parenchyma is known. Taking for example 200 ml day^−1^ (towards the upper end of currently plausible guesses) it is possible to calculate the size of the ion transfers that would be entailed. The fluids leaving the parenchyma are thought to have the concentrations of Na^+^, K^+^, Cl^−^ and HCO_3_^−^ and total osmolality similar to those in CSF. On this basis the net transfer of Na^+^ across the blood–brain barrier is only 0.15 mol l^−1^ × 0.2 l day^1^ = 30 mmol day^−1^. This is substantially less than the Na^+^ movement into the endothelial cells associated with amino acid reabsorption, 145 mmol day^−1^ (see Sect. 2.4.2). Therefore the endothelial cells of the blood–brain barrier are certainly capable of active transport of sufficient Na^+^ to support secretion at this rate. Furthermore the small size of the net ion transport entailed in fluid secretion explains the “heroic efforts” [21] needed to detect the ion fluxes and the expression of the transporters that mediate them.

As will be described in the following sections, all the molecular components needed for secretion of ions and fluid are present at the blood–brain barrier. In addition there is an energy source which could drive secretion as the number of mitochondria in the endothelial cells of the blood–brain barrier is relatively high, sufficient to occupy 5–10% of the cell volume [1, 243]. What is lacking is conclusive evidence that the transporters are appropriately organized and function together so as to achieve a net secretion of fluid. For a recent, emphatic statement of the view that the blood–brain barrier secretes very little, if any, fluid see [2, 21] but see also Sect. 4.3.3 for a critique of part of the basis of that view.

*Caveat: while most of this section is written as if net secretion does take place across the blood*–*brain barrier, it must be kept in mind that this has not been proven.*

### Hydrostatic pressure gradients cannot be responsible for significant fluid movement across the blood–brain barrier

Hydrostatic pressure differences could, at least in principle, drive fluid movements between blood in the microvessels and ISF as indeed they do between blood and peripheral tissues. Such a mechanism has been proposed variously to explain movements of fluid either into or out of the brain (for references see [15]). However, there is a large difference between microvessels in the brain and those in the periphery in that the former have much lower permeability to Na^+^ and Cl^−^ (see Sect. 4.3.2). Thus any pressure forcing water across the blood–brain barrier would leave solute behind and wash solute away on the brain side. The developing solute concentration difference would produce an osmotic pressure sufficient to stop water flow long before the concentration difference would become sufficient to drive the solute across the barrier. This is in complete contrast to the situation for capillaries and venules in peripheral tissues. The permeabilities to Na^+^ and Cl^−^ are there so large and the resulting differences in [Na^+^] and [Cl^−^] so small that the osmotic pressure differences resulting from them are much smaller than the hydrostatic and colloid osmotic pressure differences. Net fluid movements across peripheral microvessel walls are well described by the Starling mechanism in which transfers of water and small solutes are driven by differences in the hydrostatic and colloid osmotic pressures. At the blood–brain barrier there must be transport of solutes with water following either by simple diffusion through the lipid bilayers of the endothelial cell membranes or via specific proteins, perhaps GLUT1 (see Sect. 4.3.6,^11^ and section 2.7 of [15]).

### Functional evidence for ion transport at the blood–brain barrier from in vivo (and ex vivo) studies

In vivo techniques have been used to measure tracer influxes of ions into the brain and sometimes with more difficulty tracer effluxes. However, it has not been possible to measure net fluxes. That the transfers are taking place across the blood–brain barrier has had to be inferred from measurements of changes in the content of parenchyma, extracellular plus intracellular, and allowance for exchanges between the ISF and CSF. Discussion of in vivo results for K^+^ and Na^+^ are given below. Evidence for movements of HCO_3_^−^ is inextricably linked to consideration of pH and discussion is postponed until Sect. 6.

#### Results mainly concerned with K^+^ movement

It was established in early studies that [K^+^]_CSF_ is less than would be the case if it were at equilibrium with [K^+^]_plasma_ and the potential difference between plasma and CSF. Thus for [K^+^]_plasma_ = 4.6 mM [17] and a potential difference of 4 mV CSF positive (see Sect. 6.4 and its footnotes), the equilibrium value of [K^+^]_CSF_ would be 3.9 mM (calculated using the Nernst equation) while the measured value in the cisterna magna is less than 3 mM [17]. Because [K^+^]_ISF_ is closely similar to or less than [K^+^]_CSF_ [244–247] there must be an active process maintaining lower concentrations of K^+^ in CSF and ISF than in plasma [17, 244]. Bito et al. took these results to imply that K^+^ must be actively transported from ISF to blood across the blood–brain barrier [244]. However, while such active transport may be the correct explanation, the lack of equilibrium is not enough to imply the existence of a net, active flux of K^+^ from ISF to plasma. If there is a sufficient net secretion of fluid, including K^+^, across the blood–brain barrier, then [K^+^]_ISF_ will be the same as the concentration in the secreted fluid (after dilution with metabolic water), which could easily be lower than in plasma [241, 248].

The earliest tracer studies on K^+^ movement established that ^42^K^+^ added to blood appears rapidly in CSF [249] but slowly in the brain parenchyma [250]. The long half-time for penetration into brain (10–20 h) results because the K^+^ content of the brain is very large and the permeability of the blood–brain barrier to K^+^ is much lower than that of peripheral capillaries. K^+^ in the blood can enter the parenchyma either across the blood–brain barrier or indirectly by CSF secretion across the choroid plexus followed by diffusion into the parenchyma. A substantial amount may enter via the latter route. In the ventriculo-cisternal perfusion experiments of Cserr it was observed that more ^42^K^+^ left the ventricles by diffusion into the parenchyma than by the outflow of CSF [251]. But, nevertheless Katzman [252] calculated from the penetration rates into the parenchyma from CSF and from blood that approximately 4/5ths of K^+^ entry to the brain was actually across the blood–brain barrier rather than via the choroid plexuses.

Control of [K^+^]_ISF_ in the face of long-term changes in [K^+^]_plasma_ was investigated by Bradbury and Kleeman [253] who found that the rate of [K^+^] influx (measured with ^42^K^+^) increased with [K^+^]_plasma_ as if there were two components of influx, one at a rate independent of [K^+^]_plasma_ the other proportional to [K^+^]_plasma_. At normal [K^+^]_plasma_, 3.5–4 mM, the two components were almost equal. Despite the increase in influx, the amount of K^+^ in the brain showed no variation with [K^+^]_plasma_ (see Sect. 5). They concluded that “the larger volume of brain tissue relative to that of CSF, and the remoteness of parts of the brain from ventricular or subarachnoid CSF make it extremely unlikely that the control of the [K^+^]_ISF_ of the brain is secondary to control of the CSF by the choroid plexuses”. Furthermore they noted that maintenance of nearly constant [K^+^]_ISF_ requires some mechanism for increasing efflux from ISF to blood when [K^+^]_plasma_ is increased.

The principal proposal for how efflux of K^+^ is increased is still that presented by Bradbury and Stulcova [254]. Using ventriculo-cisternal perfusion they calculated ^42^K^+^ flux from brain to blood from the loss of ^42^K^+^ from perfusates with different [K^+^]. They found that the loss had two components. At low [K^+^], almost all of the loss was accounted for by uptake into the cells of the parenchyma with little efflux to blood, but as [K^+^] was increased the calculated rate of K^+^ efflux increased as a sigmoidal function of [K^+^]. Ouabain added to the perfusate at 10 µM inhibited the K^+^ efflux and lower [Na^+^]_CSF_ increased it. All of these findings are consistent with efflux of K^+^ from brain to blood being via the Na^+^-pump, and are reminiscent of the situation in red blood cells where the pumping rate is a sigmoid function of external [K^+^] [255] and is inhibited by ouabain and external [Na^+^] [256, 257]. Activation of the Na^+^-pump by external K^+^ in isolated cerebral microvessels has been shown to occur over the range of [K^+^] encountered in ISF [258].

Bradbury, Segal and Wilson [259] found that reducing [K^+^]_ISF_ by perfusing the subarachnoid space with K^+^ free solution, produced a 50% increase in the amount of ^42^K^+^ from plasma that accumulated in the parenchyma over a 2 h period. They suggested that over 2 h [^42^K^+^]_ISF_ may have increased sufficiently for there to be a substantial efflux of ^42^K^+^ from the brain which would be mediated by the Na^+^-pump. Because the relation between pump rate at the blood–brain barrier and [K^+^]_ISF_ is sigmoidal in the relevant range of concentrations, decreasing [K^+^]_ISF_ would decrease the pump rate for ^42^K^+^ which would increase the accumulation of ^42^K^+^ in the brain. Difficulties with this explanation and other possibilities are considered in 12.

From the data from many sources tabulated by Bradbury (Table 8.1 in [16]) the values for K^+^ permeability were generally about 0.5–0.7 ml h^−1^ g^−1^. (The data are expressed as the *PS* product, i.e. the product of permeability and area of barrier, usually per gram of tissue.) Keep et al. noted that K^+^ permeability in the adult rat would be inadequate to support a net influx of K^+^ in the foetus sufficient for brain growth. They found that the blood–brain barrier K^+^ permeability in rat foetus was much larger (2.5 ml h^−1^ g^−1^) [260]. Smith and Rapoport [261] measured entry of tracer K^+^ into regions of parenchyma far from the ventricles and hence not initially affected by entry via the choroid plexuses and CSF. They found permeabilities similar to the earlier values. They also compared influx into parenchyma and into CSF from which it can be concluded that for K^+^ the blood–brain barrier route is the dominant route of entry into brain [261] (see discussion for Na^+^ in the next section).

In a meeting abstract Ennis et al. [262] reported that 1 mM ouabain reduced ^86^Rb^+^ influx into in situ perfused rat brains by about 50%. If as expected [263] ouabain could only reach the luminal side of the blood–brain barrier from the perfusate this result argues that Na^+^-pump activity is present in the luminal membrane. Furthermore this route accounts for roughly half of the entry of K^+^ on this side of the endothelial cells (see Sect. 4.4.1 for further discussion of luminal Na^+^-pumps). This result contrasts with the premise that most of the K^+^ entry across the luminal membrane occurs via NKCC1 (see Sect. 4.5.1). NKCC1 has been localized to the luminal surface both in endothelial monolayers and in vivo (see Sect. 4.4.2). It would be very interesting to know the effect of the NKCC1 inhibitor bumetanide on K^+^ influx in the in situ perfused brain.

Further results comparing influx of K^+^ into CSF and the parenchyma and the changes in influx when [K^+^]_plasma_ is changed acutely or chronically are discussed in Sects. 5.1 and 5.2.

#### Results mainly concerned with Na^+^ movement

Davson and Segal [173] measured tracer flux into the brain parenchyma when ^22^Na^+^ was added to blood and found that this was not affected by ouabain, acetazolamide, or amiloride all of which had been shown to affect CSF secretion. From this they concluded that entry must be across the blood–brain barrier but that this measured ^22^Na^+^ influx did not represent a net flux. The tracer influx calculated from these and additional studies the following year by Davson and Welch [264] is indeed larger than any estimates of net flux that have been made (see Sect. 4.3.6). Unfortunately, they went on to conclude that there is no secretion (or absorption). This conclusion does not follow from their data. To reach any conclusion about the net flux of Na^+^ and fluid secretion requires measurements of both tracer influx and efflux, these measurements being sufficiently accurate to determine the difference between them. To date this has not been possible.^13^

Data from many sources for values for Na^+^ and Cl^−^ permeabilities, 0.08–0.19 ml h^−1^ g^−1^, were tabulated by Bradbury (Table 8.1 in [16]). Smith and Rapoport [261] found similar values and noted that following intravenous injection of tracers, the Na^+^ and Cl^−^ permeabilities of the blood–brain barrier were comparable to those of cell membranes, i.e. much greater than those of lipid bilayers but much smaller than those of leaky epithelia and peripheral capillary walls. The permeabilities to Na^+^ and Cl^−^ were similar to each other and to that of mannitol.

From their data obtained with rats, Smith and Rapoport [261] calculated the transfer constants for Na^+^ across the blood–brain barrier into samples of brain parenchyma, *k*_BBB =_ 2 × 10^−5^ s^−1^, and across the choroid plexus into CSF, *k*_CSF_ = 3.8 × 10^−4^ s^−1^. (The transfer constant for a solute is the ratio of the rate of transfer into unit volume of destination to the concentration at the source, which is calculated from experimental data for transfers into the brain or CSF as

1 $$ k = \frac{{\left[ {{{\left( {\text{tracer in sample}} \right)} \mathord{\left/ {\vphantom {{\left( {\text{tracer in sample}} \right)} {\left( {\text{volume of sample}} \right)}}} \right. \kern-0pt} {\left( {\text{volume of sample}} \right)}}} \right]}}{{\int\nolimits_{0}^{T} {[{\text{tracer}}]_{\text{plasma}} {\text{d}}t} }} $$

where *T* is the time allowed for influx.) The substantially larger value of the transfer constant for choroid plexus compared to that for the blood–brain barrier calculated by Smith and Rapoport has been cited as evidence of a “great difference in plasma ion penetration at the two barriers” (see p. 81 in [2]). However, this comparison is misleading. The transfer constants are rate constants for transfers into *unit volumes* of parenchyma or CSF. The comparison that is more revealing is between the rate for Na^+^ transfer into the whole parenchyma and rate for transfer into the entire volume of CSF. To obtain the rates for the total transfers it is necessary to multiply the transfer constants by the volumes of the respective destinations, ~1.7 cm^3^ for the brain and ~0.1 cm^3^ for CSF and by [Na^+^]_plasma_. This gives transfer rates of 3.4 × 10^−5^ cm^3^ s^−1^ [Na^+^]_plasma_ for the blood–brain barrier and 3.8 × 10^−5^ cm^3^ s^−1^ × [Na^+^]_plasma_ for the choroid plexuses.

The conclusion from these calculations for rats is that neither the choroid plexuses nor the blood–brain barrier can be ignored when considering influx of Na^+^ into the brain. Davson and Welch [264] found in rabbits that the time courses for tracer concentrations in brain water and CSF were similar. This implies that in rabbits substantially more Na^+^ enters the brain via the blood–brain barrier than via the choroid plexuses.

#### Results from further in vivo studies using inhibitors

The mechanisms that allow influx of Na^+^ into the brain across the blood–brain barrier have been investigated using inhibitors. Using such an approach Murphy and Johanson [265] confirmed that in vivo the carbonic anhydrase inhibitor acetazolamide inhibits secretion of Na^+^ at the choroid plexus but does not inhibit ^22^Na^+^ influx into brain across the blood–brain barrier. They also showed in contrast to Davson and Segal’s earlier observations [173] that amiloride, which inhibits Na^+^ transport in many epithelia, did produce a 22% inhibition of Na^+^ influx at the blood–brain barrier but at a relatively high dose, i.e. that calculated to produce a plasma concentration of 0.12 mM [174].

Betz [266], using an intracarotid bolus injection technique, compared Na^+^ uptakes over a range of [Na^+^] in the presence and absence of inhibitors. The uptake at a low [Na^+^], 1.4 mM, was found to have both saturable and unsaturable components. The saturable component was partly inhibited by 1 µM amiloride or 1 mM furosemide, which inhibit different Na^+^ transport mechanisms. These results suggested the presence of two distinct saturable transport systems. The amiloride-sensitive component was thought from its apparent *K*_*D*_ (no inhibition at 0.1 µM and maximal at 1 µM) to be an ion channel. The furosemide-sensitive component was thought to be a Na^+^, Cl^−^ cotransporter. The saturable components could be detected at low [Na^+^], but they could not be seen in tracer influx measurements conducted with more physiological [Na^+^], e.g. 140 mM.

Ennis, Ren and Betz [267] sought to improve the characterization of Na^+^ influx using an in situ perfused brain preparation that allowed uptake to continue for 10 min from solutions containing 140 mM Na^+^. Despite the previous results described above showing domination of the unsaturable component at 140 mM Na^+^, using this later technique they found about 25% of the uptake was via a saturable mechanism that could be inhibited completely by 25 µM dimethylamiloride, an amiloride derivative selective for Na^+^/H^+^ exchangers such as NHE1 or NHE2. These NHEs are now known to be expressed in brain endothelial cells (see Sect. 4.4.2). Why this component could be seen in these experiments but not the earlier experiments may be explained by the possibility that the perfused brains were acidotic. It is known from in vitro experiments with brain endothelial cells [268] (see Sect. 4.5.2) that NHE activity is strongly activated at low intracellular pH. Ennis et al. [267] found that neither bumetanide, an inhibitor of Na^+^, K^+^, Cl^−^ cotransporters, at the high concentration of 250 µM nor hydrochlorothiazide, an inhibitor of Na^+^, Cl^−^ cotransporters, at the high concentration of 1.5 mM had any effect on tracer Na^+^ influx into the brain. This bumetanide result is consistent with an earlier result from Smith and Rapoport [269] that the rate of ^22^Na^+^ tracer entry into the brain across the blood–brain barrier was the same at 25 and 95 mM Cl^−^.

#### Transcellular versus paracellular routes for Na^+^ and Cl^−^

It has been possible to inhibit K^+^ tracer fluxes but not those for Na^+^ across the blood–brain barrier in vivo using ouabain. Why? A possible explanation is suggested by the properties of the tracer influx of Na^+^ at normal concentrations: (i) it is not blocked by inhibitors of the Na^+^ transporters known to be present and is not affected by [Cl^−^]_plasma_ (see Sect. 4.3.3); (ii) it is unsaturable and (iii) it is much larger than any possible net flux of Na^+^ across the barrier (see Sect. 4.3.5). These are the properties expected for transport by a paracellular route. Such transport: (i) would be independent of any of the transporters involved in the transcellular route and thus not subject to their inhibition, (ii) would be similar to electrodiffusion (diffusion of ions when there are both concentration and electrical potential gradients) which is unsaturable, and (iii) would have a net flux much smaller than influx or efflux because [Na^+^] is almost the same on both sides of the barrier and the potential difference across it is small, e.g. 4 mV, ISF relative to plasma (see Sect. 6.4 and associated footnotes). For further arguments in favour of a paracellular route for transport see [270].

Electrodiffusion by a paracellular route would of course imply that there is a paracellular conductance. The conductance of this proposed pathway must not be greater than the total measured conductance of the barrier. Smith and Rapoport [261] calculated that the Na^+^ and Cl^−^ permeabilities they measured, if occurring by electrodiffusion, would together correspond to an electrical conductance of about 1.6 × 10^−4^ s cm^−2^ (or resistance of 8000 $\Omega$ cm^2^). This is less than the best available measurements of barrier conductance, 5–7 × 10^−4^ S cm^−2^ [271, 272].

The tight junctions at the blood–brain barrier are among the tightest in the body. This probably reflects the high abundance of claudin-5, which is known to reduce the conductance of tight junctions to low levels. However, apparently there are no estimates, other than from the transendothelial resistance estimates above, of just how low the conductance becomes. For reviews of the properties of the tight junctions see [273–276].

There are two further observations in the literature that argue against all of the observed tracer fluxes of Na^+^ and Cl^−^ across the blood–brain barrier being via a paracellular route. The first concerns fluxes of Na^+^. In the perfused brain experiments discussed in Sect. 4.3.3 about a third of the unsaturable Na^+^ influx was inhibited by phenamil, an amiloride derivative [267] thought to be selective for inhibition of epithelial Na^+^ channels, which may mean that less than half of the Na^+^ tracer influx is paracellular. However because in these experiments the inhibitor was used at a much higher concentration, 25 µM, than the sub-micromolar concentrations found to block Na^+^ channels in epithelia [277, 278], it is conceivable that its effect was not on a channel in the plasma membrane but on some other target. One possibility is that it was acting less specifically to reduce the conductance of the tight junctions and thus affect movement via a paracellular route. Amiloride itself, at concentrations higher than those used to block Na^+^ selective channels, is known to reduce fluxes through tight junctions (albeit those with much higher conductances) [180, 279–281] lending support to the idea that phenamil might be doing something similar (compare the discussion of the effects of amiloride in choroid plexus in Sect. 3.6.1.1). Furthermore such inhibition may not be a total block of the permeation pathways, but may instead be a modification of the permeation pathway, for instance by changing surface charge [282–284]. That type of action may explain why the reported inhibition has been only partial. In summary, this evidence may be consistent with anything up to almost all of the Na^+^ tracer influx being paracellular.

**Fig. 10:**
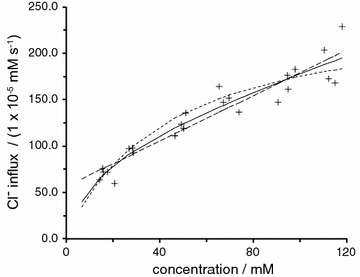
Unidirectional Cl^−^ influx into parietal cortex as a function of [Cl^−^]_plasma_. The Cl^−^ influx has been calculated as the transfer constant, *k*, taken from Fig. 3a in [269] times [Cl^−^]_plasma_. The short-dashed curve is plotted using the expression for transport by a saturable transporter with *k* = *V*_*max*_/(*K*_*m*_ + [Cl^−^]_plasma_), maximum transport rate, *V*_*max*_ = 250 mM s^−1^, and Michaelis constant, *K*_*m*_ = 43 mM as described by Smith and Rapoport [269]. The *solid curve* is the best fit for a model with a single saturable component plus an unsaturable component. 53% of the influx is unsaturable at [Cl^−^]_plasma_ = 118 mM. As shown by the *long-dashed line*, it is even possible to fit the data more closely than by Smith and Rapoport’s expression by assuming a high affinity, saturable component and an unsaturable component with 72% of the uptake unsaturable at 118 mM. The fitting is described in more detail in footnote 14

The second of the observations that can be used to argue against a paracellular route concerns fluxes of Cl^−^. Tracer influx of Cl^−^ was observed to occur at a rate comparable to that for Na^+^ [261] but, in contrast to Na^+^ transport, that for Cl^−^ was reported to be entirely saturable as if it were occurring via transporters and hence transcellular (see Fig. 12). However in interpreting their results, Smith and Rapoport [269] assumed that all of the transport occurred by a single mechanism, either saturable or unsaturable. If it is assumed instead that transport has two components, one saturable and the other unsaturable, the improvement in the fit to their data is statistically significant (see Fig. 10 and 14) and most of the transport is found to be unsaturable. Thus their data are consistent with a large proportion of the tracer flux of Cl^−^ occurring via an unsaturable and hence possibly paracellular route.

In conclusion, evidence that the tracer flux of Na^+^ occurs by a route that does not involve either the Na^+^-pump or NKCC1 is very strong but the suggestion that this separate route is paracellular still needs more direct confirmation.

To return to the initial question, there is a simple reason that may explain why it has been possible to demonstrate inhibition of K^+^ fluxes but not Na^+^ fluxes using ouabain. The principal mechanisms that load K^+^ into the endothelial cells, the Na^+^-pump and NKCC1, have ratios for Na^+^ and K^+^ transport of 3/2 and 1/1 (see Sects. 4.6.1 and 4.6.4). By contrast the ratio for a paracellular, unsaturable, electrodiffusion-like route would be expected to be roughly the same as the ratio of the Na^+^ and K^+^ concentrations, i.e. approximately 150/4 = ~37. Thus on this basis alone it is plausible that transcellular transport will be a much larger fraction of the total transport of K^+^ than it is of Na^+^.

#### Comparison of Na^+^ tracer flux, net Na^+^ flux inferred from tracer data and the net Na^+^ flux needed to support any significant fluid secretion

It is instructive to compare measured Na^+^ tracer fluxes [16, 261] (see Sect. 4.3.2) with the net Na^+^ flux that would occur if tracer flux represented paracellular electrodiffusion and with the net Na^+^ flux that would occur as part of fluid secretion, e.g. for purposes of illustration at 200 ml day^−1^ for an adult human. Calculation of net flux via electrodiffusion driven by a potential difference of 4 mV is explained in.^15^ The net flux as part of fluid secretion is calculated as the secretion rate multiplied by the concentration of Na^+^ in the secretion. These values are compared in Table 2.

**Table 2:** Comparison of observed Na^+^ tracer influx, calculated net Na^+^ flux driven by 4 mV if by electrodiffusion and Na^+^ flux needed for secretion of 200 ml day^−1^ at the blood–brain barrier

Quantity and formulae	Value (mmol kg^−1^ h^−1^)
$$ \begin{aligned} {\text{Tracer influx}} &= PS \times \left[ {{\text{Na}}^{ + } } \right] \\ &= 70\, {\text{cm}}^{ 3} \, {\text{kg}}^{ - 1} {\text{h}}^{ - 1} \times 0.15 \, {\text{mmol}} \, {\text{cm}}^{ - 3} \end{aligned} $$	10.5
$$ \begin{aligned} {\text{If }}\Delta V &= 4 {\text{mV}},{\text{ net flux}} \\ &= {\text{tracer influx}} \times \Delta V \times \left( {F/RT} \right) \\ & = 10.5\, {\text{mmol}}\, {\text{kg}}^{ - 1} {\text{h}}^{ - 1} \times 4\,{\text{mV}}/ 27 \, {\text{mV}} \end{aligned} $$	1.55
$$ \begin{aligned} {\text{Net flux of Na}}^{ + }& {\text{needed for secretion of }} \; 200 \, {\text{ml}}\, {\text{day}}^{ - 1} {\text{in a 1}}. 4 \, {\text{kg brain}} \\ \qquad &= 0.2 \, {\text{l}} \, {\text{day}}^{ - 1} \times 150 \, {\text{mmol}}\, {\text{l}}^{ - 1} /( 24 \, {\text{h}} \, {\text{day}}^{ - 1 } \times 1.4 \, {\text{kg}}) \end{aligned} $$	0.9

The values in the table illustrate two points. The first and most important point is that tracer influx is much larger than all credible net fluxes calculated from the possible rates of fluid secretion. As noted by Davson and Segal [173] because tracer influx is so much larger than any conceivable net flux, it must to a large extent be balanced by a tracer efflux. The second point is that, if tracer flux occurs by electrodiffusion, then even quite small potential differences across the barrier between ISF and plasma will produce net fluxes comparable in size to those that might support secretion. As discussed in Sect. 6.4 and associated footnote, it is likely that there is a potential, of the order of 3 or 4 mV [285], ISF positive, across the blood–brain barrier. The Na^+^ flux driven by this small potential would be in the opposite direction to that required for secretion, but Cl^−^ flux would be in the same direction as the secretion. This could have important implications as the potential difference may become more positive in acidosis. However, there does not appear to have been any study on the consequences of changes in this potential for net transport of Na^+^, Cl^−^ and fluid across the blood–brain barrier (but see [286]).

To restate the main conclusion from these comparisons, net flux by a mechanism involving active transport and hence presumably transcellular can make a major contribution to total net flux across the blood–brain barrier but at the same time be much smaller than tracer influx (or efflux) measured across the barrier. Inhibition of transport via an active, hence transcellular mechanism may thus not be detectable by measuring tracer influx because the latter occurs primarily by a separate, passive mechanism, presumably paracellular (see Sect. 4.3.4 but also the discussion of results for dimethylamiloride [267] in Sect. 4.3.3).

#### Results concerned with water transfer

As can be judged from the magnitude of unidirectional fluxes of water noted earlier (see Sect. 2.1), water molecules can easily cross the blood–brain barrier. However, because permeability = flux/concentration and the concentration of water is so high (55 M), permeability to water could still be relatively small despite these large observed unidirectional fluxes. It is thus necessary to ask if the barrier is sufficiently permeable to water that the water component of fluid secretion could be driven by the small osmotic gradients that can exist. Osmotic water permeability (or in different units the filtration constant) has been measured for the blood–brain barrier in rabbits [287, 288] and humans [289] and in both is close to 1.1 × 10^−3^ cm s^−1^ (or 1.2 × 10^−6^ ml min^−1^ cm^−2^ mM^−1^). (See 16 for definitions of some of the constants used to state the permeabilities and their units and for the calculations that are the basis of the comparisons made here). This value, which is consistent with there being large unidirectional fluxes, can be used to calculate the osmotic gradient that would be needed for fluid secretion of 0.1 µl g^−1^ min^−1^ (corresponding to 200 ml day^−1^ in a human). This water flow could be driven by as little as 0.4 mM NaCl concentration difference. As this value is so small, there is no need to propose anything beyond osmotically driven water flux to explain the net flux of water across the blood–brain barrier.

The measured water permeability is well within the possible range of water permeabilities for protein-free lipid bilayers [290]. Thus it is not surprising that no aquaporins have been detected in brain endothelial cells in vivo [13] and their appearance in these cells in culture is thought to be a result of dedifferentiation [291] and not an indication of the normal situation within the brain.

Even though the permeability of the lipid bilayers may be adequate, that of the endothelial cell membranes may be increased further by the presence of transporters and other proteins. In particular GLUT1 is known to be highly expressed in the brain endothelium (see Sect. 2.4.1) and it is known to increase the osmotic water permeability of membranes in other cell types [154–157].^17^

### Evidence for the presence of ion transporters able to move osmotically important solutes across the blood–brain barrier

**Fig. 11:**
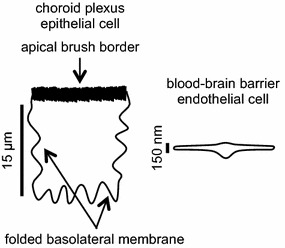
Schematic diagram emphasizing differences between choroid plexus epithelial and blood–brain barrier endothelial cells relevant to the detection of transporters. Note the difference in *scale bars*

Identification and localization of the transporters involved in the transport of Na^+^, K^+^, Cl^−^ and HCO_3_^−^ across the endothelial cells of the blood–brain barrier is challenging and has not reached the same level of certainty as at the choroid plexus. This is partly because brain endothelial cells are very different in shape from choroidal epithelial cells (see Fig. 11). Each choroid plexus epithelial cell has an apical membrane that is tightly folded into a brush border and a basolateral membrane that is less tightly but still extensively folded. Furthermore the apical and basolateral regions of the cells are sufficiently far apart to be distinguishable by light microscopy (see Fig. 5). So, when a choroid plexus cell is viewed in section, transporters can be clearly detected at the cell borders both because there is a lot of membrane folded into the border and because there is a high density of transporters in the membranes.

**Fig. 12:**
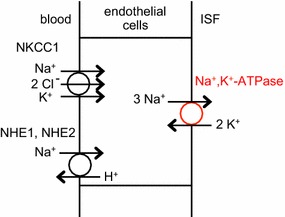
Transporters localized to membranes of the endothelial cells of the blood–brain barrier. The Na^+^, K^+^-ATPase and the Na^+^/H^+^-exchangers, NHE1 and NHE2, are also present on the opposite sides of the cell but at lower densities

By contrast it is difficult to detect transporters that may be present on endothelial cells of the blood–brain barrier. This is because there are few foldings to increase the amount of membrane at the two surfaces of the cells and the numbers of ion transporters per unit area of membrane may be relatively small (as judged by measured ion fluxes). Furthermore, because the endothelial cells are so thin, under the light microscope it is not possible to distinguish between luminal and abluminal membranes. Despite these difficulties, as discussed in Sects. 4.4.1 and 4.4.2, four ion transporters have been identified and localized primarily to one membrane or the other. These are the Na^+^, K^+^-ATPase, a.k.a. the Na^+^-pump, NKCC1, NHE1 and NHE2 as shown in Fig. 12. There is also evidence for the presence and activity of AE2, NBCn1 and NBCe1. A large number of proteins including transporters have also been identified in brain endothelial cells at the transcript level [292, 293].

#### Expression and localization of Na^+^, K^+^-ATPase

The key transporter that couples metabolic energy to ion transport at the blood–brain barrier is the Na^+^-pump otherwise called Na^+^, K^+^-ATPase. The presence of this pump in the membranes of blood–brain barrier endothelial cells has been firmly established by evidence from a number of different studies. These studies have been variously based on: ouabain-sensitive release of phosphate from ATP by the Na^+^, K^+^-ATPase in isolated microvessels [294] and their isolated membranes [295, 296], inhibition by ouabain of K^+^ or Rb^+^ uptake into isolated microvessels [294, 297, 298] and cultured brain endothelial cells [299], ouabain binding [294, 300, 301] and the presence of ouabain-inhibited, K^+^-dependent p-nitrophenylphosphatase (K-NPPase) activity [302–304] in the abluminal membranes of the endothelial cells in vivo [295, 305–312].

Preferential localization of the Na^+^-pump to the abluminal rather than luminal membrane remains likely but controversial. Most but not all electron microscopy studies using the K-NPPase cytochemical assay have found primarily abluminal localization. This assay, however, has to be carefully controlled because it is clear that it can detect other ATPase activities including that of alkaline phosphatase, which is present in both membranes of the endothelial cells. In all studies using this assay where the necessary control criteria were met, i.e. activity not blocked by alkaline phosphatase inhibitors and either dependent on K^+^ or inhibited by ouabain, predominantly abluminal localization was observed [295, 305–309, 312] (see below). However, even in these studies, because the assays were conducted after tissue fixation there is the ever present risk that Na^+^-pumps were inactivated prior to the assay [304, 313, 314]. Furthermore, it is conceivable that fixation might have affected the pumps in one membrane more than the other [311]. It should be noted that, using the K-NPPase cytochemical assay, differences in localization of the Na^+^-pumps were observed in the choroid plexus that have never been adequately explained [315]. (For further discussion see 18).

An alternative method for determining the sidedness of pump activity is based on separation of luminal and abluminal membranes by density gradient centrifugation. Betz et al. [295] and Sanchez del Pino et al. [296] detected pump activity in separated membrane fractions using release of phosphate from ATP. Betz et al. found that ouabain-sensitive, Na^+^- and K^+^-dependent release by Na^+^, K^+^-ATPase was at much higher levels in that fraction identified as being primarily abluminal. Sanchez del Pino et al. using markers for luminal and abluminal membranes found that 75% of Na^+^, K^+^-ATPase activity was abluminal and 25% luminal. Furthermore they found that the concentrations of ouabain required for inhibition of activities in the luminal and abluminal membranes differed suggesting that there may be different isoforms expressed on the two surfaces. Three different α subunits and two β subunits of the Na^+^, K^+^-ATPase have been reported to be expressed at the blood–brain barrier allowing for the possibility of six different pumps ([316], but see [317]).

It is interesting to note that in a more recent study of fractionated membranes prepared from porcine brain capillaries and analysed for Na^+^, K^+^-ATPase not by its activity but on the basis of its protein sequence (selected/multiple reaction monitoring experiments, SRM/MRM, see e.g. [318]), it was far from clear that Na^+^, K^+^-ATPase expression was predominantly at the abluminal surface [98]. Given these conflicting results the exact sidedness of the Na^+^-pump remains an unresolved issue. At present the balance of evidence supports a predominant localization of the Na^+^-pumps (at least functionally) in the abluminal membrane (see Sect. 4.3.1 for further discussion). It is important that the distribution of activity be determined in future work since it provides clues as to the direction of movement of Na^+^ at the blood–brain barrier.

#### Evidence for expression of other ion transporters at the blood–brain barrier

NKCC1 has been localized primarily to the luminal membrane by O’Donnell and coworkers using bumetanide binding assays with cultured endothelial cells [319] and immuno-electron microscopy with brain slices [237, 320]. They also used immuno-electron microscopy to localize NHE1 and NHE2 [321] primarily but not exclusively on the luminal membrane. NHE1 and NHE2 have been detected by western blot analysis in cultured rat cerebral microvascular endothelial cells [322], cultured bovine microvascular endothelial cells [321] and freshly isolated rat cerebral microvessels [321]. Other results regarding NKCC1, NHE1 and NHE2 expression have been reviewed by O’Donnell [19].

**Fig. 13:**
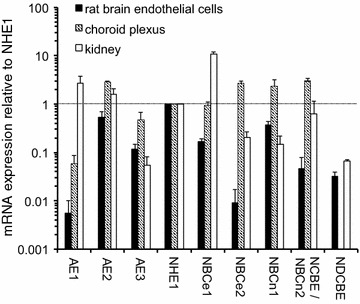
mRNA expression relative to that of the Na^+^/H^+^-exchanger, NHE1, in rat brain endothelial cells, choroid plexus and kidney cortex. At the blood–brain barrier expression of mRNAs for anion exchangers 2 and 3, AE2 and AE3, the Na^+^/H^+^-exchanger, NHE1, the charge-transporting Na^+^, HCO_3_^−^-cotransporter, NBCe1 and the neutral Na^+^, HCO_3_^−^-cotransporter, NBCn1, are prominent. Those for the Cl^−^-dependent Na^+^, HCO_3_^−^-cotransporters, NCBE/NBCn2, and NDCBE are clearly detected. Redrawn from data in [336]

HCO_3_^−^ transporters have been detected at the mRNA level in brain endothelial cells (see Fig. 13) and at the protein level by fluorescence microscopy on microvessels in brain slices. The relative levels of mRNA compared with the ubiquitous exchanger NHE1 have been measured in brain endothelial cells and compared with those in isolated choroid plexus and renal cortex. Prominent expression of mRNA for the Cl^−^/HCO_3_^−^ exchanger AE2 and the Na^+^, HCO_3_^−^ cotransporters NBCe1 and NBCn1 were detected in brain endothelial cells with lower levels for AE3, NCBE/NBCn2 and NDCBE. NBCn1 was detected in membranes isolated from cultured rat brain endothelial cells by western blot analysis [322]. Immunohistochemistry on relatively thick frozen rat cortical brain slices has shown clear selective labelling of microvessels for AE2 and labelling for NBCe1 and NBCn1 [241]. A preliminary report indicates that the same transporters are present in both bovine cerebral microvascular endothelial cells (CMEC) and freshly isolated rat brain microvessels [323, and M.E. O’Donnell personal communication]. So far localization of these HCO_3_^−^ transporters to one or the other of the brain endothelial cell surfaces has not been achieved.

Detection and location of K^+^ channels that may be important in blood–brain barrier function is considered in Sect. 4.5.3 alongside the functional evidence for the currents they mediate.

### Functional evidence of ion transport at the blood–brain barrier from in vitro studies with brain endothelial cells

The in vivo tracer studies described above demonstrate that small monovalent ions can cross the blood–brain barrier. However, a major hindrance to in vivo studies has been the lack of methods for direct measurements of net fluxes across the barrier and tracer fluxes into or out of the endothelial cells. In vitro systems, i.e. isolated brain microvessels and primary cultures of brain microvascular endothelial cells, allow for better access to the brain endothelial cells under conditions that can be more closely controlled. It is thus possible to measure fluxes into and out of the endothelial cells. Unfortunately at present there is no in vitro preparation that allows determination of net fluxes of Na^+^, K^+^, Cl^−^ or HCO_3_^−^ across the cells [10].

#### Evidence concerning Na^+^, K^+^ and Cl^−^ transport

Evidence obtained from early studies on the presence and functions of the Na^+^-pump in brain endothelial cells in vitro has already been mentioned (see Sect. 4.4.1). Evidence of other transporters for Na^+^ has also been obtained. Betz used both isolated microvessels and cultured endothelial cells [324] to demonstrate the presence of amiloride-sensitive, saturable processes for ^22^Na^+^ entry into the cells. This entry was stimulated by increasing internal Na^+^ (preincubation in the presence of ouabain) or by increased internal H^+^. It was inhibited by extracellular Na^+^, H^+^, Li^+^ and NH_4_^+^ strongly suggesting the presence of a Na^+^/H^+^ exchanger presumably located in the abluminal membrane, this being the side of the endothelial cells exposed to the bathing solution. However, they could not detect any process that could be inhibited by furosemide which impairs Na^+^, Cl^−^ cotransport including that by NKCC1 (discussed below). In hindsight this is surprising as other groups have seen activity of NKCC1. An unfortunate choice of experimental conditions may have hidden the function of this transporter. In the study by Betz, uptakes were usually determined using buffer with very low Na^+^, K^+^ and Cl^−^ concentrations and it is not clear that the effects of furosemide were ever investigated in the presence of ouabain (to inhibit the large K^+^ influx and Na^+^ efflux via the Na^+^-pump) with simultaneously sufficient Na^+^, K^+^ and Cl^−^ to produce influx of Na^+^ and K^+^ by NKCC1. Nor were the effects of furosemide investigated in the presence of both ouabain and amiloride.

The presence of Na^+^, K^+^, Cl^−^-cotransport in isolated rat brain microvessels was subsequently suggested by the finding of an ouabain-insensitive, Na^+^- and Cl^−^-dependent component of Rb^+^ influx [325]. The cotransport was also seen in cultured bovine brain microvascular endothelial cells by O’Donnell’s group in 1993 [326] and the presence of this activity was soon confirmed by the results of others [327, 328]. It was shown that ouabain, blocking Na^+^, K^+^-ATPase, and bumetanide, blocking Na^+^, K^+^, 2Cl^−^-cotransport, inhibited K^+^ influx into the cultured cells to roughly equal extents and the combination inhibited entry by about 90% [299, 329]. The ouabain-insensitive transport was inhibited almost equally by bumetanide or by omission of either Cl^−^ or Na^+^ from the bathing solution [329]. With application on just one side of endothelial cells grown on permeable supports, the inhibition of K^+^ influx into the cells was much greater when bumetanide was applied on the luminal rather than abluminal side. From their observations, Sun et al. inferred that about 90% of the cotransporter responsible was present on the luminal side [319]. The molecular identity of the cotransporter as NKCC1 was established soon after [330]. These results in combination with the immunohistochemistry in brain slices localizing NKCC1 to the luminal membrane of the endothelial cells strongly suggest that NKCC1 plays an important role at the blood–brain barrier.

Unfortunately O’Donnell and colleagues [319, 329] did not test the sidedness of inhibition by ouabain. It would be very interesting to know the proportions of K^+^ entry inhibited on each side of the cells given that evidence from both expression studies (Sect. 4.4.1) and in situ brain perfusion (Sect. 4.3.1) now suggests that Na^+^-pumps are present in both membranes.

Though there is substantial information available about transport of Na^+^ and K^+^, much less is available about Cl^−^. It is very likely that in addition to NKCC1 there are channels conducting Cl^−^ that are important for the functions of the blood–brain barrier. It is clear from patch-clamp studies that anion conductances exist (see e.g. [331, 332] which will mediate efflux of Cl^−^ from the cells, but no channels have been identified at the molecular level. Cl^−^ is also inextricably involved in the transport of HCO_3_^−^ by AE2.

#### Evidence concerning HCO_3_^−^, Cl^−^ and H^+^ transport

**Fig. 14:**
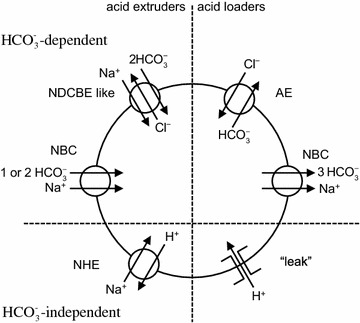
Types of transporters that load or extrude acid from brain endothelial cells. In the presence of CO_2_/HCO_3_^−^ acid is added (i.e. HCO_3_^−^ is removed) primarily by either Cl^−^/HCO_3_^−^ exchange (AE: AE2 and possibly AE3) or a Na^+^, HCO_3_^−^ cotransporter operating in a 3:1 mode (NBC: probably NBCe1). Acid is extruded by Na^+^ driven transport by several Na^+^, HCO_3_^−^ cotransporters either Cl^−^-independent (NBC: e.g. NBCn1 (*n* = 1) and NBCe1 in a 2:1 mode (*n* = 2) or Cl^−^-dependent (NDCBE-like). In the absence of CO_2_/HCO_3_^−^ the principal loader is here called the “leak” and the principal extruder is a Na^+^/H^+^-exchanger (NHE). The classification into loaders and extruders follows that used in [399, 545]

It is very difficult to study the transport of HCO_3_^−^ across the blood–brain barrier either in vivo or in vitro using radiotracers because there is interconversion between HCO_3_^−^ and CO_2_ (see Sects. 6.1 and 6.4.2). However, transport of H^+^ and HCO_3_^−^ into and out of brain endothelial cells can be studied in vitro by monitoring the effects that movements of these ions have on intracellular pH (pH_i_). This has been done with brain endothelial cells grown in culture using the fluorescent indicator BCECF. In the steady-state in vivo, the rate of transport of HCO_3_^−^ out of cells plus the rate of transport of H^+^ into cells, together called acid loading, must be almost the same as acid extrusion because otherwise pH_i_ could not be stable given that H^+^ and HCO_3_^−^ are the major ions affecting intracellular pH.^19^ The types of transport thought to be important in movements of H^+^ and HCO_3_^−^ and their classification into acid extruders and loaders and the dependence of the various transporters on presence of HCO_3_^−^ are indicated in Fig. 14.

The transporters indicated below the horizontal line in Fig. 14 are functional whether or not HCO_3_^−^ is present. The first, i.e. the “leak” is detectable in the presence or absence of HCO_3_^−^ and is seen as a slow acidification of the cells in the absence of HCO_3_^−^ or presence of DIDS, which blocks many forms of HCO_3_^−^ transport, combined with the absence of Na^+^ (replacement with NMDG^+^) or presence of EIPA, which blocks NHEs. This acidification is not blocked by replacement of Cl^−^ by gluconate^−^. It is, however, apparently reversed by replacement of external Na^+^ by K^+^, which is expected to strongly depolarize the cells [268]. This suggests that the “leak” is sensitive to membrane potential and independent of HCO_3_^−^ and thus plausibly a channel-like permeability to H^+^. The “leak” acts as an acid loader but at a much smaller rate than the acid loading described below in the presence of HCO_3_^−^ and Cl^−^.

The other type of HCO_3_^−^-independent transporter shown in Fig. 14 is NHE. Na^+^/H^+^ exchange has been found in many studies looking at recovery of cells from markedly reduced pH_i_ after additions of acid to the cell interior [268, 324, 333–336]. However, if rat brain endothelial cells sit in nominally HCO_3_^−^-free Hepes buffered solution, remarkably little happens to their pH_i_ when Na^+^/H^+^ exchange is inhibited either by exposure to EIPA, an inhibitor of Na^+^/H^+^ exchangers, or even more telling by replacement of all external Na^+^ with the membrane impermeant cation NMDG^+^ [336]. This suggests that under these resting conditions Na^+^/H^+^ exchange occurs at only a very slow rate, comparable to the “leak”, because if it were rapid, pH_i_ would decrease after the exchanger was either silenced by EIPA or reversed by removal of external Na^+^. This conclusion has been confirmed by studying the rate of Na^+^/H^+^ exchange as a function of pH_i_, which reveals that exchange is markedly activated by low pH_i_ but is almost quiescent at resting pH_i_ [268].

**Fig. 15:**
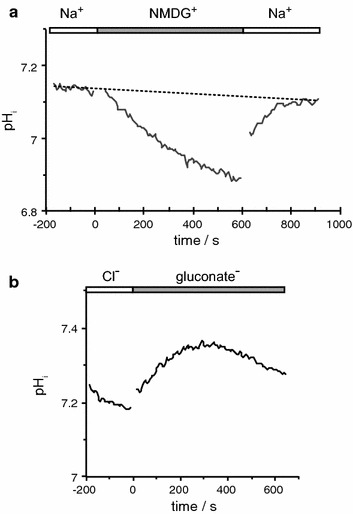
Effects of Na^+^ or Cl^−^ removal on pH_i_ in the presence of CO_2_/HCO_3_^−^. **a** When NaCl is replaced by *n*-methyl-d-glucosamine chloride, NMDG Cl, there is a progressive acidification of cells that is initially somewhat faster. Replacement of the Na^+^ allows pH_i_ to recover. The *dashed line* indicates the drift in pH_i_ observed when no substitution is made. It is attributed to the “leak” described in the text. This trace is the mean of four experiments. **b** When NaCl is replaced by Na gluconate there is a transient rapid alkalinization of the cells. This trace is from a single experiment. Both **a** and **b** are replotted from data sets used in [336]. Both effects are statistically significant, see Table 3 in [336]

**Fig. 16:**
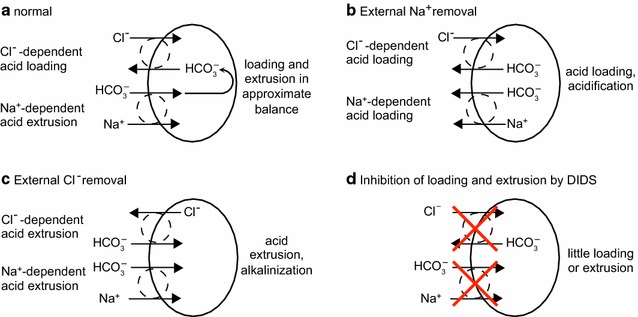
Simplified scheme for explaining the initial results of ion substitutions and inhibition by 4,4′-diisothiocyano-2,2′-stilbenedisulfonic acid (DIDS). In **a** the rates of acid loading by Cl^−^/HCO_3_^−^ exchange and acid extrusion by Na^+^, HCO_3_^−^-cotransport are nearly in balance and the pH is stable. In **b** removal of external Na^+^ reverses the direction of the Na^+^ gradient and Na^+^, HCO_3_^−^-cotransport is acid loading until the internal Na^+^ is depleted. While both types of transport are acid loading, pH_i_ falls, i.e. there is cellular acidification. In **c** removal of external Cl^−^ reverses the direction of the Cl^−^ gradient and Cl^−^/HCO_3_^−^ exchange is acid extruding until the internal Cl^−^ is depleted. While both types of transport are acid extruding, pH_i_ increases, i.e. there is cellular alkalinization. In **d** DIDS blocks both types of transport and there is little acid loading or extrusion and only slow if any change in pH_i_

The transporters indicated above the horizontal line in Fig. 14 are expected to be active whenever HCO_3_^−^ is present. Their activities have been revealed primarily by looking at the effects of ion substitutions and transport inhibitors on the rate of change of pH_i_. With cells initially in a solution containing Na^+^, Cl^−^ and HCO_3_^−^, Na^+^ removal from the external solution led to a marked increase in the rate of pH_i_ decrease, i.e. it led to a marked acidification, see Fig. 15a. This effect together with the rate of pH increase produced by removing Cl^−^ (shown in Fig. 15b) and with the block of both of these effects by preincubation with DIDS can be interpreted as existence for two types of activity: Na^+^-driven, HCO_3_^−^-dependent acid extrusion and Na^+^-independent, Cl^−^- and HCO_3_^−^-dependent acid loading (see Fig. 16). In this scheme, acid loading (i.e. HCO_3_^−^ extrusion) and acid extrusion (HCO_3_^−^ loading) are both occurring when the cells are unchallenged but are in approximate balance (see Fig. 16a). Replacing external Na^+^ with membrane impermeant NMDG^+^ reverses the direction of the Na^+^, HCO_3_^−^ cotransport so that it contributes to loss of HCO_3_^−^ from the cells until they are depleted of Na^+^ (Fig. 16b). Thus initially the rate of acidification is changed from that corresponding to approximate balance of loading and extrusion, to that seen when the major type of extruder is converted into a loader [336].

The Cl^−^ dependence of Na^+^, HCO_3_^−^ cotransport can be investigated by replacing Cl^−^ with membrane impermeant gluconate^−^, allowing time for intracellular Cl^−^ to be depleted and then replacing Na^+^ with NMDG^+^. In experiments where this was done, the change observed in acidification rate induced by removal of external Na^+^ appeared to be less after depleting Cl^−^, but the effect did not reach statistical significance [336]. Na^+^, HCO_3_^−^ cotransport also contributes to recovery of pH_i_ when a cell is acidified from pH_i_ = 7.1–6.5 using the NH_4_^+^-pulse technique. When the effect of Cl^−^ was tested in this type of experiment the HCO_3_^−^-dependent recovery was approximately twice as fast in the presence of Cl^−^ than when Cl^−^ had been replaced by gluconate^−^. This finding suggests that part of the Na^+^, HCO_3_^−^-cotransport is Cl^−^-dependent, i.e. occurring by a NDCBE-like cotransporter [268]. There appear to be three Na^+^, HCO_3_^−^-cotransporters involved in HCO_3_^−^ transport in brain endothelial cells, NBCe1 and NBCn1, which are Cl^−^-independent and the NDCBE-like transporter, which is Cl^−^-dependent.

The functional data from cultured cells have revealed the existence of a net inward Na^+^-dependent flux of HCO_3_^−^ but not the size of the contribution to this flux made by each of the individual cotransporters. The measured net flux will be the sum of fluxes occurring by NBCn1 and by NDCBE-like transporter, but whether the flux via NBCe1 adds to or subtracts from this sum depends on its mode of operation. If NBCe1 transports 2 HCO_3_^−^ per Na^+^, the transport will be into the cell and it will add to the sum. However, if NBCe1 transports 3 HCO_3_^−^ per Na^+^, it will transport out of the cell and it will subtract from the sum.

Evidence for the functional presence of a Cl^−^/HCO_3_^−^ exchanger was obtained from observing the initial effect of removing external Cl^−^. Replacing external Cl^−^ with membrane-impermeant gluconate^−^ reverses the direction of Cl^−^/HCO_3_^−^ exchange so that it contributes to entry of HCO_3_^−^ into the cells until they become depleted of Cl^−^. Thus initially acidification rate is changed from that corresponding to approximate balance of loading and extrusion (Fig. 16a), to that seen when the major acid loader is converted into an extruder (Fig. 16c) [336].

The Na^+^ dependence of Cl^−^, HCO_3_^−^ exchange can be investigated by replacing Na^+^ with membrane-impermeant NMDG ^+^, allowing time for intracellular Na^+^ to become depleted and then replacing Cl^−^ with gluconate^−^. The change in alkalinization rate induced by removal of external Cl^−^ was observed to be almost the same before and after depleting brain endothelial cells of Na^+^ [336]. Thus the Cl^−^, HCO_3_^−^ exchanger appears to be Na^+^-independent, a property of the AE family of exchangers. The most prominently expressed member of this family in these cells is AE2.

The initial effects of ion substitutions on pH_i_ are adequately described by the scheme shown in Fig. 16. In the longer term, the effects of substitutions are likely to be more complex, e.g. after external Cl^−^ removal the cells should shrink markedly and there will also be changes in membrane potential and in concentrations of ions other than Cl^−^, HCO_3_^−^ and H^+^.

As stated above preincubation of brain endothelial cells with DIDS prevents changes in acidification and alkalinization produced by removal of external Na^+^ or Cl^−^. Yet acutely in the presence of Na^+^, Cl^−^, and HCO_3_^−^ adding DIDS produces no observable effect on pH_i_. This suggests that DIDS is blocking both the loaders and extruders as indicated in Fig. 16d [336].

It is possible to produce a rapid alkalinization of cells in CO_2_/HCO_3_^−^-containing solution either by removing CO_2_/HCO_3_^−^ (see above) or by adding a weak base such as trimethylamine. Trimethylamine diffuses into cells and combines with H^+^ forming trimethylammonium^+^. This weak base procedure has the advantage of allowing investigation of recovery of pH_i_ towards normal in the presence or absence of HCO_3_^−^ and Cl^−^. Using this method, it was observed that recovery, which is acid loading, was 3 times faster when HCO_3_^−^ was present than when it was absent but only if Cl^−^ was also present. This suggests that acid loading occurs primarily by Cl^−^-dependent extrusion of HCO_3_^−^. The increase in recovery rate was blocked by DIDS, which together with the dependence on Cl^−^ adds to the evidence favouring the presence of an AE-like transporter.

Evidence that any particular transporter expressed accounts for an activity observed can in principle be obtained using specific inhibitors, by genetic knockouts or by transient knock-down of expression. EIPA is selective for NHE transporters but does not distinguish between the isoforms while DIDS inhibits many transporters and thus can not be used for precise molecular identification. siRNA has been used successfully to reduce levels of NHE1 and of AE2 in a cell line derived from rat brain microvascular endothelial cells. As expected lower expression of NHE1 reduced pH_i_ recovery rate following addition of acid to the cells while lower expression of AE2 reduced the alkalinization rate when Cl^−^ was removed [241].

#### Evidence concerning K^+^ transport

In vitro evidence for influx of K^+^ into brain endothelial cells across the luminal side via NKCC1 was discussed in Sect. 4.5.1. While evidence was obtained that the ouabain-sensitive Na^+^-pump mediates K^+^ influx in vitro, no evidence was obtained showing that this entry was abluminal (see Sect. 4.5.1). However, there is in vivo evidence showing that K^+^ influx from brain into the cells across the abluminal membrane is via the pump (see Sect. 4.3.1). On each side of the endothelial cells the influx exceeds the possible net flux across the cells, and thus at each membrane there must be a mechanism allowing efflux of most of the K^+^ that enters [332, 337]. There are no reports of K^+^, Cl^−^ cotransporters being present or active at the blood–brain barrier but also no reports of a careful search. By contrast many K^+^ channels have been characterized in cultured brain microvascular endothelial cells using patch clamp experiments [240, 332, 338–348]. In acutely dissociated rat brain endothelial cells mRNA is present and there are functional signatures for channels containing Kv1.3, Kir2.1 and Kir2.2 [332]. There is also western blot, immunocytochemical and patch clamp evidence for the presence of KCa3.1 in bovine brain endothelial cells [240, 347, 349]. Studies of the role of this channel should be aided by the recent development of a selective blocker, TRAM-34 [240, 347, 348]. However, while it seems from the evidence that there are several types of K^+^ channels that may play important roles in K^+^ transport at the blood–brain barrier, many details are missing including the localization of the ion channels to one membrane or the other of the endothelium. A plausible suggestion [332, 350] is that different channels are expressed on the two sides as this arrangement would allow separate regulation of K^+^ efflux across each membrane to achieve the proper balance with influx mechanisms. The role of the blood–brain barrier in regulation of [K^+^] in ISF and CSF is considered in Sect. 5.

### Mechanisms for ion and water movements across the endothelial layer of the blood–brain barrier: a current description

**Fig. 17:**
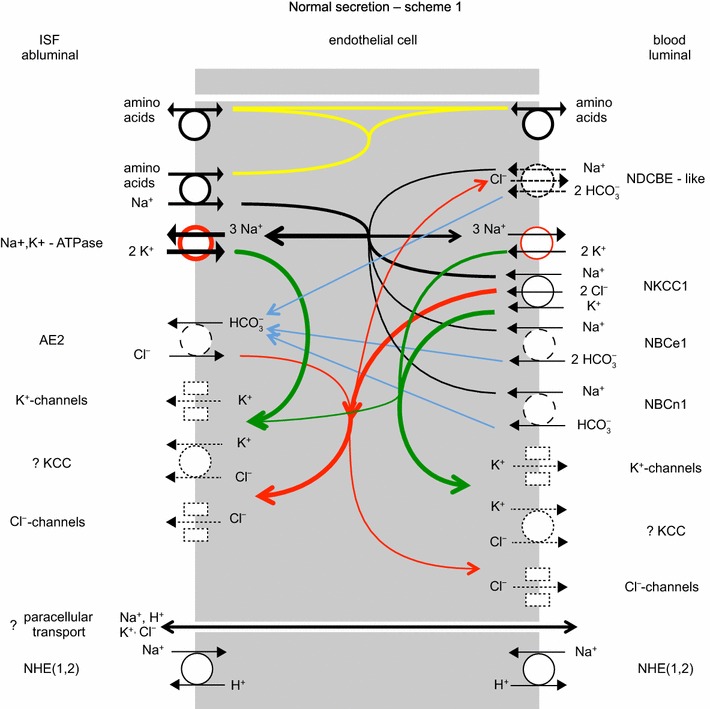
One possible scheme for ion transport by the blood–brain barrier. The Na^+^-pump is shown with more on the abluminal than luminal side of the endothelial cells. Transporters shown with *solid circles* have been identified in the membrane indicated; those with *dashed circles* have been identified at a molecular level but not localized; while those with *dotted circles* or rectangles have been identified only functionally. The *red circle* used in the symbol for the Na^+^, K^+^-ATPase indicates that energy for the transport is input from hydrolysis of ATP. *Arrows* within the cell indicate transfers: in *black* Na^+^, in *green* K^+^, in *red* Cl^−^, and in *blue* HCO_3_^−^. The electrical potential and ion concentrations inside the cells in vivo are not known. In primary cell culture the potential is about −40 mV [see e.g. 332]. Note that, in contrast to the choroid plexuses, at the blood–brain barrier there are likely to be conductances in both the luminal and abluminal membranes. NHE(1,2) is shown separately from the rest as it is unlikely to be active when pH_i_ is in the normal range. However, it is strongly activated by low pH_i_ as may occur in hypoxia/ischemia

**Fig. 18:**
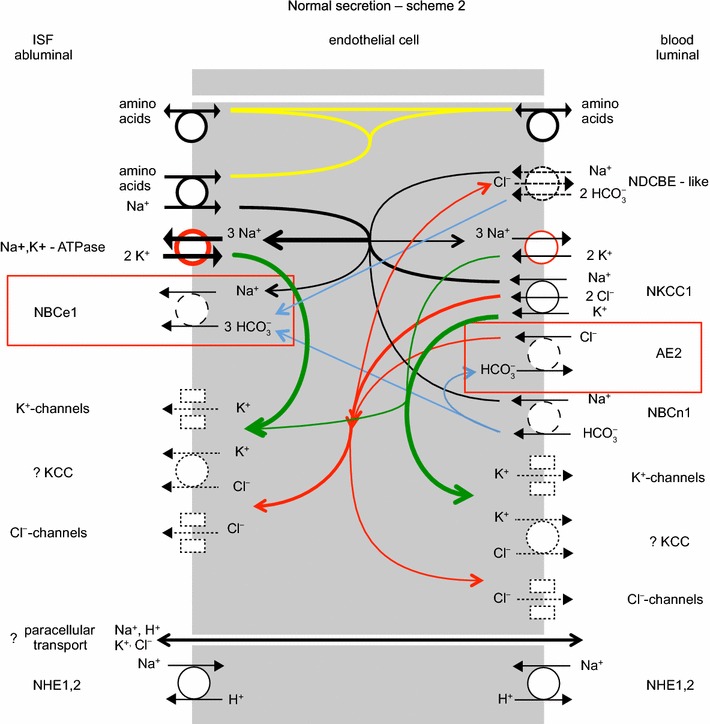
A second possible scheme for ion transport by the blood–brain barrier. This differs from the first, shown in Fig. 17, by swapping the positions of NBCe1 and AE2. For the key to lines and colours see the legend to Fig. 17. The consequences of the swap are considered in Sects. 4.6.1, 4.6.2

A number of possible schemes for transport across the blood–brain barrier based on both the in vivo and in vitro results above can be envisaged of which two are shown in Figs. 17 and 18. The two schemes differ in the suggested locations of AE2 and NBCe1. Experimental localization has not yet been reported. It should be noted that these are in some sense extremes of a range. Both or either of AE2 and NBCe1 may be present in both membranes.

Based on the schemes in the figure the net transport of ions appears to be as described in the following sections.

#### Na^+^ transport

The Na^+^, K^+^-ATPase or Na^+^-pump actively transports Na^+^ out of the cells into the ISF and to some extent to plasma. This reduces the intracellular [Na^+^] which provides the gradient for Na^+^ influx via other transporters. Na^+^ entry from the blood is thought to occur primarily by the Na^+^, K^+^, Cl^−^ cotransporter, NKCC1, though some must also occur by cotransport with HCO_3_^−^, probably via NBCn1 and possibly, if it is expressed in the luminal membrane, by NBCe1 working in the 1 Na^+^, 2 HCO_3_^−^ mode. If NBCe1 is instead in the abluminal membrane it would be sensible for it to be operating in the 1 Na^+^, 3 HCO_3_^−^ mode, which would mediate efflux of Na^+^ and HCO_3_^−^ from the cells to the ISF. From the concentrations in ISF, overall approximately 126/150ths of the net flux of Na^+^ across the endothelial cells will be accompanied by Cl^−^ (126 mM NaCl) while most of the remaining 24/150ths by HCO_3_^−^ (24 mM NaHCO_3_).

The principal routes for Na^+^ entry across the abluminal membrane are likely to be the Na^+^-linked transporters of organic solutes including prominently those for amino acids (see Sect. 2.4.2). If as appears likely, the preponderance of the linked transporters are in the abluminal membrane [120] and if in addition the Na^+^ flux via these is the largest component of Na^+^ entry to the cells (see Sect. 4.1.1 for comparison of the coupled Na^+^ fluxes and those needed for any realistic rate of secretion) then if the Na^+^-pumps were evenly distributed or primarily in the luminal membrane there would be an associated net active flux of Na^+^ towards plasma, i.e. a net reabsorption of fluid. At present the balance of evidence supports secretion under normal conditions (see Sect. 4.1). Obviously, it would be very interesting indeed if it were found that the distribution of Na^+^-pump activity could be altered.

#### HCO_3_^−^ and Cl^−^ transport

HCO_3_^−^ will enter the cells from the blood via NBCn1, which is known to transport 1 Na^+^ for 1 HCO_3_^−^, and possibly via NBCe1 transporting 1 Na^+^ and 2 HCO_3_^−^. HCO_3_^−^ will leave the cells via AE2 in the first scheme shown in Fig. 17 or via NBCe1 transporting 1 Na^+^ and 3 HCO_3_^−^ in the second scheme shown in Fig. 18. NBCn1 and the NDCBE-like transporters must mediate influx, and thus are predicted to be present in the luminal (blood facing) membrane. In the first scheme, suggested by Taylor et al. [336], NBCe1 would also be located in the luminal membrane mediating influx while AE2 would be located in the abluminal membrane mediating HCO_3_^−^ efflux in exchange for Cl^−^. Thus all the HCO_3_^−^ transporters would be moving HCO_3_^−^ in the direction required for secretion, but there would be no obvious reason why three NBC-like transporters are required. The second scheme incorporates the suggestion by O’Donnell [19] that AE2 is located in the luminal membrane. The HCO_3_^−^ efflux route from the cell would then be provided by NBCe1. This scheme places the HCO_3_^−^ transporters in positions analogous to those seen in the choroid plexuses but AE2 is now redundant to the extent that no matter how much it transports changes in the other HCO_3_^−^ transporters can compensate. Both schemes are plausible. Further experimental results are required. It should be noted that in our present state of ignorance (see especially Sect. 4.4.1) other schemes are conceivable in which the HCO_3_^−^ transporters in schemes 1 or 2 are placed in the opposite membranes. These schemes would produce secondary active transport of HCO_3_^−^ from ISF to blood as envisaged many years ago by Pappenheimer, Fencl and colleagues [351, 352].

NKCC1 brings in Cl^−^ across the luminal membrane and AE2 will also transport Cl^−^ inward across the membrane in which it is located. Thus there must be an additional Cl^−^ transporter in the abluminal membrane to move Cl^−^ from the cells to the brain as part of any secretion. Furthermore because NKCC1 brings in two Cl^−^ for each Na^+^, if this mechanism accounts for a large proportion of the luminal Na^+^ entry, more Cl^−^ will enter than can be part of any possible transcellular net flux. An additional Cl^−^ transporter is required in the luminal membrane to recycle the excess Cl^−^.

Regulation of HCO_3_^−^ transport and its interrelations with H^+^, Cl^−^ and CO_2_ transport are considered in Sect. 6.

#### Role of carbonic anhydrase in HCO_3_^−^ transport

Carbonic anhydrase is present both inside [336] (isoform not yet identified) and, on at least one surface, (CAIV [353, 354]) of brain endothelial cells (at least in rat). Neither scheme 1 nor 2 provides an explanation for why carbonic anhydrase might be required for normal secretion as there is no need for the hydration-dehydration reactions between CO_2_ and H_2_CO_3_. The in vivo experiments showed no effect of carbonic anhydrase inhibitors on tracer fluxes, however, these results do not reveal whether or not there is an effect on net fluxes (see Sect. 4.3.5). Further experiments are required.

#### K^+^ transport

K^+^ is loaded into the cells from both sides: from the blood by NKCC1 and to some extent by the Na^+^-pump (see Sect. 4.4.1) and from the ISF by the Na^+^-pump. On each side the influx exceeds the net flux across the cells, and thus at each membrane there must be pathways for efflux of most of the K^+^ that enters. These are thought to be K^+^ channels (see Sect. 4.5.3). K^+^ transport is considered further in Sect. 5.

### A description of Na^+^, Cl^−^ and water transport across the blood–brain barrier

So far transport across only the endothelial layer of the blood–brain barrier has been discussed. To cross into the brain parenchyma solutes and water must cross not only the endothelial cells but also the surrounding basement membrane and the layer of astrocyte endfeet. There is little reported data about channels or transporters for Na^+^ or Cl^−^ or the presence and activity of the Na^+^-pump in the astrocyte endfeet. In the absence of any such evidence it would appear that the fluxes of these ions between blood and brain parenchyma pass through the clefts between the endfeet. This is scheme (1) in Fig. 4.

The importance of water fluxes via aquaporin 4 (AQP4) in the endfoot membrane under normal circumstances has not been established. In mice in which AQP4 has been knocked out in astrocytes the endfeet are swollen. This observation led Amiry–Moghaddam et al. to suggest that AQP4 is needed to allow efflux of metabolically produced water [355]. Regardless of whether or not this swelling is due to accumulation of metabolic water, the idea that AQP4 might be mediating an efflux of water from the astrocytes under normal conditions deserves further consideration.

It was argued in Sect. 4.1 that there is a strong circumstantial case that there is a net secretion of fluid from blood to brain across the blood–brain barrier. It was argued at the start of Sect. 4 that the ratio of the amount of NaCl transported to the amount of water transported must be higher, possibly much higher (see also footnote 8), than in an isosmotic solution because when the secretion is diluted with metabolically produced water the net product is nearly isosmotic. If, as is very likely, the interior of the astrocytes is nearly isosmotic, secretion of hyperosmotic fluid into the basement membrane by the endothelial cells would imply that the osmotic gradient across the AQP4-containing membranes is directed from endfoot towards the basement membrane—i.e. AQP4 would be mediating a net flux of water into the basement membrane. This water when combined with the hyperosmotic endothelial cell secretion would produce a product that is not so strongly hyperosmotic, which would then emerge into the interstitial spaces of the brain by way of the clefts. This is scenario 3 in Fig. 4. If the net movement of fluid across the blood–brain barrier is a hypoosmotic absorption rather than a hyperosmotic secretion, then the source of the excess water is likely to be efflux from astrocyte endfeet much of which would be via AQP4.

The function of AQP4 is to increase water permeability. High functional expression of AQP4 would allow a given osmotic gradient to drive a larger flux of water or a given flux of water to be driven by a smaller osmotic gradient. The osmotic gradient across the endfoot membrane is likely to be small because the parenchyma as a whole is known to be nearly isosmotic with blood and the water permeability of the endothelium is sufficiently high (see Sect. 4.3.6) that the osmolality within the basement membrane will also be close to that of plasma. As mentioned above, it is known that in the absence of AQP4 the endfeet swell [355], as if an efflux of water has been blocked. Thus it appears that AQP4 is needed for the second reason: to allow a small gradient to drive a net water efflux from the endfeet.

Both AQP4 and the potassium channel subunit Kir4.1 are highly expressed in the astrocyte endfeet [356–358]. The possible implications of this are discussed further in Sect. 5.3.

## Role of the brain interfaces in regulation of K^+^ in the brain

K^+^ levels in the brain are remarkably stable with [K^+^]_ISF_ less than [K^+^]_plasma_ [16, 246–248, 253, 359–361]. By taking up K^+^ from ISF, astrocytes can limit increases in [K^+^]_ISF_ resulting from nervous activity inside the brain, but such uptake or release cannot protect the brain as a whole from long-term changes in [K^+^]_plasma_. For instance if the rate of entry is increased due to an increase in [K^+^]_plasma_, the astrocytes and neurons could initially take up K^+^ so reducing the increase in [K^+^]_ISF_, but such a process has a finite capacity and eventually net uptake into the brain cells must cease.

**Fig. 19:**
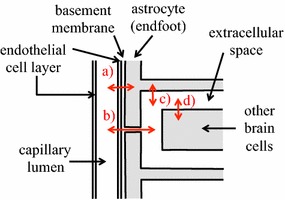
Schematic diagram of K^+^ exchanges that are thought to be most important in regulation of [K^+^]_ISF_ and brain K^+^ content: **a** between plasma and astrocytes via the endothelial cells, the basement membrane surrounding them and K^+^ channels in astrocyte endfoot membranes; **b** between plasma and ISF via the endothelial cells, the basement membrane and clefts between the endfeet; **c** between astrocytes and ISF; and **d** between ISF in the extracellular space and brain cells

There are only two ways to reach a new steady-state in the brain in the face of a sustained increase in [K^+^]_plasma_: either decrease the rate of entry from the blood or increase the rate of exit from the brain. In other words the long-term steady-state for K^+^ in the brain is dependent on transport processes at the blood–brain interfaces together with K^+^ loss as part of fluid outflow from the brain. Of these routes, transfers across the blood–brain barrier, (a) and (b) in Fig. 19, are thought to be most important because measurements show that far more K^+^ enters and leaves the parenchyma across the blood–brain barrier than enters CSF across the choroid plexuses [16, 250, 253, 261] (see Sect. 4.3.1) or leaves in the fluid outflow. In addition it has been shown in the rat that regulation of [K^+^]_ISF_ develops at an earlier age than that of [K^+^]_CSF_ [247]. Nevertheless it is clear that the rate of K^+^ secretion by the choroid plexuses is also regulated.

### K^+^ transport across the choroid plexuses

In studies on the composition of choroid plexus secretion, [K^+^] (~3.5 mmol kg^−1^) in the secretion was remarkably stable in the face of acute changes in either [K^+^]_plasma_ and/or CSF secretion rate [141, 200, 247, 362]. Current knowledge of the transporters present in the choroid plexuses is consistent with this observation but can hardly be held to predict it.

Sampling choroid plexus secretion directly is difficult though not impossible and there have been two such studies [200, 362] (see Sect. 6.4.1). Useful information can be obtained more readily by sampling CSF from the ventricles and cisternae. Using this method, Stummer et al. [361] measured the flux of ^86^Rb^+^ from blood to CSF at low, normal and high [K^+^]_plasma_. They collected a large fraction of the total CSF from rats (100 µl samples) 10 min after introduction of the tracer into the blood. This time interval they argued was insufficient for there to be appreciable entry of tracer into the CSF via the blood–brain barrier and parenchyma. The results they obtained demonstrate a clear difference in ^86^Rb^+^ accumulation between acute (minutes) and chronic (days) variations in [K^+^]_plasma_ [361]. With acute variations the K^+^ influx, calculated from ^86^Rb^+^ accumulation, increased with [K^+^]_plasma_. By contrast, after [K^+^]_plasma_ had been maintained at different levels for more than a week, the K^+^ influx was independent of [K^+^]_plasma_.

The observation that acutely K^+^ influx increases with [K^+^]_plasma_ is expected since much of the influx is thought to be paracellular (see Sect. 3.6.4). The observation that K^+^ influx does change while the [K^+^] in the secretion does not implies that there is an increased efflux of K^+^ from the secretion back to blood. The most likely mechanism for this is that K^+^ entering via the paracellular route produces an increase in [K^+^] within the secretion, too small or too local to be measured, and this increase somehow produces a disproportionately large increase in transfer of K^+^ from the secretion back into the epithelial cells, probably via the Na^+^-pump (compare with Sect. 4.3.1), and hence to the blood.

How the transcellular and paracellular routes of transfer (see Sect. 3.6.4) are regulated to achieve an unchanged influx from blood to CSF in the face of a long-term change in [K^+^]_plasma_ is unclear. The suggestion made by Stummer et al. [361] that there was downregulation of a transporter that mediates K^+^ transfer into the secreted fluid presumed that the influx was transcellular while the available evidence implicates a large paracellular component. Klarr et al. [363] found that in choroid plexuses isolated from hypo-, normo- or hyper-kalemic rats both expression of the α_1_ and β_1_ subunits of the Na^+^, K^+^- ATPase and ouabain sensitive K^+^ uptake into the epithelial cells increased with [K^+^]_plasma_ and pointed out that similar change in transfer of K^+^ from nascent CSF to blood by the Na^+^-pumps in vivo would be in the correct direction to help stabilize [K^+^] in the secretion. This could appear as a reduction in influx, if K^+^ emerging from either the transcellular or paracellular routes were transported back into the epithelial cells so efficiently, that a significant portion would not be able to diffuse away into CSF and hence be counted as part of the influx. Changes in the paracellular route, i.e. in the tight junctions, have yet to be considered as part of the explanation.

### K^+^ transport across the blood–brain barrier

In vivo studies on K^+^ transport across the blood–brain barrier and regulation of [K^+^]_ISF_ were considered in Sects. 4.3.1 and 4.3.4 and evidence from in vitro studies and the mechanisms for transport across the endothelial cells were discussed in Sects. 4.5.1, 4.5.3 and 4.6.4. It should be noted that because the in vivo flux experiments all used measurements of the K^+^ content of the parenchyma their results do not distinguish between transfers between blood and astrocyte endfeet, (a) in Fig. 19, on the one hand and between blood and ISF, (b) in Fig. 19, on the other.

**Fig. 20:**
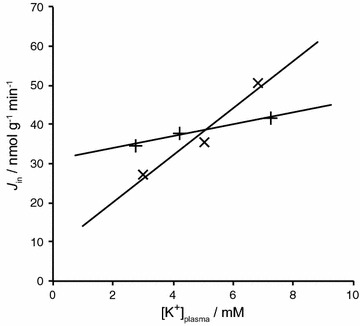
Tracer influx of K^+^ into brain parenchyma with acute (X) or chronic (+) variations in plasma [K^+^] as calculated by Stummer et al. [361] from their data for ^86^Rb^+^ entry

Alongside their experiments on influx into CSF, Stummer et al. [361] compared influx of K^+^ into the brain parenchyma for acute (minutes) and chronic (days) variations in [K^+^]_plasma_ (see Fig. 20). With acute variations the rate of accumulation (calculated from ^86^Rb^+^ uptake) increased with [K^+^]_plasma_ while with chronic changes any variation with [K^+^]_plasma_ was much smaller. Stummer et al. suggest that the difference between the responses to acute and chronic changes indicates that chronically there is regulation of the number of transporters mediating influx, with the number decreasing as [K^+^]_plasma_ increases. The obvious candidate at the blood–brain barrier is NKCC1 but that would also affect transport of Na^+^ and Cl^−^. Another possible explanation is considered in footnote.^20^ Unfortunately there is no other evidence to suggest which transporter is regulated, let alone how. There is evidence for down regulation of the α_3_ subunit of the Na^+^, K^+^-ATPase in hyperkalemia [364], but no obvious way that this result can be used to explain the data for ^86^Rb^+^ influx.

In theory the arrangement of transporters at the blood–brain barrier shown in Figs. 17 or 18 could produce a net transport of K^+^ in either direction depending on the K^+^ concentrations, the potential difference across the barrier and the relative activities of the different transporters and channels present. Because the net flux across each membrane is the small difference between relatively large influx and efflux, subtle changes in any factors affecting these fluxes could produce large changes in the net flux across the cells. For instance the variation of Na^+^-pump rate with [K^+^] in the basement membrane may have a large effect on the net flux of K^+^ (see Sects. 4.3.1 and 5.3).

In summary: on a time scale of seconds, astrocytes buffer [K^+^]_ISF_; on a time scale of minutes to hours, [K^+^]_ISF_ is protected against changes in [K^+^]_plasma_ by marked increases in K^+^ efflux for small changes in [K^+^]_ISF_; and on a time scale of days, the regulation is improved further by changes in the processes that allow K^+^ to enter across the blood–brain barrier when [K^+^]_plasma_ is increased.

### The role of K^+^ channels in glial endfeet

K^+^ channels containing Kir4.1 subunits are prominently expressed in the endfoot membrane facing the endothelial cells [365, 366]. Furthermore there is evidence from studies on neurovascular coupling (see end of Sect. 2.3) suggesting that release of K^+^ from endfeet does occur following neuronal activity and thus that the channels are functionally active. How and why are K^+^ channels localised especially (though not exclusively) in these endfeet?

AQP4 (see Sect. 4.7) and Kir4.1 colocalize to the endfoot membrane facing the basement membrane (or basal lamina) by binding to different components of the dystrophin-associated protein complex, which also binds to laminin in the basement membrane [356–358]. However, the presence or absence of AQP4 does not appear to affect the properties of the Kir4.1 channels [367, 368], though there may be functional interaction in the parenchyma because water fluxes may change the time course of changes in [K^+^] [369]. However, there is no evidence that this happens in the basement membrane of the blood–brain barrier.

One of the established functions of the selective K^+^ conductance of astrocyte membranes is the so-called ‘spatial buffering’ mechanism that can redistribute extracellular K^+^ [370–373]. K^+^ released from active neurons causes local elevation of [K^+^]_ISF_ and consequent entry of K^+^ into astrocytes, leading to depolarisation and inward current that spreads to other regions of the astrocyte syncytium. The current carried by K^+^ leaves the astrocytes where [K^+^]_ISF_ is less elevated. In effect, excess K^+^ is spread out by a passive mechanism over a larger volume, thus reducing the change in [K^+^]_ISF_ locally. Such redistribution (and subsequent return by the same mechanism) would tend to happen anyway by extracellular diffusion, but can be enhanced fivefold over large distances as seen in rabbit cortex [372]. For some disturbances (see Fig. 5 in [374]) spatial redistribution can be the dominant buffering mechanism for [K^+^]_ISF_ close to active neurons.

A special form of spatial buffering, shown to occur in retinal glial (Müller) cells is K^+^ ‘siphoning’ [373, 375–377]. K^+^ channels in Müller cells are particularly concentrated at the inner and outer retinal surfaces where the large fluid volumes appear to act as sinks to buffer concentration changes within the densely packed neural layers of the retina. Since glial K^+^ channels in both retina and mammalian brain are also concentrated in the endfeet surrounding blood vessels, it is natural to suggest that these may serve the function of siphoning excess K^+^ to or from capillaries where they may be exchanged with blood or via perivascular routes to CSF (see discussion in [378]). However, significant K^+^ efflux across the blood–brain barrier is not easily detected during even extreme nervous activity [379] and at this site such mechanisms may be too slow to play a significant part in the dynamics of K^+^ buffering over periods of seconds or even minutes (see [372], section 5.3.1 in [16], and section III in [380]).

With such small blood–brain barrier fluxes, Gardner–Medwin (personal communication) has argued that concentrating K^+^ channels close to capillaries would have little or no benefit in [K^+^]_ISF_ buffering compared with uniform distribution of the channels over the entire astrocyte membrane and reliance on simple diffusion of K^+^ to capillaries. Instead he suggests that the reason why localization of Kir4.1 matters is that this combined with the almost complete coverage of the endothelial tube by endfeet may be important for the longer term homeostatic regulation of blood–brain barrier K^+^ transport. In this view the concentration (strictly the electrochemical potential) of K^+^ in the endfeet is taken to be a more reliable measure of the long-term K^+^ status of the brain than the fluctuating and unrepresentative [K^+^]_ISF_ in the nearby extracellular space. Keeping K^+^ in the basement membrane close to equilibrium with K^+^ in the astrocytes rather than with K^+^ in the extracellular spaces (see Fig. 19) ensures that the transport across the blood–brain barrier reflects the concentrations and amounts of K^+^ over a wide region of parenchyma. If this is correct, the reason why a high density of channels and hence high K^+^ conductance is required in the endfoot membrane is not so much to allow a large K^+^ flux but rather to reduce to nearly negligible levels the electrochemical gradient required to drive the small net K^+^ flux that occurs (compare with the discussion of the role of AQP4 in Sect. 4.7).

## pH and concentration of HCO_3_^−^ in the extracellular fluids of the brain: importance of HCO_3_^−^ transport at the blood–brain barrier and choroid plexuses

This section considers the extent to which the blood–brain interfaces are involved in determining the pH of the extracellular fluids of the brain.

**Fig. 21:**
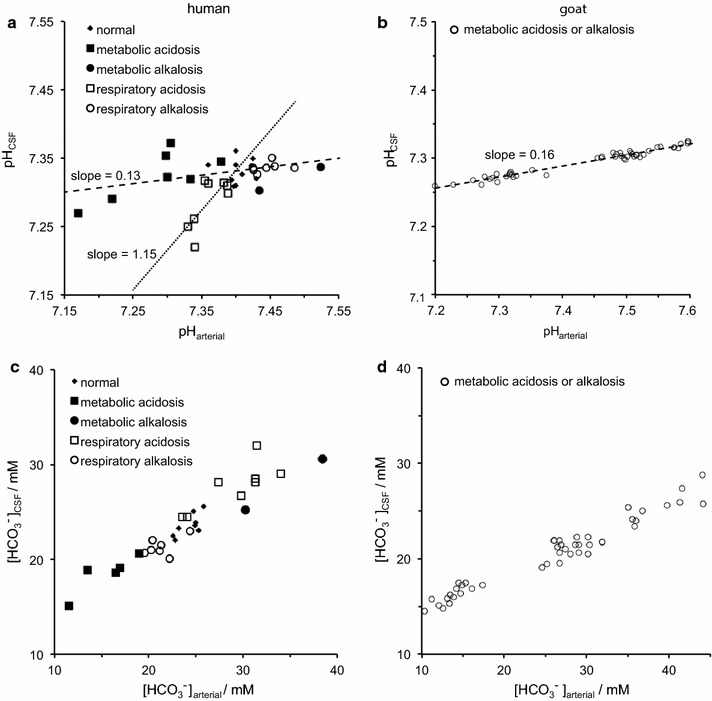
Classic studies on the regulation of CSF pH. pH (**a**, **b**) and [HCO_3_^−^] (**c**, **d**) in CSF are plotted against values of the same parameters in arterial blood plasma. **a**, **c** are for humans with acid–base disorders as indicated (taken from the compilation in Table 2 of [52] with all of the data shown). **b**, **d** are for goats exposed to different [HCO_3_^−^]_arterial_ over a week by systemic administration of NH_4_Cl or NaHCO_3_ (data extracted from Fig. 2 of [352] with pH_arterial_ calculated as in their Fig. 3). pH is regulated by controlling the ratio [HCO_3_^−^]/pCO_2_ (see Sect. 6.1.1). A metabolic disturbance of pH is one in which the causal event is a change in [HCO_3_^−^] while a respiratory disturbance of pH is one in which the causal event is a change in pCO_2_. All of the data reported for goats are for metabolic disturbances. As can be seen in both humans and goats, in metabolic acidosis and alkalosis (*dashed lines*) pH_CSF_ changes by much less than pH_arterial_, i.e. there is tighter regulation of pH_CSF_. By contrast in humans in respiratory acidosis (*dotted line*) the variation in pH_CSF_ is as large or larger than the change in pH_arterial_. In metabolic acidosis and alkalosis the tighter control of pH_CSF_ is a consequence of the smaller variation in CSF of [HCO_3_^−^] (see **c**, **d**) and hence of the [HCO_3_^−^]/pCO_2_ ratio than in arterial plasma. More recent data confirm the variations shown for metabolic disturbances and the general features of the responses to respiratory disturbances [185]). The relations between changes in [HCO_3_^−^] and changes in pH are considered further in footnote 21

The concentration of H^+^ in extracellular fluid in general is small compared to those of any other ions considered in this review. Nevertheless even these small concentrations are important because H^+^ can bind reversibly to many body constituents altering their net charges with resultant effects on their function. To provide a stable environment in which cells can control their intracellular pH, extracellular pH is regulated to fall within a relatively narrow range, 7.35–7.45 (see e.g. pp. 224 and 225 in [381]) corresponding to [H^+^] 45 and 35 nmol kg^−1^ respectively. This is done by controlling the concentrations of CO_2_ and HCO_3_^−^ (see Sect. 6.1.1). In most tissues of the body, CO_2_ and HCO_3_^−^ exchange freely across capillary walls and thus regulation of plasma pH controls extracellular pH. The brain is different: CO_2_ still moves freely between plasma and ISF, but movements of HCO_3_^−^ are governed by the function of specific transporters at the blood–brain interfaces. Under many circumstances this reduces variation of pH within the extracellular fluids of the brain (see Fig. 21 and 21) allows closer regulation (see e.g. [382–385]) although Siesjö [382] discusses the important point that control of plasma pH still accounts for a large part of the control of CSF and ISF pH.

Regulation of pH and [HCO_3_^−^] in ISF and CSF has been the subject of many reviews including [16, 59, 382, 386–390]. Two major conferences highlighted the controversies in the early work [391, 392]. Kazemi and Johnson [393] and Fencl [185] gave comprehensive cover of work up to 1986 and Davson [17] and Nattie [394] gave accounts as of the late 1990s. The effects of many transport inhibitors are discussed in [393]. The subject has been surveyed more recently by Nattie [395]. It is remarkable that other reviews, those that refer to “brain pH” rather than intracellular and extracellular pH (see e.g. [385, 396]), largely ignore regulation of extracellular pH even though this must affect the regulation of intracellular pH.

Control of ISF pH is more important than that of CSF pH (see e.g. [382, 386, 392, 397]. This is primarily because it is the ISF that comes into direct contact with cells within the brain parenchyma. However, because it is much easier to measure the composition of CSF, many of the available results relate to this. Fortunately it is generally accepted that in the steady-state, the ionic compositions of CSF and ISF are closely similar but with somewhat higher pCO_2_, lower pO_2_ and lower glucose concentration in ISF than in CSF [351, 352].

In ISF pCO_2_ is determined by the rate of production of CO_2_ by brain cells and the net rate at which CO_2_ is removed in the blood (see Sect. 2.2). In CSF pCO_2_ is typically 7–9 mmHg higher than in arterial blood [386] and presumably slightly higher still within the parenchyma where the CO_2_ is produced. These differences will vary with blood flow [386]. However the available evidence indicates that the *change* in the difference between pCO_2_ in ISF and blood is of relatively minor importance in understanding the changes in pH within the brain when the composition of arterial blood is altered [185, 386, 393]. In the following discussion it will be assumed that *changes* in pCO_2_ in arterial blood will quickly produce similar *changes* in pCO_2_ throughout the brain.

**Fig. 22:**
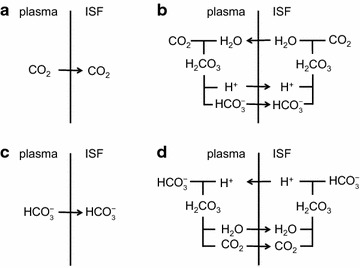
Transport schemes for CO_2_ (**a**) and (**b**), and for HCO_3_^−^ (**c**) and (**d**). Transport of CO_2_ and HCO_3_^−^ can be distinguished since the latter but not the former entails the transport of net charge. By contrast transport of HCO_3_^−^ in one direction and transport of H^+^ in the other can produce the same net result. See text for further explanation. The proportion of CO_2_ transport that occurs by the mechanism in (**b**) is negligible

CO_2_ is able to cross membranes either by diffusion as molecular CO_2_ (scheme a, Fig. 22a) or by a more complicated sequence of steps (scheme b, Fig. 22b) in which the CO_2_ first hydrates to form H_2_CO_3_, the H_2_CO_3_ dissociates to form H^+^ and HCO_3_^−^, and then each of these species crosses the membrane, and the H^+^ and HCO_3_^−^ recombine following which the H_2_CO_3_ dehydrates. The water can diffuse back across the membrane leaving the same overall result in both cases: the transfer of a single molecule of CO_2_. The large amounts of CO_2_ formed in the brain, ca. 3.3 mol day^−1^ (see Sect. 2.2), arise from the oxidation of a neutral substrate, glucose. Neither the glucose nor the O_2_ brings net charge into the reaction, so the end product CO_2_ must be disposed of also without altering charge (see Sect. 6.1.2 for further discussion). This condition is satisfied by either scheme a or b. However, because the small, neutral molecule, CO_2_, can cross membranes rapidly (see Sect. 2.2) while transport of the ions, HCO_3_^−^ and H^+^, is relatively slow and usually in opposite directions, scheme a is the dominant process and CO_2_ transport can be discussed without reference to any HCO_3_^−^ or H^+^ fluxes across the membrane.

The net amount of HCO_3_^−^ transferred into the brain across the blood–brain interfaces is only of the order of 11 mmol day^−1^ as estimated from an average value of [HCO_3_^−^] in the secretions, ~22 mM, and a total secretion rate by whatever routes of ~500 ml day^−1^. This is very much less than the amount of CO_2_ transferred. HCO_3_^−^can cross membranes either via transporters with which it interacts directly (scheme c, Fig. 22c) or via a more complicated sequence of steps (scheme d, Fig. 22d) in which the HCO_3_^−^ first combines with a H^+^, the resultant H_2_CO_3_ dehydrates forming CO_2_ and H_2_O, and each then diffuses across the membrane. On the other side the CO_2_ recombines with H_2_O after which the resultant H_2_CO_3_ dissociates to form H^+^ and HCO_3_^−^. If the overall result is to be transport of HCO_3_^−^, the H^+^ must be transported back across the membrane. In both schemes c and d there is a net transfer of one negative charge as part of the HCO_3_^−^ transport. *It is this transport of charge that differentiates the net transport of* HCO_3_^−^ *or of* H^+^ *in the opposite direction from net transport of* CO_2_ *and requires* HCO_3_^−^ *and* H^+^ *transport to be considered separately from* CO_2_ *transport* (as in Sect. 6.1.2).

Both of the schemes in Fig. 22c, d are important. In physiological solutions at near neutral pH, [HCO_3_^−^] is 20–25 mM and [H^+^] is ~0.1 µM. Thus the amount of net charge transferred by transport of either HCO_3_^−^ or H^+^ across the choroid plexuses or the blood–brain barrier will correspond primarily to changes on either side of the barrier in [HCO_3_^−^]. For this reason it is often convenient to talk of a flux of HCO_3_^−^ in one direction even when the underlying mechanism may include a flux of H^+^ in the opposite direction as in Fig. 22d. Furthermore, as indicated in Sects. 3 and 4, the available evidence now strongly favours transport of HCO_3_^−^ itself across the membranes of the choroid plexuses and the blood–brain barrier (and in many other epithelial processes, see e.g. [398, 399]). However, it is important to note that under conditions where intracellular pH is reduced, H^+^ transport, primarily via Na^+^/H^+^ exchange, can be much faster than HCO_3_^−^ transport across one or both of the membranes of the endothelial cells of the blood–brain barrier [268].

HCO_3_^−^ ions can be introduced into or removed from ISF and CSF in a number of ways, five of which are quantitatively prominent:

- buffering of ISF by brain cells most of which entails interconversion of CO_2_ and HCO_3_^−^ within the cells and exchange of HCO_3_^−^ and Cl^−^ across their membranes (see Sect. 6.2);
- production of lactic acid within brain cells and its transfer to the extracellular fluids where the H^+^ is buffered by the CO_2_/HCO_3_^−^ system (see Sect. 6.3);
- removal of lactate^−^ together with H^+^ by MCT1 (equivalent to addition of a HCO_3_^−^) (see Sect. 6.3);
- transport of HCO_3_^−^ (or equivalently of H^+^ in the opposite direction) across the choroid plexuses or the blood–brain barrier (see Sect. 6.4);
- ISF and CSF can themselves be removed by fluid outflow from the brain (see Fig. 8 and [15]).

The first four of these routes are important in transient changes in [HCO_3_^−^], but it is argued in the following sections (and previously by many others) that the fourth and fifth are most important on time scales of hours or longer.

Under normal resting conditions, [HCO_3_^−^] in brain extracellular fluids is less than would occur if it were at equilibrium with [HCO_3_^−^] in plasma. For [HCO_3_^−^]_CSF_ = 22 mM and [HCO_3_^−^]_plasma_ = 24 mM the value of the potential at equilibrium (calculated from the Nernst equation) would be −1 mV, yet the measured PD is between 2 and 7 mV positive [185, 286]. This is even the wrong sign for there to be an equilibrium. There are species variations in [HCO_3_^−^] in CSF and plasma but always it seems with [HCO_3_^−^]_CSF_ too small for there to be an equilibrium [17, 400].

Two possible explanations have been offered for how the lower [HCO_3_^−^] in ISF and CSF is maintained [185, 382]: acid (primarily lactic acid) is continually being added to ISF and CSF converting HCO_3_^−^ to CO_2_ (Sect. 6.3) or there are active transport processes at one or both of the barriers separating CSF and ISF from blood plasma (Sect. 6.4).

### Consideration of the physiological principles important for understanding pH regulation

Before discussing the transport of CO_2_ and HCO_3_^−^ in and out of the brain across the interfaces (Sect. 6.4), it is necessary to consider the interconversion of CO_2_, HCO_3_^−^ and H^+^ and the constraints imposed by the Principle of Electroneutrality and the maintenance of osmotic equilibrium between the brain and blood.

#### The interrelationship of CO_2_, HCO_3_^−^ and H^+^

HCO_3_^−^ and H^+^ ions differ from fixed ions such as Cl^−^ and Na^+^ in that they can be formed in solution from neutral precursors CO_2_ and H_2_O,

2 $$ {\text{CO}}_{2} + {\text{H}}_{2} {\text{O}}  \rightleftarrows {\text{H}}_{ 2} {\text{CO}}_{ 3}   \rightleftarrows  {\text{HCO}}_{3}^{ - } + {\text{H}}^{ + } $$

In physiological solutions these reactions are sufficiently fast and can be regarded as reaching equilibrium (i.e. wherever required carbonic anhydrase is present),

3 $$ K^{{\prime }} \left[ {{\text{CO}}_{2} } \right]\left[ {{\text{H}}_{ 2} {\text{O}}} \right] = \left[ {{\text{HCO}}_{3}^{{ - }} } \right]\left[ {{\text{H}}^{ + } } \right] $$

where *K*′ combines the equilibrium constants for the two reactions shown above. It has no units. Because [CO_2_] is proportional to pCO_2_ and the concentration of water is a constant (55.5 mol kg^−1^), Eq. 3 can be rewritten as

4 $$ K{\text{pCO}}_{2} = \left[ {{\text{HCO}}_{3}^{{ - }} } \right]\left[ {{\text{H}}^{ + } } \right] $$

where *K* is the composite constant.

5 $$ K = K{{^{{\prime }} \left[ {{\text{H}}_{ 2} {\text{O}}} \right]\left[ {{\text{CO}}_{2} } \right]} \mathord{\left/ {\vphantom {{^{{\prime }} \left[ {{\text{H}}_{ 2} {\text{O}}} \right]\left[ {{\text{CO}}_{2} } \right]} {{\text{pCO}}_{2} }}} \right. \kern-0pt} {{\text{pCO}}_{2} }} $$

If the units of [H^+^] and [HCO_3_^−^] are mol kg^−1^ and pCO_2_ is in mmHg, then the units of *K* are mol^2^ kg^−2^ mmHg^−1^. Equation 4 is one form of the Henderson–Hasselbalch equation, which is more commonly encountered in a logarithmic form,

6 $$ {\text{pH}}\,{ = }\,{\text{pK}} + {\rm log}\left( {{{\left[ {{\text{HCO}}_{3}^{{ - }} } \right]} \mathord{\left/ {\vphantom {{\left[ {{\text{HCO}}_{3}^{{ - }} } \right]} {{\text{pCO}}_{2} }}} \right. \kern-0pt} {{\text{pCO}}_{2} }}} \right) $$

where

7 $$ {\text{pH}} =  - \log \left( {\left[ {{\text{H}}^{ + } } \right]} \right) $$

and

8 $$ {\text{p}}K = - \log \left( K \right) $$

In Eqs. 6–8 the various symbols stand for the numerical values of the quantities in Eq. 4 when they are expressed in the units indicated above. Numerical values must be used because taking logarithms of units is not defined.

As a consequence of the reactions described by Eq. 4, once any two of [H^+^], [HCO_3_^−^] and pCO_2_ are known the third can be calculated (at least in principle: the value of *K* must be known for the conditions of the solutions in question). Similarly anything that could be achieved by a flux of one of H^+^, HCO_3_^−^ and CO_2_ could be achieved equally by some combination of fluxes of the other two (see Fig. 22). For brief consideration of OH^−^ and CO_3_^2−^ see 22.

HCO_3_^−^ transport cannot be followed using radiotracers, e.g. H^14^CO_3_^−^, because interconversion of HCO_3_^−^ and CO_2_ and movement of CO_2_ across membranes (see Fig. 22) are much more rapid than transfer of HCO_3_^−^ itself (for a possible exception see Sect. 6.4.2). If H^14^CO_3_^−^ (or H^11^CO_3_^−^) is added to blood, the ^14^C (or ^11^C) quickly distributes itself over all of the HCO_3_^−^ and CO_2_ present. This can occur with or without net transfer of either total CO_2_ (labelled plus unlabelled) or total HCO_3_^−^ [51, 184].

#### Constraints imposed by the principle of electroneutrality and constancy of osmolality

The first constraint is imposed by the need for electroneutrality. It is not possible to add more than a negligible amount of HCO_3_^−^ or any other charged species to a cell without adding or removing something else. The reason is that addition of very few ions produces a large enough electrical potential difference between the cell and its surroundings to prevent further addition or force a compensating movement of something else. The Principle of Electroneutrality is the general statement of this property of charges and potentials. It asserts that the net charge within any region, e.g. a cell or a portion of extracellular fluid, will always be so small that it cannot be determined by any means other than measuring the potential difference between that region and its surroundings, i.e. to the accuracy it can be measured by all other means the net charge is zero.

It is important to realize that the Principle of Electroneutrality applied to any region is an approximation. The net charge within a cell can be exactly zero only if there is no potential difference between it and its surroundings. What the Principle of Electroneutrality states is that the net charge is very small compared to either the total charge on the cations or the total charge on the anions. When the imprecisely-known total charge on the anions is subtracted from the imprecisely-known total charge on the cations, the difference is smaller than the errors. Within experimental accuracy it is possible to say that the sum of charges on the cations must be equal to the sum of charges on the anions.

The accuracy of the electroneutrality approximation applied to a region increases with the total concentrations of the ions present and the size of the region. Because the numbers of ions present scale with volume while the potential difference produced by the net charge scales with the surface area (and capacitance), the electroneutrality assumption becomes less secure as the size of the region becomes smaller. Electroneutrality is exactly true only when there are no electrical potential differences, but it is true to an excellent approximation whenever the volume of the region is sufficiently large. In practical terms with physiological solutions electroneutrality fails when the size of the region becomes very small, roughly the size of a mitochondrion. However, the assumption is secure (and can be called a “Principle”) for regions as large as a mammalian cell.

For the extracellular fluids of the brain electroneutrality implies that

9 $$ \left[ {{\text{Na}}^{ + } } \right] + \left[ {K^{ + } } \right] = \left[ {{\text{Cl}}^{-} } \right] + \left[ {{\text{lactate}}^{- } } \right] + \left[ {{\text{HCO}}_{ 3}^{ - } } \right] $$

This must also be true in both the fluids lost from the brain and the fluids secreted into it. [lactate^−^] is included in this expression because in hypocapnia/hypoxia it can increase to significant levels (see Sect. 6.3). This statement is an approximation both because electroneutrality is itself an approximation but much more importantly because ionic species present at low concentrations, e.g. Mg^2+^, Ca^2+^ and various phosphate compounds, have been ignored. This can be justified for the present purpose, the illustration of principles, because variations in the concentrations of the ignored species are small compared to variations in [HCO_3_^−^]. The net charge on proteins can be ignored for extracellular fluids in the brain but not for plasma or intracellular fluids.

The second constraint involves the constancy of osmolality resulting from the relatively free movement of water. Water moves more easily than osmotically active solutes across the choroid plexuses and the blood–brain barrier. Thus to a good approximation water moves until the osmolality of ISF and CSF are the same as that of plasma, *C*_osmolality_. Approximately this means

10 $$ \begin{aligned}&\left[ {\text{Na}^{ + } } \right] + \left[ {\text{K}^{ + } } \right] + \left[{{\text{HCO}}_{3}^{{ - }} }\right]\\
&\qquad\,\,+\left[ {{\text{Cl}}^{- } } \right]  +\left[ {{\text{lactate}}^{- } } \right] = C_{\text{osmolality}} \end{aligned}$$

The combination of the two constraints, Eqs. 9 and 10, leads to

11 $$ \begin{aligned}2 \times \left( {\left[ {{\text{Na}}^{ + } } \right] + \left[ {{\text{K}}^{ + } } \right]} \right) &= 2 \times \left( {\left[ {{\text{HCO}}_{3}^{{ - }} } \right] + \left[ {{\text{Cl}}^{ - } } \right] + \left[ {{\text{lactate}}^{ - } } \right]} \right)\\
& = C_{\text{osmolality}} \end{aligned}$$

**Fig. 23:**
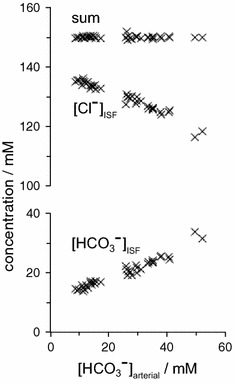
The relation between [HCO_3_^−^]_CSF_ and [Cl^−^]_CSF_ in chronic experimental metabolic acidosis and alkalosis in goats. The sum of [HCO_3_^−^] and [Cl^−^] is very nearly constant. Redrawn from data abstracted by V. Fencl (see [401]) from [351, 352]

As a consequence of this relation if there is any change in [HCO_3_^−^] in CSF or ISF there will also be an equal but opposite change in [Cl^−^] + [lactate^−^]. For examples of experimental data for which [HCO_3_^−^] + [Cl^−^] is constant (see Fig. 23) below, discussion by Fencl following [401] and [402–404]. For [HCO_3_^−^] + [Cl^−^] + [lactate^−^] constant see [405]. For evidence that things may not be quite so simple, e.g. that osmolality may not remain constant in acute changes and/or that [Na^+^] may change, see [403, 406].

Electroneutrality, constancy of osmolality and the reactions relating H^+^ and HCO_3_^−^ to CO_2_ and H_2_O mean that neither the concentrations of H^+^, HCO_3_^−^ and Cl^−^ nor their net fluxes can be manipulated independently. A particular, restrictive description of this interdependence was developed by Stewart [407, 408] in the 1980s and used in the two most recent comprehensive reviews of extracellular pH regulation in the brain [185, 393]. For the reasons discussed in 23 Stewart’s description is not used here.

*In summary any imbalance in charge due to an influx of either HCO*_3_^−^ *or Cl* ^−^ *will produce an electrical potential difference that will decrease influxes of anions and increase those of cations sufficiently to ensure the maintenance of electroneutrality. Any imbalance of osmolality will lead to a water flux to restore balance.*

### Contribution of physiological buffering by brain cells to regulation of ISF pH

If pCO_2_ in arterial blood is increased, CO_2_ rapidly penetrates the brain and enters the astrocytes and neurons where CO_2_ and H_2_O are converted to H^+^ and HCO_3_^−^. Most of the H^+^ is buffered, mainly by intracellular proteins and phosphate compounds. Some of the HCO_3_^−^ leaves the cells in loosely-coupled (e.g. connected by electrical potential changes) or tightly-coupled (e.g. via AE2) exchange for Cl^−^. The change in [HCO_3_^−^]_ISF_ reduces the change in pH_ISF_ associated with the increased pCO_2_. The whole process is closely analogous to the buffering of blood plasma by red blood cells [409, 410].

This physiological buffering of ISF by brain cells is important in the initial changes in [HCO_3_^−^]_ISF_ and pH_ISF_ following changes in arterial pCO_2_ [185, 393, 394, 411–415] (but see [395]). However, in the face of movements of HCO_3_^−^ across the blood–brain interfaces, this process can only produce a transient change in [HCO_3_^−^]_ISF_ (see e.g. [185, 388]) because the cells cannot continue to take up or release HCO_3_^−^. Two examples [415, 416] of studies in which physiological buffering appears to be prominent are considered in 24.

### Impact of lactic acid production and removal on ISF pH

Lactic acid production in brain cells and its export to ISF has been considered as an explanation for the normal non-equilibrium distribution of HCO_3_^−^ between ISF and plasma [185, 382, 417–419]. From current evidence, it is likely to contribute to the setting of ISF and CSF pH when arterial pCO_2_ is greatly reduced and/or in hypoxia. (It should be noted that almost all of the generation of acid by metabolism occurs as production of CO_2_, which is eliminated as such.)

In all of the following lactate means the total amount of lactate present whether as undissociated lactic acid or lactate^−^, i.e. [lactate] = [lactic acid] + [lactate^−^]. In solution most of the lactic acid dissociates and [lactate^−^] ≫ [lactic acid].

Lactic acid production has been calculated from the arteriovenous difference in [lactate] and cerebral plasma flow but sufficiently accurate data have been difficult to obtain and the sampling sites chosen for the venous measurements will usually have ignored outflow other than via venous blood (see below and 25). Under normal conditions the balance of such evidence suggests that lactic acid production rate is 20–40 µmol min^−1^ (see e.g. [48, 420]), which is 5–10% of the rate of glucose consumption (see Sect. 2.4.1) [48]. The conclusion that there is lactic acid production is supported by other types of data including comparison of the rates of utilization of glucose and O_2_ (for references see [48, 421]). Lactic acid production rate might thus normally be as much as three to sixfold greater than the rate, at which HCO_3_^−^ is secreted into the brain (the latter calculated as 22 mM × 500 ml day^−1^ = 11 mmol day^−1^ = 5.9 µmol min^−1^).

Lactic acid production increases in conditions of low pCO_2_ [185, 382, 393, 405, 417–419]. When pCO_2_ is reduced from 37 to 18 mmHg, [lactate^−^]_CSF_ increases from about 1.6–3.2 mM [422]. At these concentrations the rate at which lactate is removed by MCT1 (see below) will be roughly in proportion to the concentration difference between ISF and plasma (see below). As [lactate^−^]_plasma_ is about 1 mM, this suggests a three to fourfold increase in the rate of lactic acid production (compare the discussion following [401]).

If [lactate^−^] increases, electroneutrality and constant osmolality require that [HCO_3_^−^] + [Cl^−^] must decrease by a similar amount (see Eq. 11 and following). It has sometimes been argued that increased [lactate^−^] *causes* a decrease in [HCO_3_^−^] [185, 393] (see also footnote 23) but that argument does not take into account possible changes in [Cl^−^] and does not provide any basis for considering the mechanisms that bring about changes in [HCO_3_^−^]. Such mechanisms and the fact that the changes in [HCO_3_^−^] resulting from lactic acid production need not equal the changes in [lactate^−^] are considered in the following paragraphs.

Acutely when additional lactic acid is added to ISF, the lactic acid dissociates releasing lactate^−^ and H^+^. Almost all of the H^+^ combines with HCO_3_^−^ forming CO_2_, which diffuses away. Thus, as the lactic acid is added and lactate^−^ is accumulating, the HCO_3_^−^ removed equals the lactate^−^ added and the initial changes in [lactate^−^] and [HCO_3_^−^] will be equal but opposite (see e.g. [423, 424]). However, the result can be quite different in the steady-state when [lactate^−^]_ISF_ is constant as described below.

**Fig. 24:**
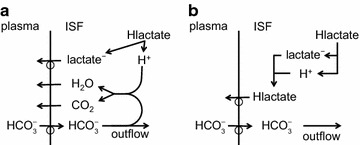
Consequences of adding lactic acid to ISF. In **a** the lactic acid dissociates and the lactate^−^ is transported out of the ISF leaving the H^+^ behind. H^+^ combines with HCO_3_^−^ and forms CO_2_ and H_2_O both of which can diffuse into the blood. Each molecule of lactic acid added reduces the number of HCO_3_^−^ ions present in ISF by one. In **b** the lactic acid is transported out of the ISF to blood as lactic acid (or by cotransport of lactate^−^ and H^+^). There is no immediate relationship between [HCO_3_^−^]_ISF_ and either the rate of addition of lactic acid or [lactate^−^]_ISF_

Sustained production of lactic acid may or may not produce a sustained reduction in [HCO_3_^−^]_ISF_ (see Fig. 24). In the steady-state, lactic acid entering ISF from the cells will dissociate producing lactate^−^ and H^+^. The H^+^ will react with HCO_3_^−^ entering the brain from the blood. This reaction will remove some of the HCO_3_^−^, leaving H_2_O, CO_2_ and lactate^−^. If these products are removed *without regenerating* HCO_3_^−^ *and* H^+^ as in Fig. 24a, [HCO_3_^−^]_ISF_ will be below that seen in the absence of lactic acid production. Because the increase in lactic acid production can be relatively large compared to the rate of secretion of HCO_3_^−^, H^+^ release from lactic acid could be sufficient to result in a large decrease in [HCO_3_^−^]_ISF_ even if removal of lactate^−^ were fast and thus [lactate^−^]_ISF_ were small ([382, 417–419], and discussion following [401]). Indeed, from the estimates of normal lactic acid production rate and of HCO_3_^−^ secretion rate given at the start of this section, if all of the lactate^−^ were removed from ISF leaving the H^+^ behind as in Fig. 24a, there would be far more H^+^ than could be removed by reaction with HCO_3_^−^. *It is important that when lactic acid is added to ISF and dissociates either* (*a*) *the* H^+^ *released can be removed by some means other than reaction with* HCO_3_^−^ *or* (*b*) *the rate of secretion of* HCO_3_^−^ *can be increased.*

l-Lactate^−^ transport across the blood–brain barrier is now known to be mediated by MCT1 that transports one H^+^ for each lactate^−^ [425–427]. The H^+^ transported is supplied by conversion of CO_2_ to HCO_3_^−^ *thus regenerating the* HCO_3_^−^ *lost when the lactic acid was added to the ISF*. As indicated in Fig. 24b, the combination of release of lactic acid into ISF from brain cells and its removal from the brain across the blood–brain barrier by MCT1 does not leave H^+^ for reaction with HCO_3_^−^ and thus can not account for a long-term reduction in ISF [HCO_3_^−^] [417, 418].

Although MCT1 transport of lactate^−^ and H^+^ across the blood–brain barrier is equivalent to removal of lactic acid, it is still convenient to refer to it as transport of lactate because [lactate^−^] is much greater than [lactic acid] in ISF and plasma and thus the concentration changes seen as a result of transport are primarily those of lactate^−^. At low concentrations the rate constant for lactate transport, i.e. (rate of change of [lactate^−^])/[lactate^−^], is about one-third of that for glucose transport mediated by GLUT1 [41, 428–431]. Lactate transport via MCT1 is saturable [430] with a Michaelis–Menten constant near 2.5–3 mM [428, 432]. Thus, even with the increased lactic acid production seen in all but extreme hypocapnia or hypoxia, the mechanism for efflux of lactic acid across the blood–brain barrier is not saturated. However, in severe hypoxia or locally in response to nervous activity, lactic acid production can exceed the capacity for removal by MCT1.

During increased nervous activity, lactic acid production is increased (see [56, 57] for references). This would be expected to increase efflux of lactic acid across the blood–brain barrier, and this would tend to make the arteriovenous difference in [lactic acid] more negative. However, this may not be observed if, for any reason, [lactic acid] in blood is also increased so increasing influx and tending to make the arteriovenous difference more positive (see e.g. [56]).

The relative rates of introduction of lactic acid and HCO_3_^−^ into ISF and CSF under normal conditions have not been accurately determined and furthermore little is known about the fractions of lactate removed to blood (regardless of route) as lactic acid or lactate^−^. Ball et al. [57] estimated that when [lactate] was elevated in spreading depression about half was removed by efflux directly to blood, i.e. across the blood–brain barrier presumably in the form of lactic acid by MCT1, and about half was effluxed indirectly, presumably as lactate^−^, via perivascular spaces. Similar proportions were seen in earlier work from the same group [433]. Clear, independent evidence that some of the lactate reaches the lymphatics has been obtained by assaying the lactate content of the cervical lymph nodes. Furthermore the proportion removed by this route was shown to be altered by many of the same factors that alter removal of inulin, which is thought to be removed via the perivascular spaces [434].

Studies on the production of lactic acid and removal of lactate have not yielded a clear answer for how much of the generated H^+^ must be removed by reaction with HCO_3_^−^.^26^ The evidence available indicates that the H^+^ from lactic acid is important when pCO_2_ is reduced below ca. 20 mmHg and in severe hypoxia (for discussion see Siesjö [417] and Mines and Sørensen [419]. However as shown by Pappenheimer, Fencl and colleagues [351, 352] (see Fig. 23) in metabolic acidosis and alkalosis the changes in [HCO_3_^−^]_CSF_ appear to be closely matched by changes in the opposite direction of [Cl^−^]_CSF_. Their data were obtained for a wide range of [HCO_3_^−^]_arterial_ and for pCO_2_ between 20 and 45 mmHg [352]. These results leave little if any room for changes in [lactate^−^] and hence for lactic acid production to have significant effects on steady-state [HCO_3_^−^] or pH in CSF and ISF. The changes in [Cl^−^]_CSF_ and [HCO_3_^−^]_CSF_ reflect altered transport at the blood–brain barrier, the choroid plexuses or both. Events at the barriers can, of course, be affected by changes in electrical potential differences, and the concentrations of all ions, including [lactate^−^], will affect these potentials.

### Involvement of HCO_3_^−^ transport across the blood–brain interfaces in ISF and CSF pH regulation

Transport of HCO_3_^−^ across the blood–brain interfaces and its removal by outflow of CSF and ISF are the dominant processes determining [HCO_3_^−^] in CSF and ISF in the long term. As described in Sects. 3 and 4 transcellular transport of HCO_3_^−^ across both the choroid plexuses and the blood–brain barrier is directly coupled to the transport of other ions, primarily Na^+^ and Cl^−^.

Many of the early publications considered how the overall potential difference between CSF and blood (the PD) varies with plasma pH and the contribution this might make to regulation of pH in CSF. Held, Fencl and Pappenheimer [286] showed that the PD in goats and dogs became increasingly positive as [H^+^] in the blood increased (see below). Their striking findings, confirmed in several other species (see [185, 388, 417]), invited the hypothesis that in acidosis increased PD, which would tend to increase net flux of HCO_3_^−^ into CSF, accounts for an increase in the ratio [HCO_3_^−^]_CSF_/[HCO_3_^−^]_plasma_. This change would explain at least part of why the variation in pH in CSF is smaller than that in blood in metabolic acidosis and alkalosis. Certain aspects of these studies, including the evidence that the PD is produced by processes at the blood–brain barrier, are considered in footnote 27.

There are two persuasive arguments against this “simple” mechanism as the principal explanation of pH regulation. Firstly a variation of the PD in the opposite direction to that seen in most species can occur in cats even though they still regulate pH_CSF_ [189, 435, 436] (see pg 120 in [185]). Secondly by varying [K^+^]_plasma_ the PD can be changed by as much as 9 mV with no effect on the distribution of Na^+^, H^+^ or Cl^−^ [437–439]. This indicates that changes in the PD do not produce the changes in [HCO_3_^−^]_CSF_ and [H^+^]_CSF_ needed for regulation.

#### HCO_3_^−^ transport across the choroid plexuses

The importance of the choroid plexuses compared to the blood–brain barrier in determining [HCO_3_^−^] and hence pH within the brain extracellular fluids is not known with certainty. The choroid plexuses do determine pH in the CSF immediately adjacent to them. Furthermore at constant plasma pCO_2_ even at distant locations changes in [HCO_3_^−^]_CSF_ following changes in [HCO_3_^−^]_plasma_ occur on the same timescale of hours as the turnover of CSF. This means that choroid plexus secretion could be important in determining the final composition. However, the choroid plexuses cannot account for any rapid changes far removed from the ventricles. Furthermore there are at least three lines of evidence suggesting that transport at the choroid plexuses is less important than at the blood–brain barrier. Firstly, [K^+^]_CSF_ and [HCO_3_^−^]_CSF_ change as CSF flows from the ventricles to the cisterna magna [141, 244, 440]. Secondly changes in ventilation ascribed to changes in ISF pH at constant pCO_2_, i.e. those resulting from changes in [HCO_3_^−^]_ISF_, are faster than changes in [HCO_3_^−^]_CSF_ (see next section for further discussion) and electrode measurements of pH_ISF_ (see next section) indicate that this changes more rapidly than does pH_CSF_. Finally experiments collecting fluid directly adjacent to the choroid plexuses [440] have found little change in [HCO_3_^−^] in the secretion when [HCO_3_^−^]_plasma_ was altered at constant pCO_2_ (for further discussion see 28) indicating that the changes in [HCO_3_^−^]_CSF_ sampled at a distance from the choroid plexuses could not be due to alterations in choroid plexus secretion.

By contrast to the situation at constant plasma pCO_2_, results obtained when plasma pCO_2_ was altered showed that [HCO_3_^−^] in the choroid plexus secretion did change and in the same direction as pCO_2_ [141, 440] see also p. 282 in [16]. No satisfactory explanation has been given for how [HCO_3_^−^] in the secretion can depend on plasma pCO_2_ but not on [HCO_3_^−^]_plasma_. The mechanisms discussed in Sect. 3 suggest that increases in either pCO_2_ or [HCO_3_^−^] in plasma should increase the concentration of [HCO_3_^−^] in the choroid epithelial cells and hence in the secretion. These results and their interpretation are considered further in footnote 28.

#### HCO_3_^−^ transport across the blood–brain barrier

There is near universal agreement that pCO_2_ throughout the brain rapidly reflects changes in pCO_2_ in blood because CO_2_ crosses the blood–brain barrier rapidly (see Sect. 2.2). By contrast the evidence concerning transport of HCO_3_^−^ has been controversial with some studies supporting rapid, extensive transport (much faster than found for other ions like Na^+^, K^+^ and Cl^−^ using radiotracers though of course still much slower than for CO_2_) while others have indicated slow, perhaps even no, transport (see summary of evidence in Table 3). The presence at the blood–brain barrier of multiple HCO_3_^−^ transporters (see Sects. 4.4.2 and 4.5.2) renders it most unlikely that there is no HCO_3_^−^ transport and the evidence discussed below strongly supports the idea that such transport occurs at a rate comparable to that of Cl^−^.

**Table 3:** Summary of evidence that has been used to support or oppose the idea of rapid transport of HCO_3_^−^ following changes in [HCO_3_^−^]_plasma_ at constant pCO_2_

For	Against
High rate of HCO_3_^−^ loss from or gain to fluids perfused through the ventricles	Little or no change in total CO_2_ (almost all of which is HCO_3_^−^) in the brain in response to altered [HCO_3_^−^] in plasma
Rapid, easily seen changes in pH (measured by electrodes applied to the brain surface or microelectrodes within the parenchyma) in response to changes in [HCO_3_^−^]_plasma_	Little or no change in pH (measured by electrodes applied to the brain surface or microelectrodes within the parenchyma) in response to changes in [HCO_3_^−^]_plasma_
Measurable first-pass extraction of H^11^CO_3_^−^ from blood	No change in intracellular pH measured using ^31^P-NMR over an hour following decrease in plasma [HCO_3_^−^]
Acute changes in ventilation rate (after denervation of peripheral chemoreceptors) when [HCO_3_^−^]_plasma_ is changed at constant pCO_2_	low measured permeability to Cl^−^ determined using radiotracers

The pieces of evidence are listed in Table 3. Four of these have been used to support the view that there is rapid transport of HCO_3_^−^ across the blood–brain barrier.

(1) The first argument was put forward in the landmark papers on pH regulation and the central control of ventilation by Pappenheimer, Fencl and colleagues [351, 352] (see Figs. 21, 23). They used ventriculocisternal perfusion to look at the loss from or gain into the perfusion fluids of HCO_3_^−^ and Cl^−^. If the perfusion fluid had the same concentrations as in a sample of CSF withdrawn shortly before the perfusion, there was no net loss or gain of either ion. When a concentration in the perfusion fluid was higher there was rapid loss, while if a concentration was less there was rapid gain. From the concentrations with neither loss nor gain, they inferred that in the steady-state [HCO_3_^−^] and [Cl^−^] are the same in ISF and CSF, but different from those in plasma. For other concentrations the initial rates of loss or gain represent exchange with the parenchyma. However, they reasoned that after 45 min of perfusion, the concentrations in the parenchyma would have stabilized and thus that at that time the continuing loss or gain of HCO_3_^−^ and Cl^−^ from the perfusion fluid had to be equal to their transfers across the blood–brain barrier. However, they did not demonstrate that the rates of loss or gain had reached constant values. It has been shown using radiotracers for K^+^ and Na^+^ that 45 min is not long enough [251, 441] and thus that the measured rates of loss from or gain into the perfusate of Cl^−^ and HCO_3_^−^ are likely to have exceeded substantially their rates of transfer across the blood–brain barrier. Thus at present this line of evidence must be discounted. This matter is considered semi-quantitatively in 29.

(2) Another type of evidence put forward in support of rapid transport of HCO_3_^−^ across the blood–brain barrier was obtained using pH electrodes applied to the surface of the exposed cortex or pH microelectrodes below the cortical surface. These values were taken to represent changes in pH_ISF_. When [HCO_3_^−^]_plasma_ was altered at nearly constant pCO_2_, Ahmad and Loeschcke [442], Teppema and coworkers [443, 444] and Davies and Nolan [445] all found rapid changes (e.g. within 30 s) in pH_ISF_ and in one case also [Cl^−^]_ISF_ [442]. The inferred changes in [HCO_3_^–^]_ISF_ were large, major fractions of the change imposed in plasma. The discrepancies between these results and those discussed as item 2) of evidence against have still not been explained (see 30).

(3) Normally it is not possible to vary pCO_2_ and [HCO_3_^−^] independently of each other in blood because there is rapid interconversion between H^+^ + HCO_3_^−^ and CO_2_ (see Sect. 6.1.1). However, after total inhibition of carbonic anhydrase, it is possible to have disequilibrium concentrations of CO_2_ and HCO_3_^−^ for tens of seconds and this strategy has been used by Johnson et al. [51] to investigate the penetration of H^11^CO_3_^−^ and ^11^CO_2_ into the brains of dogs. They injected total ^11^CO_2_ (^11^CO_2_ + H^11^CO_3_^−^) in acid or alkaline solution into the aorta followed by very rapid recording of ^11^C in the head using positron emission scanning. The signal rose to a maximum within a few seconds as the bolus dose reached the head. After a further 10–15 s most of the signal left in the head was tracer that had crossed the blood–brain barrier. They found, as expected, that without carbonic anhydrase inhibition, the different injections all led to the same very rapid penetration, with extraction of more than 80% of the total ^11^CO_2_ in the bolus in a single pass. After carbonic anhydrase inhibition using acetazolamide, penetration was reduced and the reduction was much more marked when the total ^11^CO_2_ was injected in alkaline rather than acid solution, i.e. primarily as H^11^CO_3_^−^ rather than ^11^CO_2_ indicating as expected that CO_2_ can penetrate rapidly. However, the rate of penetration when most of total ^11^CO_2_ was in the form of H^11^CO_3_^−^ was greater than could be accounted for by movement of the small proportion of ^11^CO_2_ calculated to be present. They concluded from these results that H^11^CO_3_^−^ could penetrate rapidly enough to extract about 16% of that arriving in the blood. Their calculated permeability for H^11^CO_3_^−^ was about 30 times larger than the permeability for ^36^Cl^−^ [261]. Johnson et al. suggested that such large fluxes could not represent a net flux of HCO_3_^−^ and thus would have to involve some form of exchange.

There may be alternative explanations for the above results that do not invoke rapid movement of HCO_3_^−^. Firstly there may not have been total inhibition of carbonic anhydrase in which case the conversion of H^11^CO_3_^−^ to ^11^CO_2_ in the alkaline injection experiment would have been more extensive than calculated and the higher [^11^CO_2_] might then explain the observed penetration. It would have been reassuring had data been presented showing that higher concentrations of acetazolamide did not produce further inhibition. Secondly the results could be explained without penetration of H^11^CO_3_^−^ if there were a high permeability to H_2_CO_3_. At equilibrium H_2_CO_3_ is at a concentration about 400 times less than [CO_2_] [446] but during the disequilibrium period it could be present at a higher concentration. Apparently nothing is known about the membrane permeability of H_2_CO_3_. Fluxes of H_2_CO_3_ do not carry charge and with functional carbonic anhydrase would be very difficult to distinguish from the larger fluxes of CO_2_.

(4) Indirect evidence that HCO_3_^−^ (or, of course, H^+^) can cross the blood–brain barrier rapidly comes from studies following changes in ventilation when [HCO_3_^−^]_plasma_ is altered. It is widely believed that ventilation rate is determined by signals from both peripheral and central chemoreceptors and that the central chemoreceptors respond to pH (see e.g. pg 239 in [447]). In animals whose peripheral chemoreceptors have been denervated, additions of acid to plasma produced rapid [448] or acute [449] increases in ventilation without measurable changes in CSF pH. The changes could not be explained as responses to changes in pCO_2_ in plasma and it was concluded that “central” chemoreceptors can respond quickly or acutely to changes in pH and/or [HCO_3_^−^] in plasma. One interpretation of this result is that the central receptors are exposed to ISF and that pH and [HCO_3_^−^] in ISF change more rapidly than in CSF. However, it is not clear how much change in pH_ISF_ is required or even if there need be any rapid change if some of the central chemoreceptors are in regions not protected by the blood–brain barrier and so can monitor changes in [HCO_3_–]_plasma_ directly (see e.g. pp. 235–6 and 244 in [447]).

At least four lines of evidence exist that support the view that transport of HCO_3_^−^ across the blood–brain barrier is either slow or non-existent.

(1) Measuring total CO_2_ (most of which is HCO_3_^−^) in brain tissue, Siesjö and colleagues [450–452] found no change after 6 h exposure to high or low HCO_3_^−^ in plasma. However, while these results exclude fractional changes in [HCO_3_^−^] in the parenchyma as large as those in plasma, the sensitivity of the measurements was not sufficient for the results to argue against the existence of the smaller changes considered in points (ii)–(iv) below.^31^ Small changes in [HCO_3_^−^]_CSF_ are known to occur, see Fig. 21.

(2) A number of studies using either pH electrodes applied to the cortical surface or pH microelectrodes inserted into the cortex support slow penetration of the blood–brain barrier by HCO_3_^−^. In the first study using pH electrodes placed against the surface of the cortex, Rapoport [453] (see also [454]) found that intravenous injection of HCl or NaOH led to the expected decrease or increase in pH_plasma_ and produced transient pH changes at the cortical surface in the same direction as in plasma. By contrast injection of NaHCO_3_ leading to the expected alkaline shift in plasma produced a clear, transient acid shift at the cortical surface. These are the expected results if the blood–brain barrier is intact and CO_2_ can cross this barrier much more rapidly than HCO_3_^−^. In brief, injections of NaHCO_3_ or of HCl by reaction with HCO_3_^−^ will increase pCO_2_ in the blood while those of NaOH by reaction with CO_2_ will decrease it and these changes in CO_2_ will produce changes in pH_ISF_. Such changes will be transient because the excess or deficit of CO_2_ will be offset by distribution of CO_2_ and HCO_3_^−^ throughout the body and by ventilation. Javaheri et al. [455] have called these results “a positive Rapoport test”. Rapoport’s results demonstrate that CO_2_ crosses the blood–brain barrier much more rapidly than HCO_3_^−^ but they do not exclude slow HCO_3_^−^penetration that would only be apparent on time scales longer than the 10 min he used.

Another pH electrode study, this time using microelectrodes inserted into the parenchyma to study pH_ISF_ [456] found definite but slow penetration of HCO_3_^−^ across the blood–brain barrier. In this study the transient acidification of ISF described by Rapoport following addition of NaHCO_3_ to blood was not detected, possibly because of the limitations of using high impedance electrodes to measure small changes in pH in an environment with both electrical and mechanical “noise”.

A further study by Javaheri et al. [455] used pH electrodes on the cortical surface with pCO_2_ held constant to exclude changes in pCO_2_ as a factor changing pH_ISF_. They found that intravenous infusions of acid or base for 30 min produced significant shifts in the surface pH in the same direction as in plasma. Much smaller changes were noted in pH_CSF_. To check that the blood–brain barrier was intact, before the infusion in each experiment they used the Rapoport test as described above. In a subsequent study using pH microelectrodes with tips located 5 mm below the cortical surface, Javaheri et al. [457] found smaller but significant changes in pH_ISF_ that were still increasing at the end of an hour. These were much larger than the changes, if any, over the same period in CSF, but much smaller than those in plasma.

(3) Using ^31^P-NMR to monitor intracellular pH within the brain Adler et al. [458] found no change over an hour when pH_plasma_ was reduced by 0.25 units. They noted that they would not have been able to see changes corresponding to those reported by Javaheri et al. (see just above). By contrast it is very difficult to reconcile this result with the large, rapid change in pH_ISF_ reported by Ahmad and Loeschcke [442].

(4) A strong argument against large rapid changes in [HCO_3_^−^]_ISF_ in response to changes in [HCO_3_^−^]_plasma_ is that these must be accompanied by changes in the opposite direction in [Cl^−^]_ISF_ (see Sect. 6.1). The permeability of the blood–brain barrier to Cl^−^ has been measured using radiotracers [269] and is much too small to support the rapid changes reported by Ahmad and Loeschcke [442] (see also footnote 30) and Johnson et al. [51].

To summarize, the balance of evidence from the in vivo results suggests that there is slow transfer of HCO_3_^−^ across the blood–brain barrier, presumably at rates of the same order as for Cl^−^. Despite extensive research on the regulation of pH_ISF_ carried out between 1950 and 1998 (see [185, 393, 394]), some of which has been discussed above, there is still uncertainty about the changes in the rate of HCO_3_^−^ and Cl^−^ transport across the blood–brain barrier in situ when [HCO_3_^−^]_plasma_ is changed. With brain endothelial cells in culture, transport of HCO_3_^−^ can occur by multiple transporters. These could support a net transport adequate for fluid secretion as large as 200 ml day^−1^ (see Sect. 4.1.1) and could account for the estimates of HCO_3_^−^ transport reported by Javaheri and coworkers [455, 457] from their in vivo studies on pH regulation.

In contrast to responses to changes in [HCO_3_^−^]_plasma_, when pCO_2_ is altered there are rapid and sustained changes in [HCO_3_^−^]_ISF_ [16, 185]. Initially a substantial part of the change may result from physiological buffering but in the long term the changes result from altered transfer of HCO_3_^−^ across the blood–brain interfaces with that across the blood–brain barrier likely to be more important. In both schemes in Figs. 17 and 18 depicting mechanisms for transport across the blood–brain barrier, the rate of HCO_3_^−^ transport would increase with increases in either pCO_2_ or [HCO_3_^−^] in plasma. By contrast the fluid secretion rate across the blood–brain barrier is predicted to be relatively insensitive to these changes (at least at constant pH) because the principal transporters for Na^+^ and Cl^−^ do not depend on the presence of CO_2_ or HCO_3_^−^. The present state of knowledge of transport mechanisms at the blood–brain barrier is not sufficient to predict the sizes of changes in [HCO_3_^−^]_ISF_ relative to those in plasma.

## Summary

The two blood–brain interfaces, the blood–brain barrier and the choroid plexuses, fulfil very different roles.

These are reflected in their differing properties and locations in the brain as itemized and summarized in Table 4.

**Table 4:** Comparison of properties of the blood–brain barrier and the choroid plexuses

Topic	Blood–brain barrier	Section or ref.	Choroid plexus	Section or ref.
Principal roles	Exclusion of unwanted substances, retention of required substances, rapid transfers of O_2_, CO_2_ and regulated transfers of metabolites and wastes	1	Secretion of CSF excluding unwanted substances	1
Nature of barrier				
Location	Wall of microvasculature distributed throughout brain and spinal cord		Discrete entities one protruding into each ventricle	
Cell types contributing to barrier function	Endothelial cells exposed directly to blood but surrounded on parenchymal side by basement membrane, pericytes and astrocyte endfeet	1, 4, 5	Epithelial cells exposed directly to CSF. Adjacent stroma and capillary wall provide only a small transport barrier	1, 3
Connections between cells	High resistance tight junctions containing claudin 5 between endothelial cells that greatly restrict paracellular transport	4.34	Low resistance “leaky” tight junctions containing claudin 2 between epithelial cells; underlying peripheral-type, leaky blood vessels	3.4.2, 3.6.4
Surface area	Various estimates, 50–240 cm^2^ g^−1^. Smith and Rapoport [261] quote 140 cm^2^ g^−1^	[261, 531–533]	Similar to area of blood–brain barrier. Highly folded at both subcellular and cellular levels to fit into the ventricles	[1, 2]
Ratio of cell membrane area to length of tight junction band around the cell	Relatively small compared to choroid plexuses. Cell surfaces are not folded	4.4	Relatively large compared to blood–brain barrier. Apical brush border and highly folded basolateral membrane	4.4
Blood flow	0.54 ml g^−1^ min^−1^ (but variable, see below)	[534]	Three to ten fold larger than for blood-flow at the blood–brain barrier	2
Percentage of cerebral blood flow	~99%	2	~1%	2
Barrier type	High resistance and low permeability to highly polar substances (e.g. mannitol, sucrose). Permeability increases with lipid solubility		Classic leaky epithelium producing a high volume of nearly isosmotic fluid transport	
Transfers related to metabolism				
O_2_ and CO_2_ transfers	Very rapid. At least partly blood-flow limited. Neurovascular coupling to ensure adequate blood flow to regions with high metabolism	2.3	Presumed to be rapid. Total transfers to and from brain much less than across blood–brain barrier because much less blood flow	2.3
Glucose transfer	Rapid transport across barrier, extraction of ~30% of that arriving in blood. Passive via GLUT1 in luminal and abluminal membranes. *K*_*m*_ close to normal [glucose]_plasma_ which limits excessive entry during raised [glucose]_plasma_	2.4.1 and see also footnote 1	Total amount transferred small compared to that across the blood–brain barrier. GLUT 1 transporters present in basolateral membrane, but far fewer in apical membrane	2.5
Lactic acid transfer	Rapid, but saturable, passive transport via MCT1 in luminal and abluminal membranes	6, 6.3	Existence of transepithelial transport unclear. MCT3 present but only in the basolateral membrane.	[535]
Amino acid transfers	Selective transport via multiple transporters. Concentration in ISF ~1/10th that in plasma. Passive transporters on both sides and Na^+^-linked transporters on abluminal side maintain this gradient. Amounts transported imply that the Na^+^ transfer via this route likely to exceed the net transfer of Na^+^ in any fluid secretion	2.4.2	Same types of transporters as at blood–brain barrier with Na^+^-linked transporters in the apical membrane, but total amount of amino acids transferred much less than at the blood-brain barrier	2.5
Vitamins and “micronutrients”, e.g. folate	Minor route of transport	[2, 137]	Major route of transport into brain	3.1, [2, 137]
Transfers related to fluid secretion and regulation of [K^+^] and [HCO_3_^−^]				
Fluid secretion rate	Unknown. On present knowledge could be anywhere between small net absorption and net secretion comparable to ~50% of CSF production by choroid plexuses. Ion transporters needed are present, so net movement unlikely to be zero. If secretion rate is large, a large portion of ISF must be returned to blood without first mixing with CSF in the cisterna magna	4.1	350–400 ml day^−1^	3.2
Water permeability	Sufficient to allow a close approach to osmotic equilibrium between brain and blood. Most water from blood crosses the barrier in a single-pass through microvessels, some via the lipid bilayers of the cell membranes, some possibly via membrane proteins, e.g. GLUT1, some possibly paracellular. Aquaporins absent in endothelium, but AQP4 present on astrocyte endfeet	2.1, 4.3.6	High. Some via AQP1 in apical membrane of epithelium and possibly GLUT1 in basolateral membrane. Some via paracellular route	2.1, 3.4.2
Na^+^ transporters	Abluminal: Na^+^, K^+^-ATPase (most), Na^+^-linked transporters, e.g. for amino acids, NHE (some) and possibly NBCe1. Luminal: NKCC1, NBCn1, NDCBE-like, Na^+^, K^+^-ATPase (some), NHE (most) and possibly NBCe1	4.6.1 and Figs. 17 and 18	Apical: Na^+^, K^+^-ATPase, NKCC1, NBCe2, NHE1 (nearly silent at normal pH?). Basolateral: NCBE/NBCn2, (NBCn1?)	3.6.1, 3.6.11 and Fig. 6, [4]
Na^+^ tracer influx, blood towards brain	~3.4 × 10^−5^ cm^3^ s^−1^×[Na^+^]_plasma_ in rats, possibly mainly paracellular	4.3.2 and 4.3.4	~3.8 × 10^−5^ cm^3^ s^−1^×[Na^+^]_plasma_ in rats	4.3.2
Na^+^ tracer efflux, brain towards blood	Similar to rate of influx	Footnote 13, 4.3.2, 4.3.5	Smaller than influx	3.6.1 and footnote 6
Na^+^ net flux	Unknown, see entry Fluid secretion rate		~ 0.15 mol l^−1^ × 400 ml day^−1^ = ~0.69 µmol s^−1^	
Cl^−^ transporters	Luminal: NKCC1, NDCBE-like? Unknown sidedness: AE2, anion channels	4.4.2, 4.6.2	Apical: KCC4, NKCC1 and probably anion channels. Basolateral: AE2 and KCC3.	Figure 5; 3.6.2
K^+^ transporters	Abluminal: Na^+^, K^+^-ATPase (most) Luminal: NKCC1 and Na^+^, K^+^-ATPase (some) Both: K^+^ channels	4.4.1 and 4.5.3	Apical: Na^+^, K^+^-ATPase, KCC4, NKCC1 and K^+^ channels. Basolateral: KCC3	3.6.4
Contribution to [K^+^]_ISF_ regulation	Major	4.3.1, 5, 5.2, 5.3	Minor	3.6.4, 5.1
K^+^ fluxes, trans- vs. para-cellular	Amount transported substantially greater than at choroid plexuses. Mainly transcellular	4.3.1, 4.3.4 and 5.2	Transcellular net efflux from CSF and paracellular net influx into CSF	3.64
K^+^ tracer influx to brain, acute changes in [K^+^]_plasma_	Increased with increased [K^+^]_plasma_	5.2	Increased with increased [K^+^]_plasma_	5.1
K^+^ tracer influx to brain, chronic changes in [K^+^]_plasma_	Influx independent of [K^+^]_plasma_. Downregulation of luminal transporters with increased [K^+^]_plasma_, possibly NKCC1? Upregulation of luminal K^+^ channels?	5.2, [361]	Influx not increased by increasing [K^+^]_plasma_. Unknown mechanism	5.1, [361]
K^+^ tracer efflux from brain	Rate varies sigmoidally with [K^+^]_ISF_, transport via Na^+^, K^+^-ATPase in abluminal membrane	4.3.1	Presumably sigmoidal variation as at blood–brain barrier	
HCO_3_^−^ transporters	NBCe1, NBCn1, AE2, NDCBE-like?, localization not known	4.4.2	Apical: NBCe2 Basolateral: AE2, NCBE/NBCn2	Figures 5 and 6, 3.6.2
Rate of HCO_3_^−^ transport	Controversial, fast vs. slow. Balance of evidence now strongly favours slow	6.4.2	~25 mM × 400 ml min^−1^ = 10 mmol min^−1^	6.4.1
Modulation of HCO_3_^−^ transport by pCO_2_	HCO_3_^−^ influx may be increased by increases in [HCO_3_^−^]_plasma_ or pCO_2_. Increased [HCO_3_^−^]_plasma_ leads to increased pH_ISF_ in advance of changes in pH_CSF_	6.4.2	[HCO_3_^−^] in the secretion changes in the same direction as pCO_2_	6.4.1
Importance for regulation of pH_ISF_	Major via regulation of [HCO_3_^−^]_ISF_	6.4	Unclear	6.4

Numerical values are scaled to be appropriate for an adult human with a 1.4 kg brain unless stated otherwise

## Conclusions

The primary roles of the blood–brain barrier are to restrict entry of unwanted substances circulating in the blood, prevent loss of required substances and at the same time provide the means for rapid transfers of O_2_, CO_2_ and glucose to support the metabolic needs of cells in the brain (Sect. 2). In this way it is able to control the composition of the interstitial fluid surrounding the brain cells thus allowing them to function effectively. Large fractions of the water, O_2_, CO_2_ and glucose that arrive in the cerebral blood flow are transferred into the brain in a single pass and there are comparable fluxes in the opposite direction (see Sect. 2). The choroid plexuses cannot replace the principal actions of the blood–brain barrier for these functions. The primary role of the choroid plexuses is to secrete fluid.

The total rate of fluid secretion into the brain is approximately 0.1% of cerebral blood flow. Most of this secretion is by the choroid plexuses that receive about 1% of cerebral blood flow. The remaining 99% of the blood flow is separated from the brain by the blood–brain barrier. The rate of net fluid transport across the blood–brain barrier is still unknown.

The water permeability of the blood–brain barrier is much lower than that of peripheral capillaries but still sufficiently large that even very small osmotic gradients could drive net water movement into the brain. However, the permeability to small ions is low which greatly reduces any possible hydrostatic pressure driven flow. Instead fluid transfer across the blood–brain barrier requires transport of solutes, particularly NaCl, with the water following osmotically.

From knowledge of the identities and locations of the transporters responsible for ion transport across the epithelial cells of the choroid plexuses, a description of events driving fluid transport can be constructed. Of note: Na^+^ is transported across the apical (CSF facing) membrane of the epithelial cells primarily by the Na^+^ pump and across the basolateral (stroma and capillary facing) membrane by cotransport with HCO_3_^−^. There is presumably a paracellular back-leak of Na^+^. Cl^−^ is transported across the basolateral membrane by Cl^−^/HCO_3_^−^ exchange and across the apical membrane by multiple mechanisms. Transcellular transport of K^+^ is in the direction from CSF to blood because the only means for its transport across the basolateral membrane is K^+^, Cl^−^-cotransport, which mediates a net efflux from the epithelial cells. However net K^+^ secretion is from blood to CSF across the choroid plexuses, so it follows that paracellular transport of K^+^ from blood to CSF must exceed the transcellular transport. This description of choroid plexus fluid secretion does not explain all of the available evidence. It fails to explain the relative independence of both [HCO_3_^−^] in the secretion and CSF secretion rate from changes in [HCO_3_^−^]_plasma_ (see Sect. 6.4.1 and footnote 28). Furthermore it has not been established that the mechanisms for HCO_3_^−^ transport require conversions between CO_2_ and HCO_3_^−^. If they do not, this description provides no explanation for the inhibition of secretion by the carbonic anhydrase inhibitor, acetazolamide (see Sect. 3.6.3).

More than one comparable description of events can be constructed for fluid transfer across the blood–brain barrier. The endothelial cells lining the brain microvessels have a complement of ion transporters that if appropriately arranged would allow active secretion of fluid. The Na^+^-pumps that are present are capable of expelling substantially more Na^+^ from the cells than would be needed for secretion. However, much is still unknown: exactly how the transporters are distributed between the luminal and abluminal membranes, the extent of the net transfers of Na^+^ and Cl^−^ across the barrier, and the rate of fluid secretion. There is strong but still inconclusive in vivo evidence that the blood–brain barrier does secrete fluid (see Sect. 4.1).

K^+^ enters the endothelial cells of the blood–brain barrier via Na^+^, K^+^-ATPase located primarily in the abluminal (brain side) membrane and via Na^+^, K^+^, 2Cl^−^-cotransporters across the luminal (blood side) membrane. Efflux from the cells is thought to be primarily via different K^+^ channels in the membranes. Small changes in any of these components of K^+^ transport may produce relatively large changes in net flux, which could be from brain to blood or blood to brain as required.

HCO_3_^−^ enters the endothelial cells primarily by Na^+^, HCO_3_^−^ cotransport and leaves the cells either by Cl^−^/HCO_3_^−^ exchange or by Na^+^, HCO_3_^−^ cotransport in 1 Na^+^, 3 HCO_3_^−^ mode.

Na^+^ and Cl^−^ entry to the brain across the blood–brain barrier is similar to or somewhat greater than Na^+^ and Cl^−^ entry across the choroid plexuses. However, at the blood–brain barrier almost the same amount leaves as enters the brain and the relatively small net transfers have proved difficult to measure. By contrast at the choroid plexuses less comes out of CSF than goes in and the net fluxes represent a larger proportion of the influxes.

The role of astrocyte endfeet in the function of the blood–brain barrier is still poorly understood. It is likely that much of the glucose that crosses the blood–brain barrier enters the endfeet. AQP4 in endfeet membranes may be there to allow water produced during glucose oxidation to enter the basement membrane separating endfeet from endothelial cells. There it would either dilute hyperosmotic secretion across the blood–brain barrier or be part of a hypoosmotic absorbate. The function served by the high density of K^+^ channels in the endfeet is still unknown but there are speculative suggestions (Sect. 5).

Regulation of [K^+^]_ISF_ appears to occur primarily by control of influx and efflux across the blood–brain barrier. K^+^ efflux from the brain increases markedly for small increases in [K^+^]_ISF_, but there is little change in K^+^ influx in response to sustained changes in [K^+^]_plasma-_ presumably because there is a reduction in either the number or activity of K^+^ transporters or channels (Sect. 5).

The determinants of pH_ISF_ are pCO_2_ and [HCO_3_^−^]_ISF_: pCO_2_ is in turn determined primarily by the metabolic and ventilation rates, with a relatively constant difference in value between pCO_2_ in arterial blood and ISF. The available evidence indicates that [HCO_3_^−^]_ISF_ is determined primarily by transport across the blood–brain barrier. While some evidence has suggested very rapid transfers of HCO_3_^−^, the balance of evidence strongly favours slow transport with rates similar to those for other ions. However, this slow transport is still adequate for the blood–brain barrier to be the main site of [HCO_3_^−^]_ISF_ regulation.

Neither the blood–brain barrier nor the choroid plexuses provide the major route for net fluid outflow which occurs by pressure driven fluid flow by a variety of routes, including the arachnoid villi, meningeal lymphatics, nerve tracts through the cribriform plate, perineural pathways in the spinal cord and probably also other perineural and perivascular pathways (see Figs. 8 and 9).

Although the blood–brain barrier is vital for effective brain function, it is less clear that the choroid plexuses are needed in the adult. Thus while secretion by the choroid plexuses does account for most of the net fluid entry into the brain, a suitable arrangement of transporters at the blood–brain barrier could easily produce a similar secretion. One must look for somewhat more subtle reasons for having choroid plexuses as the major source of CSF. Firstly it may allow the blood–brain barrier to serve local needs free from constraints of having to maintain fluid secretion, e.g. in response to changes in nervous activity. Secondly, it avoids the need to provide pathways and sufficient pressure gradients for flow through the parenchyma of all the fluid entering the brain. Thirdly fluid secretion directly into the ventricles may help to maintain their patency. Having such spaces of variable volume with little resistance to flow between them allows compensation for changes in blood volume during the cardiac and respiratory cycles and in response to changes in posture. It should also be noted that the choroid plexuses are needed to secrete fluid during CNS development and it may be an economical solution to allow them to continue to do so in the adult. Finally it may be efficient for the choroid plexuses to have a role in hormonal signalling. Because the choroid plexuses are accessible for studies of transport and importantly they express ion transporters at sufficiently high levels to allow clear, convincing immunohistochemistry, it is not surprising that much more is known about the mechanisms of transport across them than across the blood–brain barrier.

More attention needs to be paid to the function of the blood–brain barrier, if volume, composition and flow changes in ISF occurring in disorders such as hydrocephalus and oedema following stroke are to be understood. The relative importance of blood–brain barrier fluid secretion and CSF recirculation through the parenchyma in the clearance of high molecular wastes is an area currently receiving a great deal of attention. Certainly there are many areas where experimental results are needed to extend our knowledge. These include: direct measurements of net fluxes across the blood–brain barrier; recordings of intracellular potentials during transport across the blood–brain barrier and the choroid plexuses under conditions close to those obtaining in vivo; localization of transporters at the blood–brain barrier to the luminal or abluminal membranes in particular the distribution of Na^+^-pump activity and whether this can be altered in response to different challenges; determination of clearance of solutes from brain parenchyma without barbiturate anaesthesia; clarifying whether outflow of ISF from the parenchyma is periarterial, perivenular, or both; obtaining quantitative measurements of fluid flows via perivascular pathways.

## Abbreviations

AE2: anion exchanger 2
AQP1: aquaporin 1
AQP4: aquaporin 4
CAII: carbonic anhydrase 2
CAIV: carbonic anhydrase 4
CMEC: (bovine) cortical microvascular endothelial cells
CSF: cerebrospinal fluid
DIDS: 4,4′-diisothiocyano-2,2′-stilbenedisulfonic acid
EIPA: ethylisopropylamiloride
ENaC: epithelial Na^+^ channel
GLUT1: glucose transporter 1
ISF: interstitial fluid
MCT1: monocarboxylate transporter 1
NBC: Na^+^, HCO_3_^−^-cotransporter
KCC3: K^+^, Cl^−^-cotransporter 3
KCC4: K^+^, Cl^−^-cotransporter 4
K-NPPase: K^+^ dependent *p*-nitrophenylphosphatase
NBCe1: electrogenic Na^+^, HCO_3_^−^-cotransporter 1
NBCe2: electrogenic Na^+^, HCO_3_^−^-cotransporter 2
NBCn1: neutral Na^+^, HCO_3_^−^-cotransporter 1
NBCn2: neutral Na^+^, HCO_3_^−^-cotransporter 2
NCBE: Cl^−^-dependent Na^+^, HCO_3_^−^-cotransporter
NDCBE: Na^+^ dependent Cl^−^/HCO_3_^−^-exchanger
NHE: Na^+^/H^+^ exchanger
NHE1: Na^+^/H^+^ exchanger 1
NHE2: Na^+^/H^+^ exchanger 2
NKCC1: Na^+^, K^+^, 2Cl^−^-cotransporter 1
NMDG^+^: *n*-methyl-d-glucosamine
PC-MRI: phase contrast magnetic resonance imaging
SRM/MRM: selected/multiple reaction monitoring experiments

## Footnotes

1
*Glial endfeet, hexokinase and glucose fluxes across the blood–brain barrier* Exchange of glucose across the blood–brain barrier between blood and astrocyte endfeet rather than between blood and interstitial fluid (see Sect. 2.4.1) can lead to larger efflux relative to influx. This is possible because the endfeet express the glucose transporter GLUT1, which allows fluxes in both directions [102, 104, 105]. Thus there could be a large unidirectional flux back out of the endfeet if there is a significant [glucose] within the astrocytes. That in turn would require a relatively slow rate of phosphorylation of glucose by hexokinase. This requirement is consistent with the view, said to originate with Betz [112], that net uptake of glucose into the brain is limited by its rate of phosphorylation by hexokinase.

2
*Balance between total rates of influx and efflux of amino acids* Felig [467] lists the differences in arterial and venous plasma concentrations for a number of amino acids in the cerebral circulation of human volunteers. The sum of these differences is 125 µmol l^−1^. If it is assumed that for a 1400 g brain, the plasma flow is 0.4 l min^−1^, this corresponds to a net uptake of 35 nmol min^–1^ g^−1^. However, Felig's list notably lacks entries for glutamine and glutamate for which, see Sect. 2.4.2, there are likely to be substantial net releases. Thus these data do not challenge the view that total influx of amino acids is nearly balanced by the total efflux. Betz and Gilboe [468] list net uptakes for a number of amino acids for perfused dog brains in the presence of methoxyflurane or halothane for which the total uptakes are 5 and −5 nmol min^−1^ g^−1^ neither of which is significantly different from zero. Thus these results are also consistent with total influx of amino acids being nearly balanced by total efflux. It is worth noting that the efflux to blood of glutamine and of glutamate and NH_4_^+^ produced from them in the endothelial cells is the major route for export of amino-group-derived nitrogen from the brain [120, 469].

3
*Location of Na* ^+^-*coupled amino acid transporters* Smith et al. [470] demonstrated using in situ brain perfusion protocols and a number of radiolabelled neutral amino acids, including glutamine that there was a saturable uptake of amino acids into the brain. Ennis et al. [122] extended the results for glutamine and demonstrated that the influx rate was reduced by 62% when the [Na^+^] in the perfusion fluid was reduced from 138 to 2.4 mM. Based on this dependence they proposed that roughly 60% of the transport across the luminal membrane was via system N, which is a Na^+^-dependent transporter. However, Lee et al. ([471], reviewed in [120, 472, 473]) found using fractionated membrane vesicles that the luminal membrane contained system n, a Na^+^-independent carrier, while system N was present only in the abluminal membrane. There is evidence from studies using hepatocytes that two such systems exist [474–478]. Furthermore it is now known that there are far more genes for amino acid transporters than were envisaged when the system N, A, L etc. nomenclature was developed [479]. One part of the explanation of the apparent discrepancy concerning the Na^+^ dependence of glutamine transport across the luminal membrane [122, 471] may be that the methods used by Hawkins and coworkers do not completely exclude mixed expression of the transporters on the two sides of the cells. Another part may arise because the unidirectional flux from perfusate into the brain is measured across a cell layer rather than a single membrane. In order for glutamine to cross from plasma or perfusate to the interstitial fluid of the brain, it must cross the luminal membrane, diffuse across the cell cytoplasm, cross the abluminal membrane and then diffuse across the basement membrane and outwards either through or around the astrocyte endfeet (compare Sect. 5). Only then does it count as part of the unidirectional influx into the brain. Transport across the luminal membrane will be primarily Na^+^-independent, system n, and hence bidirectional while that across the abluminal membrane will be primarily Na^+^-dependent, system N, and hence biased towards movement from basement membrane into the cell cytoplasm. When the perfusate has a normal [Na^+^], the [Na^+^] within the endothelial cell will be substantial (perhaps >30 mM, compare with values in Table 2) and system N will mediate some efflux of glutamine from the cell to the basement membrane (compare [480]), and in addition glutamine may leave the cell via another transporter, system L1, that is known to be present. Some of the glutamine reaching the basement membrane will be transported back into the cell via system N, the rest will escape into the interstitial fluid of the brain and be part of the measured unidirectional flux from perfusate to brain. When the [Na^+^] in the vascular perfusate is greatly reduced, that inside the endothelial cells will also be reduced and there will be a larger gradient of [Na^+^] between the basement membrane and the cell interior. This may decrease glutamine efflux from the cells into the basement membrane via system N and increase glutamine influx in the reverse direction. Both of these effects would reduce the measured unidirectional flux from perfusate to brain. Thus the finding that Na^+^ removal decreases the tracer flux of glutamine into the brain does not in itself prove that there is a Na^+^-dependent amino acid transporter for glutamine in the luminal membrane.

4
*Choroid plexus secretion and blood flow* Choroid plexus secretion rate as a percentage of blood flow to the plexuses can be calculated from estimates of the fraction of cerebral blood flow that goes to the plexuses, ~0.01 (see Sect. 2), the volume fraction of plasma in blood, ~0.5, the cerebral blood flow, ~800 ml min^−1^, and the rate of CSF production ~400 ml day^−1^ = ~0.3 ml min^−1^, with the result 0.3 ml min^−1^/(0.01 × 0.5× 800 ml min^−1^) = 7.5%.

5
*Solute and water gradients in kidney proximal tubule and choroid plexus* When solutes that cannot be absorbed are added to renal proximal tubular fluid in a segment that is blocked at both ends, the epithelial cells initially continue to transport NaCl and water out of the lumen to the blood. A steady-state with constant tubular contents is reached when passive NaCl leak back into the lumen balances active transport out. There is then a gradient of [NaCl], higher outside than inside, but no gradient of osmolality as the difference in osmolality for the impermeant solute balances that of NaCl. There must be active transport of NaCl to generate and maintain its gradient but there is no evidence from these experiments for active transport of water [149, 150]. Similar conclusions had been reached earlier using rat ileum [481], but the arguments used in the experiments on proximal tubules [149, 150] are particularly elegant in that they are based on the steady-state condition in which there are no net fluxes of solutes or water. In particular in this condition because there is no flow and no net transport of NaCl there are no gradients of concentration in any "unstirred layers" (see next paragraph). Comparable tests assessing the steady-state gradients that can be maintained in the absence of flow appear never to have been carried out for the choroid plexus. The closest to such a test is probably the demonstration by Heisey et al. [482] that the choroid plexus continues to secrete even when the fluid in the ventricles is diluted, a result similar to that seen in the gall bladder where absorption of fluid can proceed when the lumen is hyperosmotic by as much as 80 mOsmol kg^−1^) [483, 484]. However, as discussed by Spring [151] for the gall bladder, this type of observation is not sufficient to prove "active transport of water" [485–487]. For the choroid plexus when there is net flow it may be possible to have lower osmolality in the CSF than the blood but at the same time higher osmolality at the apical surface (CSF side) than at the basolateral surface (blood side) of the epithelial cells. The difference is made up by the osmotic gradients across "unstirred layers", presumably primarily between the capillaries and the epithelial cells. Thus water could be moving down the osmotic gradient created by active transport of solute across the epithelial layer while being carried through the non-selective unstirred layers by pressure driven bulk flow of the entire solution. No active transport of water per se would then be required to account for the net secretion. With epithelia uphill water movement has only been seen when there are supporting structures, e.g. connective tissue, that create conditions in which unstirred layers are inevitable [151].

6
*Tracer and net fluxes of Na* ^*+*^ *across the choroid plexuses* Davson and Segal [173] and Davson and Welch [264] found that the tracer Na^+^ flux towards the CSF in the rabbit was just sufficient to account for the amount of Na^+^ in the secretion leaving no room for a tracer flux in the opposite direction. This is a very surprising result because for a leaky epithelium there should be tracer fluxes in both directions and this has been observed in other studies. For instance the tracer fluxes have been shown to be several fold larger than the net fluxes for proximal tubules of the kidney [488–491] and the choroid plexuses of bullfrogs [492, 493]. Smith and Rapoport [261] and Murphy and Johanson [265] reported tracer Na^+^ flux towards CSF in the rat of 2.3% min^−1^ (relative to Na^+^ content of the CSF) but did not report net CSF secretion rates. Their values for the influx are about three times larger than needed to account for the secretion rates (expressed as a percentage of CSF volume) measured by others, using ventriculo-cisternal perfusion: 0.72 or 0.75% min^−1^ (values in Table 6.2 of [17] calculated from data in [173] and [251] respectively).

7
*Effects of acetazolamide on aquaporins* There is considerable interest in finding alternative actions of acetazolamide as its effects in treating acute mountain sickness have still not been adequately explained [494]. On the balance of present evidence, acetazolamide may inhibit water fluxes via the aquaporin AQP4 found in astrocytes. However, AQP4 is not found in the choroid plexus and acetazolamide appears not to inhibit AQP1 that is present [495]. Even for AQP4 acetazolamide only inhibits at concentrations approaching its solubility limit in water, ~1.5 mM [496] (but see also references in [494]).

8
*Osmolality of fluid transferred across the blood–brain barrier: effect of metabolic water production* Metabolic water production, ~3.3 mol day^−1^ (see Sect. 2.2) expressed in terms of volume is 3.3 mol day^−1^ × 18 ml mol^−1^ = 60 ml day^−1^. This is very small compared to the large amounts of water entering and leaving the brain but it is not small compared to the possible net water flow across the blood–brain barrier. As a consequence the osmolality of the secretion across the barrier, defined as amount of solutes secreted divided by amount of water secreted, may be surprisingly large. If the net rate of production of fluid by the combination of the blood–brain barrier and metabolic production of water were 200 ml day^−1^ and the metabolic production of water 60 ml day^−1^, then for each 140 ml of water transferred across the blood–brain barrier into ISF, 30 mmol of NaCl would have to be transferred for the final [NaCl] of ISF to be 150 mM. Thus the ratio of the net amount of NaCl transported across the blood–brain barrier to the net amount of water crossing the barrier would be 30 mmol/140 ml = 214 mM which is much greater than the concentration in either the endothelial cells or the ISF. The osmolality of the fluid within the basement membrane separating the endothelial cells from the astrocyte endfeet is considered in Sect. 4.7. The calculation above assumes that the osmolality of the net outflow of ISF from the parenchyma is determined almost entirely by [NaCl] as would be correct if the composition of ISF were the same as that of CSF (see e.g. Table 2.5 in [17]). However, metabolic wastes may be at higher concentrations in ISF than in CSF. It should be noted that the principal metabolites, e.g. CO_2_, amino acids etc., need not be considered as they are at low concentrations as a result of transport across the blood–brain barrier (see e.g. Sect. 2). Similarly as noted in Sect. 6.3, [lactate^−^]_ISF_ is normally only of the order of 1–2 mM. More generally for this example calculation to be valid, the rate of production of those osmotically active metabolic wastes that are removed from the parenchyma by outflow of ISF must be substantially less than 18 mOsmol day^−1^.

9
*Evidence obtained in studies on hydrocephalus concerning fluid secretion by the blood–brain barrier* (Item 3 in Sect. 4.1 is amplified in the following)

1. When the cerebral aqueduct is blocked CSF production within the lateral and third ventricles continues but the ventricles do not continue to swell at a rate that would accommodate the CSF added, i.e. there must then be a route of escape from the lateral or third ventricles. This route has been called "ventricular absorption" [220, 221]. The evidence establishing ventricular absorption in non-communicating hydrocephalus still stands. It is important to note that this does not require absorption across the blood–brain barrier as the fluid may pass through the periventricular parenchyma, which is oedematous, to other sites of absorption (see section 4.2.4 in [15]). There is even evidence that some such absorption may occur normally for sucrose [222].
2. Injection of kaolin into the cisterna magna of cats or dogs produces a long-lasting block of CSF flow from the cisterna magna to the cranial and spinal subarachnoid spaces, an acute elevation of intraventricular pressure, and a sustained ventricular swelling. CSF production within the ventricles is maintained, but over days and weeks the ventricles do not continue to swell to accommodate the added CSF. Thus chronically the CSF must escape. It was assumed by those who developed this model that the route was by ventricular absorption [220, 497, 498] and it was used in attempts to investigate this process. However, it has now been shown convincingly, at least in cats, that in chronic kaolin induced hydrocephalus there is almost no ventricular absorption [223]. Instead (see Fig. 5) CSF flows into the swollen central canal of the spinal cord [499], then across swollen or damaged spinal tissue to the spinal subarachnoid space and finally flows out via spinal nerve roots [223, 224]. Demonstration that ventricular absorption did not account for CSF outflow in this model appears to have discredited the possibility of such absorption despite the compelling evidence for it in non-communicating hydrocephalus described above.
3. When markers are injected into the cisterna magna of normal subjects or experimental animals they distribute rapidly into the cranial subarachnoid space and less rapidly into the spinal subarachnoid space but not to any observable extent into the lateral and third ventricles. By contrast in communicating hydrocephalus, i.e. hydrocephalus in which the pathways connecting the ventricles and cisterna magna are functional, the markers penetrate and accumulate in the ventricles and to some extent in periventricular parenchyma (see e.g. chapter 4 in [225] and [226, 227]). It is, as if there is a reversed flow of CSF carrying the markers through the cerebral aqueduct in hydrocephalus, but not normally. The reversed net flow implies an important source of CSF other than the choroid plexuses in the lateral and IIIrd ventricles. If the rate of secretion by the choroid plexuses is proportional to choroid plexus mass that from the choroid plexus in the IVth ventricle is not sufficient and there must be a non-choroidal source.
  PC-MRI studies in normal subjects and patients with communicating hydrocephalus have revealed that the flow of CSF through the cerebral aqueduct varies with time with a flow that is directed from the IIIrd to the IVth ventricle in systole and from IVth to IIIrd in diastole. If this accounts for the non-random variation in the CSF flow, the net, average flow over time can be calculated by taking an average over a cardiac cycle. The "noise" and variations from one recording to the next have usually been reduced by calculating an average over a number of recordings synchronized (i.e. with recording gated) to the cardiac cycle. However, this second stage of averaging will attenuate any and every component of flow *changes* that is not synchronous with the cardiac cycle, not just random fluctuations. Dreha-Kulaczewski et al. [500] have reported that the major variations in CSF flow through the aqueduct follow the respiratory rather than the cardiac cycle and it is indeed plausible as they state that these variations in CSF flow would not have been seen in the cardiac-gating studies. Whether changes in CSF flow through the aqueduct in the respiratory cycle distort the calculation of the net flow in the cardiac gating studies depends on whether or not the signal recorded in the individual traces varied linearly with flow over the entire range of flows occurring and whether enough cycles were averaged. If both criteria were met the net flow would still be correct and the inference of reversed net flow, fourth to third ventricle, from the data in these studies would still stand.

10
*Rate of fluid secretion by the blood–brain barrier. Does ISF need to mix with CSF to reach lymph or blood?* The available evidence does not exclude a fluid secretion rate across the blood–brain barrier as large as that for the choroid plexuses. However, if the secretion rate were larger than say 50% of choroid plexus secretion rate and outflow of ISF from the parenchyma were to CSF, then the estimates of the rate of CSF production rate would be markedly different if based on ventriculo-cisternal perfusion experiments on the one hand and e.g. collection of CSF from the lumbar sac on the other (see Sect. 3.2). The difference would have been larger than those seen [17]. Thus if the secretion rate is so large, much of the ISF must leave the brain without mixing with CSF that can be obtained from the lumbar sac. Lack of mixing was proposed by Cserr, Bradbury and colleagues [202, 501, 502] based on the relatively small proportion of markers injected into the parenchyma that could be recovered from CSF drawn from the cisterna magna. More recent work has emphasized routes that do not entail mixing of the outflow from the parenchyma with CSF even in the subarachnoid space [206, 459, 460, 466, 503–506] (see also section 4.1 in [15] for discussion).

11
*Hydrostatic pressure gradient needed to drive fluid movement across the blood–brain barrier* If we take a volume transfer of 200 ml day^−1^ containing 0.15 M NaCl into a 1400 g brain, the required flux, *J*_*req*_, is 21 µmol g^−1^ day^−1^ or 0.25 nmol g^−1^ s^−1^. Based on influx of radiolabelled Na^+^ from the blood, the permeability of the blood–brain barrier to Na^+^ times the surface area of the barrier per gram of brain, is *PS* = 1 to 3 × 10^−5^ cm^3^ g^−1^ s^−1^ [16, 261] and the value for Cl^−^ is similar. Using the midpoint of this range, the concentration difference required is then Δ*c* = *J*_*req*_/*PS* = 12.5 mM for each of Na^+^ and Cl^−^. As a driving force for water movement a concentration difference corresponds to an osmotic pressure difference
$$ {{\Delta }}\pi = RT\mathop \sum \nolimits {{\Delta }}c $$
with *RT* = 19 mmHg mM^−1^ and thus the concentration gradient needed to drive the flux of NaCl corresponds to an osmotic pressure difference of 500 mmHg, more than ten times larger than any possible hydrostatic pressure difference across the barrier (this point has been made repeatedly before, see e.g. [507] and for further discussion [15] section 2.7). Put the other way round active transport of solute across the barrier can easily produce solute gradients that would produce osmotic pressure differences far more important than any possible hydrostatic pressure differences.

12
*Effect of reducing [K* ^+^ *]*_*on influx of ISF*_^42^K^+^ Bradbury, Segal and Wilson [259] found that reducing [K^+^]_*ISF*_ produced a 50% increase in the amount of ^42^K^+^ from plasma that accumulated in the parenchyma over a 2-h period. They suggested two possible explanations: entry of K^+^ occurs by a mechanism that displays a long-pore effect; and sufficient ^42^K^+^ accumulates within 2 h for efflux to substantially reduce the net accumulation. Decreasing [K^+^]_ISF_ would then by decreasing the rate of efflux decrease this effect, leading to an increase in the net amount accumulated. A long-pore effect occurs when permeation is via a long, multiply-occupied, single-file pore. Well known examples include pores formed by gramicidin A [508, 509] and the delayed rectifier K^+^ channels found in nerve and muscle [510]. In essence the long-pore effect can greatly reduce the unidirectional flux of ions through the pore in the direction counter to the net flow. This explanation is difficult to sustain for ^42^K^+^ influx across the blood–brain barrier because transport of K^+^ into the endothelial cells across the luminal membrane is thought to be mediated by NKCC1 while transport out of the cells in the abluminal membrane is in the direction of the net flow through the channels (see Sect. 4.5.3). Explanation in terms of enhanced efflux of ^42^K^+^ also encounters difficulties. The half-time for accumulation of K^+^ in the brain parenchyma is of the order of 19 h [250] and thus even over a time as long as 2 h, the small increase in [^42^K^+^]_ISF_ will not lead to sufficient efflux to reduce the net rate of accumulation to an extent that changes in this efflux would matter. Perhaps, a closely related, potentially larger effect might be sufficient. To enter the brain ^42^K^+^ must cross the endothelial cell into the basement membrane on the abluminal side and then move onwards either through or around the astrocyte endfeet (see Sect. 5). [^42^K^+^] in the basement membrane may increase more rapidly and to a greater extent than in the parenchyma as a whole. (A similar effect is considered for amino acids in footnote 3.) However, even this is not a convincing explanation because the astrocyte endfeet contain high densities of K^+^ channels, which would minimize this effect. As the simple explanations have proved wanting, it would appear to be necessary to consider some form of regulation of the number or activity of K^+^ transporters.

13
*Na* ^+^ *tracer influx and efflux data at the blood–brain barrier* Davson and Welch [264] measured changes in concentration of tracers in CSF and the parenchyma when ^22^Na^+^ was infused intravenously and interpreted these using a model for exchanges between blood and CSF, CSF and brain parenchyma, and blood and brain parenchyma. The net flux across the blood–brain barrier was modelled as permeability multiplied by the concentration difference across the barrier. From their data they calculated a *PS* product (the product of the permeability and the area of barrier, usually per gram of tissue) of 0.074 cm^3^ h^−1^ g^−1^ for Na^+^ influx and a slightly higher value for Cl^−^ influx. Cserr et al. [511] measured the rate of Na^+^ efflux after injecting ^22^Na^+^ into the parenchyma of rats, and found a rate constant of 0.43 h^−1^. For a passive permeability this can be converted to a *PS* product by multiplying by the extracellular volume per unit weight of tissue, which in effect they took to be c. 0.16 cm^3^ g^−1^. Because the resulting estimate of *PS* based on tracer efflux, 0.43 h^−1^ × 0.16 cm^3^ g^−1^ = 0.069 cm^3^ h^−1^ g^−1^, is within the range of the estimates based on tracer influx, their results confirm that the net flux is too small to measure by these techniques, but the range of possible values is also too large to allow these measurements to be used as an argument against secretion of Na^+^ and fluid by the blood–brain barrier.

14
*Curve fitting of data for influx of* ^*36*^Cl^−^ *into brain cortex* Smith and Rapoport [269] measured the uptake of ^36^Cl^−^ into a volume of brain parietal cortex over a period, *T*, and divided the average uptake rate (units mmol l^−1^ s^−1^ or mM s^−1^) by the integral of [^36^Cl^−^] over the same period to obtain a transfer constant (see Eq. 1). This is then multiplied by [Cl^−^]_plasma_ to obtain the unidirectional Cl^−^ influx over the same period, with results shown in Fig. 10 (Fig. 3b in [269]). These data have been fitted here by non-linear least squares regression assuming that transport has two components:
${\text{influx}} = {{V_{max} \left[ {{\text{Cl}}^{ - } } \right]} \mathord{\left/ {\vphantom {{V_{max} \left[ {{\text{Cl}}^{ - } } \right]} {\left( {K_{m} + \left[ {{\text{Cl}}^{ - } } \right]} \right)}}} \right. \kern-0pt} {\left( {K_{m} + \left[ {{\text{Cl}}^{ - } } \right]} \right)}} + P\left[ {{\text{Cl}}^{ - } } \right]$. The short dashed curve is the fit reported by Smith and Rapoport with *P* = 0, *V*_*max*_ = 250 ×^−5^ mM s^−1^, *K*_*m*_ = 43 mM, and, as calculated here, a residual sum of squares of 5555 × 10^−10^ (mM s^−1^)^2^. The solid curve is the best fit with *P* = 0.88 × 10^−5^ s^−1^, *V*_*max*_ = 103 × 10^−5^ mM s^−1^, and *K*_*m*_ = 14 mM and a residual sum of squares of 4341 × 10^−10^ (mM s^−1^)^2^. For an F test on the improvement in fit provided by allowing *P* to vary (see [512]), p < 0.03. The long-dashed straight line is for *P* = 1.2 × 10^−5^ s^−1^, *V*_*max*_ = 56 ×^−5^ mM s^−1^, *K*_*m*_ = 0, and a residual sum of squares of 4918 × 10^−10^ (mM s^−1^)^2^. The proportions of the influx for [Cl^−^]_plasma_ = 118 mM by the unsaturable component are 0, 53 and 72% respectively in the three fits. Two conclusions follow from comparisons of these fits. Firstly the data suggest that more than half of the influx (blood to brain) occurs by an unsaturable mechanism and secondly the data do not determine an accurate value for the *K*_*m*_ of the saturable component. To reach firmer conclusions either more accurate data must be obtained or the data must extend over a larger range of [Cl^−^]_plasma_, which might be possible at the lower end of the range.

15
*Calculated net flux if the mechanism of tracer influx is electrodiffusion* For electrodiffusion of ions across a barrier the unidirectional fluxes, $\overrightarrow {J}$ and $\overleftarrow {J}$ and net flux, $J_{net}$ can be written terms of rate constants for transfers in the two directions, i.e. for Na^+^ moving between plasma (*p)* and ISF (i),
$$ \begin{aligned} \overrightarrow {J} &= k_{p} \left[ {Na^{ + } } \right]_{p} \quad \quad \quad \overleftarrow {J} = k_{i} \left[ {Na^{ + } } \right]_{i} \\ J_{net} &= \overrightarrow {J} - \overleftarrow {J} = k_{p} \left[ {Na^{ + } } \right]_{p} - k_{i} \left[ {Na^{ + } } \right]_{i} \\ \end{aligned} $$
where, because these equations must reduce to the Nernst equation at equilibrium, the rate constants must obey
$$ {{k_{i} } \mathord{\left/ {\vphantom {{k_{i} } {k_{p} }}} \right. \kern-0pt} {k_{p} }} = e^{{\frac{F\Delta V}{RT}}} $$.
*F* is the Faraday, *R* the gas constant, *T* the absolute temperature and, $\Delta V = V_{i} - V_{p}$ is the potential in ISF minus that in plasma. When the Na^+^ concentrations are the same on the two sides and the potential difference is small, this becomes
$$ \begin{aligned} J_{net} &= \left[ {Na^{ + } } \right]_{p} k_{p} \left( {1 - \frac{{k_{i} }}{{k_{p} }}} \right) = \left[ {Na^{ + } } \right]_{p} k_{p} \left( {1 - e^{{\frac{F\Delta V}{RT}}} } \right) \\& \cong - \left[ {Na^{ + } } \right]_{p} k_{p} \frac{F\Delta V}{RT} = - \overrightarrow {J} \frac{F\Delta V}{RT} \\ \end{aligned} $$

16
*Water permeabilities* It should be noted that the constants considered here describe permeation of just water. They can not be used to describe flow of the entire fluid crossing the blood–brain barrier including solutes in response to a hydrostatic gradient. The hydraulic permeability of a barrier to water, *L*_*p*_, is defined as the ratio of the volume flow of water per unit area, *J*_*V*_, to the net pressure difference, hydrostatic plus osmotic, Δ*P*_*total*_. Fenstermacher and Johnson [287] measured and reported a filtration constant defined as *J*_*V*_/Δ*c* where Δ*c* is the concentration difference of impermeant solutes (which for this purpose includes Na^+^ and Cl^−^). The filtration constant equals *L*_*p*_ *RT* where *R* is the gas constant, 8.3 J mol^−1^ K^−1^ and *T* is the absolute temperature. At 37 °C, *T* = 310 K and *RT* = 2576 J mol^−1^ = 2576 N m^−2^ mM^−1^ = 19.4 mmHg mM^−1^. For calculation of the filtration constant they assumed that the surface area of capillaries per gram of parenchyma was 52 cm^2^ g^−1^. Fenstermacher [288] recalculated the value assuming 100 cm^2^ g^−1^ with the result *LpRT* = 1.2 × 10^−6^ ml min^−1^ cm^−2^ mM^−1^. To facilitate comparison with the tracer permeability of water, *P*_*d*_ = (flux of tracer per unit area)/(difference in tracer concentration), the osmotic water permeability is sometimes defined as $P_{f} = {{L_{p} RT} \mathord{\left/ {\vphantom {{L_{p} RT} {\bar{v}_{w} }}} \right. \kern-0pt} {\bar{v}_{w} }}$ where $\bar{v}_{w}$ is the partial molar volume of water. Fenstermacher and Johnson's value becomes *P*_*f*_ = 1.1 × 10^−3^ cm s^−1^ which as stated in Sect. 4.3.6 is well within the range of values found for lipid bilayers. From the filtration constant and an estimate of the area of membrane per gram of parenchyma, and the expression relating net water flow and concentration difference of impermeant
$$ J_{V} = L_{p} RT{{\Delta}}c $$
it is possible to calculate the concentration difference of NaCl that would be needed to drive a net flow of 0.1 µl g^−1^ min^−1^ (corresponding to 200 ml day^−1^ in a human). Using 100 cm^2^ g^−1^ (the value used in [288]), and 1.2 × 10^−6^ ml min^−1^ mM^−1^ cm^−2^,
$$ \begin{aligned} \Delta \left[ {\text{NaCl}} \right] &=\Delta c/2 = J_{V}/(2L_{p}RT) \\ &= \frac{{ 0. 1 \,\upmu{\text{  l g}}^{ - 1} \,{\text{min}}^{ - 1} \times 10^{ - 3}\, {\text{ml}}\, \upmu {\text{l}}^{ - 1} }}{{2 \times 1.2 \times 10^{ - 6} \,{\text{ml min}}^{ - 1} {\text{mM}}^{ - 1} {\text{cm}}^{ - 2} \times 100{\text{ cm}}^{2} \,{\text{g}}^{ - 1} }} \\ &= 0. 4 {\text{ mM}} \end{aligned}$$

17
*Water cotransport * The movement of water by cotransport with ions or other solutes either by direct coupling or by local osmotic effects within a transporter vestibule [160] may be large enough to contribute to the net water flux across the blood–brain barrier. If so, the osmotically driven flow might be decreased or even changed in direction so that the final result is still a fluid close to osmotic equilibrium. Coupled transport of 40 water molecules with each glucose molecule has been proposed for GLUT1 in another context [157]. (This is in addition to the water permeability induced by GLUT1.) At the blood–brain barrier with a net glucose transfer of 0.6 mol day^−1^ this would mean addition of 24 mol day^−1^ (440 ml day^−1^) of water to the brain without accompanying osmotically active solutes (the glucose is consumed). This combined with metabolically produced water would mean that the osmotically driven water flow across the blood–brain barrier would be out of the brain.

18
*The pNPP-ATPase assay for localization of the Na* ^*+*^,* K* ^*+*^ *-ATPase* Ernst [302, 303] introduced a procedure for localizing the pNPP-ATPase activity of the Na^+^-pumps based on the hydrolysis of pNPP (*p*-nitrophenylphosphate) in a K^+^ dependent step, capture of the phosphate using strontium, and subsequent conversion to a stable lead precipitate which can be seen in the electron microscope, the so-called two-step or indirect procedure. Mayahara et al. [304] introduced a substantial simplification of the procedure to allow omission of the strontium step, the so-called one-step or direct procedure. In both methods the tissue is fixed prior to the enzyme assay step using formaldehyde with or without glutaraldehyde. Prior fixation entails the risk that the ATPase will be inactivated. Mayahara reported that 2% formaldehyde +0.5% glutaraldehyde was the best compromise between adequate fixation and loss of enzyme activity. The pNPP-ATPase assay is not selective for Na^+^-pumps. It also detects alkaline phosphatase, which can be eliminated from the results by inclusion of a suitable inhibitor, e.g. levamisole. However, even so it is necessary to demonstrate that the activity detected requires the presence of K^+^ and is inhibited by ouabain, usually at 1 mM. Manoonkitiwongsa et al. [311] investigated the effects of a range of concentrations of formaldehyde and glutaraldehyde. They found that fixation with 2% formaldehyde yielded 1.5 as the ratio of the luminal to abluminal product densities but with 2% formaldehyde plus glutaraldehyde at 0.1, 0.25 or 0.5% the ratio decreased to 0.7, 0.5 or 0.4 [311]. They concluded that glutaraldehyde had an effect to selectively decrease luminal activity. Arguing against this, previous studies that had found strong abluminal predominance include those with (e.g. [295, 305]) and without [308] glutaraldehyde. It may be significant that Manoonkitiwongsa et al. reported that some activity (always in the presence of levamisole to inhibit alkaline phosphatase) persisted in the absence of K^+^ or in the presence of ouabain, but even though the measured hydrolysis thus had to represent more than one type of activity, they still used the total measured activities to compare luminal and abluminal activities.

19
*Net rate of acid extrusion* At steady-state [HCO_3_^−^] and [H^+^] are constant inside the cells and there is no net accumulation of acid within the cells. Thus the net rate of acid extrusion from the cells must be balanced by the net rate of acid production within them as part of metabolism. Most of the acid production is in the form of CO_2_ that is extruded from the cells as such. The next most important source is production of lactic acid, but this is extruded as such by MCT1 (see Sect. 6.3). Other contributions can arise from metabolism of fats and protein, but these are at such low rates that they do not affect the conclusions in this section. Thus the net rate of acid extrusion, other than as CO_2_ and lactic acid, must be close to zero.

20
*Possible effect of upregulation of luminal membrane K* ^*+*^ *channels* Another possibility is suggested by the schemes shown in Figs. 17 and 18. Instead of downregulation of NKCC1 that mediates a net flux into the cells, there might be upregulation of K^+^ channels in the luminal membrane that mediate a net flux out of the cells. Tracer that enters via NKCC1 can either return to plasma or continue onwards towards ISF. Increasing the rate of return would decrease the fraction of K^+^ entering that reaches ISF.

21
*Compensation and relative changes in pH*_*arterial*_ *and pH*_*CSF*_ In metabolic acidosis decreased [HCO_3_^−^]_arterial_ and pH_arterial_ increase ventilation rate over time and decrease pCO_2_ which reduces the size of the decrease in [HCO_3_^−^]_arterial_/pCO_2,arterial_ and hence (see Sect. 6.1.1) reduces the decrease in pH_arterial_. This reduction in the size of the change in pH_arterial_ is called respiratory compensation. Respiratory compensation also reduces the size of the increase in pH_arterial_ in metabolic alkalosis by increasing pCO_2_. Tighter regulation of pH_CSF_ than of pH_arterial_ is achieved because while the changes in pCO_2_ are similar in CSF and arterial plasma, the change in [HCO_3_^−^]_CSF_ is less than that of [HCO_3_^−^]_arterial_ as shown in Fig. 21c, d. There would be perfect regulation of pH_CSF_ if the fold change in [HCO_3_^−^]_CSF_ were equal to the fold change in pCO_2,CSF_, i.e. if the new values of [HCO_3_^−^]_CSF_ and pCO_2,CSF_ were the same factor times their old values. In respiratory acidosis increased pCO_2_ and decreased pH_arterial_ increase metabolic production of HCO_3_^−^ which increases [HCO_3_^−^]_arterial_ and in turn reduces the size of the decrease in [HCO_3_^−^]_arterial_/pCO_2,arterial_ and hence the decrease in pH_arterial_. This reduction in the size of the change in pH is called metabolic compensation. Note that pH_arterial_ is still decreased because in compensated respiratory disturbances the fold change in [HCO_3_^−^]_arterial_ is still smaller than the fold change in pCO_2_. For there to be tighter regulation of pH_CSF_ than of pH_arterial_ in respiratory acidosis it would be necessary for the fold change in [HCO_3_^−^]_CSF_ to be closer to the fold change in pCO_2_, i.e. the fold change in [HCO_3_^−^]_CSF_ would have to exceed the fold change in [HCO_3_^−^]_arterial_. This is not the case in the data for human respiratory acidosis shown in Fig. 21b nor for more recent data (see [185]) where the fold changes in [HCO_3_^−^]_CSF_ are similar to or smaller than those in [HCO_3_^−^]_arterial_.

22
*H* ^*+*^-* and* *CO*_*2*_-*related species that can carry charge across membranes *H^+^ and HCO_3_^−^ are not the only related species that can carry charge across a membrane. Very rarely in studies of epithelial transport it has been found necessary to consider fluxes of OH^−^ (see e.g. [513]). However, the properties of a transporter that would allow selective, rapidly reversible binding of OH^−^ have not yet been described. Because evidence for OH^−^ transport has not been reported in studies of the choroid plexuses and blood–brain barrier, it has not been considered in this review. Another possible species that may be transported is CO_3_^2−^. For instance it is very difficult experimentally to distinguish coupled transport of one Na^+^ and one CO_3_^2−^ from coupled transport of one Na^+^ and two HCO_3_^−^. Both transfer one negative charge and both require the presence of CO_2_ [195, 398].

23
*Stewart's approach to the interdependence of ion concentrations and fluxes in acid-base balance and a better but more difficult alternative* The interdependence of ion concentrations and fluxes relevant to the control of pH was emphasized in an approach to the subject presented by Stewart [407, 408, 514]. In this approach, the independent variables that can be altered or controlled are taken to be pCO_2_, the strong ion difference (i.e. the sum of charges on ions like Na^+^ and Cl^−^) and the concentration of a single "representative" buffer standing for all buffers present other than CO_2_ / HCO_3_^−^. All other variables like pH and [HCO_3_^−^] are regarded as dependent and not subject to separate control. A major advantage of Stewart's approach is that it allows many acid-base calculations, even involving transfers across membranes, to be completed without any consideration of membrane potential. A major disadvantage of Stewart's approach is that it becomes tempting to make statements like: "Hydrogen ion movements between solutions can not affect hydrogen ion concentration; only changes in independent variables can." [514] or "Furthermore there are objections, based on physicochemical principles, to the assumption that HCO_3_^−^ (or H^+^) is primarily and directly handled by active-transport mechanisms" (pg 127 in [185]). The latter statement is just wrong. It would have been much better had Stewart said "The observation of changes in hydrogen ion concentration implies that there are also changes in pCO_2_, the strong ion difference or the concentrations or properties of the buffers." Stewart's proposals were useful in that they stimulated consideration of the consequences for acid-base balance of fluxes of those ions that determine the strong ion difference [185, 389, 393]. However, despite statements to the contrary (see e.g. pp. 120–121 in [185]), Stewart's choice of which variables to consider as independent is only a matter of convenience not one of necessity (compare [383, 390, 515]). Thus if all that is known is that there have been changes in [HCO_3_^−^] and [Cl^−^] it is not possible to say whether either change caused the other. Stewart's approach is of no help in considering the role of changes in membrane potential. Rather than defining some variables as always independent and some as always dependent as Stewart did, it is much better not to prejudge which concentrations and fluxes can be varied by external processes (e.g. transport into or out of a region) and instead use the explicit constraints on the concentrations and fluxes described in Sect. 6.1. Indeed, now that computer simulations can keep track of concentrations to the accuracy needed for calculation of the net charge within a region as big as a cell, it is possible to do even better and take a more rigorous approach avoiding the need to assume electroneutrality (a necessary part of Stewart's approach). The analysis should employ a model that considers the electrical potentials produced by the actual net charge within cells, the effects of these potentials and the ion concentrations on transport of ions, and the changes in intracellular net charge and ion concentrations produced by such ion transport. Electroneutrality is not imposed, but if the results of the analysis do not obey approximate electroneutrality (i.e. the membrane potentials fail to take on realistic values) either the model is describing a condition which would destroy the cell or something is wrong with either the model or the computer code used to implement it. Students of electrophysiology will recognize that important elements of this approach, avoiding the need to assume electroneutrality, were used in the classical description of the mechanism for propagation of the action potential along a nerve axon [516]. Changes in charge and potential could be calculated in that study without needing to know the concentrations to great accuracy because the potentials and the current across the membrane were measured directly. There are, however, challenging aspects to extending this more rigorous approach to the choroid plexuses and blood–brain barrier: it requires detailed description of the actual transport processes occurring including their dependence on membrane potential and it requires sufficiently accurate bookkeeping of ion fluxes to calculate the changes in net charge within the cell.

24
*Physiological buffering of pH*_*ISF*_ Ahmad et al. [415] used surface pH and Cl^−^ electrodes applied to exposed cortex and reported very rapid, equal but opposite changes in ISF [Cl^−^] and [HCO_3_^−^] following elevation of pCO_2_. For a pCO_2_ increase from ~28 to 55 mmHg, [Cl^−^] decreased and [HCO_3_^−^] increased by 4 mM with half-times of 30–40 s, much as expected for buffering within the cells and exchange of intracellular HCO_3_^−^ for extracellular Cl^−^. Similarly fast changes in ISF pH following changes in pCO_2_ have been seen using microelectrodes inserted into the parenchyma [456, 517] and similarly fast surface pH electrode responses have been correlated with phrenic nerve activity indicating activation of medullary chemoreceptors [517, 518]. See footnote 30 for caveats concerning results obtained in a related study by Ahmad et al. [442] that may complicate interpretation of the results discussed here. Physiological buffering of ISF is likely to be a major part of the explanation of results for ISF pH obtained using ^31^P NMR by Portman et al. [416]. Goats were ventilated with 0.5–1% halothane in oxygen at rates between 2 and 21 l min^−1^, which produced values of arterial pH between 7.1 and 7.65. ISF pH and intracellular pH were recorded 10 min after each change in ventilation rate. As percentages of the changes in arterial blood, the changes in ISF and intracellular fluid pH were 56 and 23%. These and other results obtained using ^31^P NMR [519, 520] imply that the intracellular fluid is strongly buffered against changes in pH as expected since this fluid is rich in proteins and phosphate compounds. Because the change in ISF pH was intermediate between those in plasma and intracellular fluid, both of which are regarded as strongly buffered solutions, the results also imply that ISF was strongly buffered even though ISF does not obviously contain sufficient buffers. Physiological buffering provides a plausible explanation. When pCO_2_ is increased, intracellular [HCO_3_^−^] is increased and thus [HCO_3_^−^]_ISF_ can be increased by efflux of HCO_3_^−^ from cells. There could also be transport of HCO_3_^−^ into ISF from plasma. The increase in [HCO_3_^−^]_ISF_ then reduces the change in pH to the level observed.

25
* Arterio-venous differences in concentrations for substrates and metabolites* The amount of substance taken up by or removed from a region is often estimated from the differences in the concentrations in the arterial inflow and venous outflow from the region and the blood–flow,
$$ {\text{uptake or release }} = \, \left( {C_{\text{arterial}} - C_{\text{venous}} } \right) \, \times {\text{ blood flow}}. $$
For substrates for which most movement is uptake, e.g. glucose and O_2_, the uptake is reasonably estimated by this calculation. It is, of course, necessary to arrange to take the venous sample from a location exposed to the venous outflow from the region and to none other. However, for metabolites the assumption that removal from the region occurs only by release into the venous blood emerging from that region is incorrect if some can be removed to lymph. This may be important when trying to estimate the rate of production of lactic acid as it is clear that some of the lactate is removed via perivascular routes to lymph which bypasses the sites at which the venous blood is sampled [57, 434, 521].

26
*Blood–brain barrier permeability for lactate and rapid distribution of lactate via astrocytes* The permeability-surface-area products, PS, for lactate transport by MCT1, about 0.06 ml g^−1^ min^−1^ in adult rats [429, 430] and 0.1 ml g^−1^ min^−1^ in humans [431], and the volume of ISF per gram of tissue, ~0.2 ml g^−1^, corresponds to a half-time for removal of lactate less than 10 min. (For a different view of the rate of removal see [522]). Some removal of lactate^−^ as such will occur as part of the outflow of ISF from the interstitial spaces of the parenchyma. However, as that has a half-time more than tenfold longer (judged by e.g. removal of markers like sucrose and mannitol, see Sect. 4.1 here and section 4.1.1 in [15]), such outflow will only make a relatively small contribution. However there may be another possible route of elimination of lactate^−^. Lactate^−^ or lactic acid can be removed from its site of production and distributed through the network of astrocytes (see e.g. [106, 521, 523, 524]) with release at sites close to perivascular spaces of larger blood vessels. From these spaces it may be able to leave the brain rapidly to blood or cervical lymph [57, 521]. The factors shown by Lundgaard et al. [434] to affect similarly the rates of removal of lactate and inulin may be acting at this final stage—i.e. on transport via the perivascular spaces. Distribution or spreading out of lactate from its site of production was proposed earlier [433] together with another complicating factor in the experimental studies, partial metabolism of lactate with storage of the radiolabel in metabolic intermediates [56]. In the steady-state the net rate at which lactic acid production and lactate^−^ removal provides H^+^ that can deplete HCO_3_^−^ is difficult to determine from the available evidence. It is likely to be substantially less than the rate of production of lactic acid until this rate approaches the transport capacity of MCT1 in the endothelial cells but, for severe hypocapnia or hypoxia with high [lactate^−^], depletion of HCO_3_^−^ and reduction of pH_ISF_ are likely to be substantial.

27
*Potential differences across membranes and across cell layers* Movements of ions across the membranes of either choroid plexus epithelial cells or blood–brain barrier endothelial cells via mechanisms that transport net charge must: (a) be sensitive to the electrical potential differences between the cell interiors and the blood on one side and CSF or ISF on the other and (b) affect the charge within the cells and hence the potentials across their membranes. There is thus no avoiding the need to consider the membrane potentials in any adequate discussion of mechanisms. Unfortunately, there are no available data for how the membrane potentials of the cells in vivo are affected by pCO_2_, pH or HCO_3_^−^ or how the membrane potentials affect the rates of ion transport into or out of the cells. However, the potential difference between CSF (and to some extent ISF) and blood (called "the PD") has been measured in a large number of studies. There are two issues to consider: whether and how pH, [HCO_3_^−^] or pCO_2_ in some way determine the PD and whether and how the PD affects the relation between the plasma and CSF values of pH and [HCO_3_^−^]. Data for ISF would be more interesting, but most relate to CSF. In dogs and goats the PD between cisternal CSF and jugular vein blood at pH 7.4 is between 3 and 6 mV (CSF positive) at pH 7.4. The PD changes with a slope of −32 mV (pH unit)^−1^ when pH is varied by making primary changes in pCO_2_ of blood and −43 mV (pH unit)^−1^ when the primary changes are in [HCO_3_^−^]_arterial_ [286]. See [525] (discussion after [401]) [438, 439] and for many further references [185, 388]. From the similar effects of increasing pCO_2_ and decreasing [HCO_3_^−^]_arterial_, it is inferred that the variation in PD depends primarily on [H^+^]_arterial_ rather than on [HCO_3_^−^]_arterial_ or pCO_2_. (A possible explanation for the difference in slopes is that changes in pCO_2_ alter blood flow while those in [HCO_3_^−^] do not, see [189, 436] and the discussion after [401]). Three possible mechanisms by which pH_arterial_ could affect the PD have been considered (see [185, 388]). Firstly the blood facing membrane of the barrier that generates the trans-barrier PD may have a higher conductance to H^+^ than to anything else. The lack of variation with pH_CSF_ is then explained if the CSF facing membrane has a potential difference across it that does not vary with pH on either side. Secondly pH_arterial_ may affect the permeability of the blood facing membrane or the paracellular transport route to other ions. Finally pH_arterial_ might somehow alter the rate of an active current-carrying mechanism. The first explanation would require a very high permeability to H^+^ to compensate for its very low concentration. It is difficult to imagine that such a large permeability would have escaped notice in studies such as those discussed in Sects. 3 and 4. The second and third explanations are more easily accepted but lack direct experimental evidence. There have also been three contenders for the location of the barrier across which the PD is generated: the ependyma and pia, which separate CSF and ISF, the choroid plexuses and the blood–brain barrier. The ependyma and pia are very unlikely sources because the barriers are leaky and non-selective between ions. The choroid plexus might generate a potential difference, but the available evidence indicates that it isn't the main source for three reasons. Firstly, direct measurements of the potential difference across isolated choroid plexus have shown either no PD [526, 527] or a PD that does not show the variation with pH_arterial_ that is seen in measurements of the PD between cisternal CSF and plasma [440, 492]. (In the dogfish the PD for isolated choroid plexus even has the opposite sign to the in vivo PD [528].) Secondly CSF production, measured by ventriculo-cisternal perfusion, is hardly changed when the PD is changed by altering pH_arterial_. In other words active secretion by the choroid plexus and the generation of the PD are not coupled [286]. Thirdly it would be difficult for a current source localized to the choroid plexuses within the ventricles to produce a potential elsewhere [529]. Furthermore if it is correct that the transport processes in the basolateral membranes of the epithelial cells are all electrically neutral [4] (see Sect. 4.3.2), transcellular transport at the choroid plexus cannot carry a current and whatever PD is produced by the choroid plexus can only be a diffusion potential across the paracellular pathway. There are two principal arguments in favour of the blood–brain barrier as the source of the PD: elimination of the other possibilities and the observation that pial microvessels, which are thought to have similar properties to those in parenchyma, do generate a potential (lumen negative) [285]. The available evidence indicates that transport at both the luminal and abluminal membranes of the endothelial cells does transfer net charge (see Sect. 4). The consequences of a PD that varies with pH_arterial_ could be considerable for pH regulation. As indicated in Sect. 8, under normal conditions [HCO_3_^−^]_CSF_ and thus presumably [HCO_3_^−^]_ISF_ are less than would be at equilibrium with [HCO_3_^−^]_plasma_ and the PD. In the so-called "passive" theory for ISF pH regulation advanced by Siesjö and colleagues [417], the active process (which might be lactic acid production) that accounts for this disequilibrium under normal conditions is assumed to be constant irrespective of pH, pCO_2_ and [HCO_3_^−^] in plasma or ISF with all changes in the ratio [HCO_3_^−^]_ISF_/ [HCO_3_^−^]_plasma_ resulting directly from changes in passive transport. If the PD were effective in driving a flux of HCO_3_^−^ or H^+^ across the blood–brain barrier, more positive PD would increase the ratio [HCO_3_^−^]_ISF_/ [HCO_3_^−^]_plasma_ and more negative PD would decrease it. This would provide a simple mechanism for pH regulation in ISF (and hence CSF). If pH_arterial_ were reduced by increasing pCO_2_, there would be an increase in [HCO_3_^−^]_plasma_ but a larger increase in [HCO_3_^−^] _ISF_ because the PD would be increased. Thus the pH change would be smaller in ISF than in plasma. Similarly if pH_arterial_ were reduced by decreasing [HCO_3_^−^]_plasma_ the decrease in [HCO_3_^−^]_ISF_ and pH_ISF_ would be smaller. Smaller pH changes in ISF than in plasma is the definition of pH regulation of ISF. Probably because this explanation of pH regulation could be tested by experiments (see Sect. 6.4), it attracted a great deal of effort and attention. In many, but not all, studies the variation of [HCO_3_^−^]_CSF_/ [HCO_3_^−^]_plasma_ was as expected from the variations in PD (see[185, 388, 417]).

28
*[HCO*_*3*_^−^ *] in the primary secretion of the choroid plexus* Variations in [HCO_3_^*−*^] in the primary secretion of the choroid plexus during hyperventilation or with raised pCO_2_ in inspired air were investigated by Ames et al. [141] in cats. Taking samples from droplets of fluid secreted into a layer of Pantopaque oil covering a lateral ventricle choroid plexus, they found during hyperventilation substantial decreases in the rates of secretion of both fluid and HCO_3_^−^ with no change in [HCO_3_^−^] (determined from charge balance, see Sect. 6.1.2). With raised pCO_2_ there was a substantial increase in both the rate of fluid secretion and [HCO_3_^−^]. These experiments established that the choroid plexuses do secrete and that the effects of changing pCO_2_ on choroid plexus secretion are at least in the correct direction to contribute to pH regulation in the brain. (But note these conditions correspond to respiratory alkalosis and acidosis for which regulation is poor, see Fig. 21.) However it has been suggested that the pantopaque oil used in these experiments may have altered the function of the choroid plexuses because it can be toxic when injected into the ventricles ([217], but see [530] for details). Exposure of the choroid plexus to this oil was avoided in other experiments but those entailed greater dissection of the brain to enclose a choroid plexus in a chamber that could capture the secreted fluid. Measuring secretion by the change in contents of the chamber Husted and Reed [440] found: (i) little change in the addition of HCO_3_^−^ to the chamber when [HCO_3_^−^] in arterial blood was reduced; (ii) little change in pH of the net fluid added to the chamber during hypocapnia and hypercapnia, implying that [HCO_3_^−^] in the secretion varied in proportion to the change in pCO_2_; and (iii) a marked effect of [HCO_3_^−^] in the chamber on the net addition of HCO_3_^−^. Increased [HCO_3_^−^] in the chamber almost eliminated addition of HCO_3_^−^, while decreased [HCO_3_^−^] increased the amount added. The variations with [HCO_3_^−^] in the chamber suggest that there is a substantial backflux of HCO_3_^−^ from chamber to blood that increases with the concentration in the chamber. It is possible that this represents an artefact produced during the dissection. All of the changes observed by Husted and Reed are in the correct direction to contribute towards pH regulation. However, in contrast to the earlier results of Ames et al. [141], Husted and Reed [440] found no effect of changes in pCO_2_ on the rate of CSF production. This discrepancy remains to be resolved. Increase in [HCO_3_^−^] in the secretion when pCO_2_ is increased is consistent with what is now known about the transporters present at the choroid plexuses. Within the epithelial cells, increased pCO_2_ will increase [HCO_3_^−^] or decrease pH to maintain the equilibrium between CO_2_, H^+^ and HCO_3_^−^ (carbonic anhydrase is present). Indeed if as favoured by current evidence the route of HCO_3_^−^ entry across the basolateral membrane is NCBE (see Sects. 4.3.2, 4.3.3), the equilibrium is likely to be maintained primarily by an increase in [HCO_3_^−^] because any decrease in pH would increase the driving force for H^+^ and Cl^−^ exit and HCO_3_^−^ entry. Increased [HCO_3_^−^] within the cells will be accompanied by a decrease in [Cl^−^] (see Eq. 11). These concentration changes will be reflected in HCO_3_^−^ being a larger proportion of the anions crossing the apical membrane, i.e. to an increase in [HCO_3_^−^] in the secretion. However, this explanation is called into question by the failure of similar reasoning to explain why an increase in plasma [HCO_3_^−^] and decrease in plasma [Cl^−^] at constant pCO_2_ does not increase [HCO_3_^−^] in the secretion.

29
*HCO*_*3*_^*−*^ *and* *Cl* ^*−*^ *permeabilities estimated from loss from ventricular perfusates* Pappenheimer, Fencl and colleagues [351, 352] (see section 5.3.1) used ventriculocisternal perfusion to look at the loss from or gain into the perfusion fluids of HCO_3_^−^ and Cl^−^ measured after 45 min of perfusion. However, it is now clear that 45 min was not long enough for concentrations in the parenchyma to reach steady-state. This is evident from experiments following radiolabelled K^+^ [251] or Na^+^ [441]. The results for Na^+^ were particularly detailed. For periods much longer than 45 min the loss of tracer from the perfusate across the ependyma corresponded to increases in the concentration within the parenchyma rather than transport to the blood. Even after 4 h of perfusion the rate of loss from the ventricles had not decreased to the steady-state value. As blood–brain barrier permeabilities to Na^+^ and Cl^−^ are now thought to be similar (see Sect. 4.3.2) and both ions are present at relatively high concentrations primarily in the extracellular fluid within the parenchyma, it follows that for Cl^−^, just as for Na^+^, perfusions lasting for hours rather than the 45 min employed would be required to reveal the rate of transfer across the blood–brain barrier. For HCO_3_^−^, an adequate theoretical treatment of the data is more difficult because the concentrations of HCO_3_^−^, CO_2_ and H^+^ are interrelated and the local pH is buffered by the presence of the cells. The measured rate of loss of Cl^−^ from the perfusion fluid was ~6 µmol min^−1^ when the concentration in CSF exceeded the value for zero loss by $c_{CSF}$ = 40 mM. It is instructive to compare this measured value with estimates of the rate of loss, R, calculated assuming that it represents:

- steady-state transfer across the blood–brain barrier, approximately:

$$ R \approx PS \times \rho \times A \times \delta \times c_{CSF} $$
or

b. diffusion into parenchyma with no transfer across the blood–brain barrier:

$$ \begin{aligned} R &= - DA\left( {{{{\text{d}}c} \mathord{\left/ {\vphantom {{{\text{d}}c} {{\text{d}}x}}} \right. \kern-0pt} {{\text{d}}x}}} \right)_{x = 0} = - DA\left( {\frac{{{\text{d}}\left( {c_{CSF} {\text{erfc}}\left( {2\sqrt {Dt} } \right)} \right)}}{{{\text{d}}x}}} \right)_{x = 0} \\& = Ac_{CSF} \sqrt {{{2D} \mathord{\left/ {\vphantom {{2D} {\left( {\pi t} \right)}}} \right. \kern-0pt} {\left( {\pi t} \right)}}} \\ \end{aligned} $$
In these expressions: *PS* is the permeability-area product for Cl^−^ at the blood–brain barrier, which has been determined by Smith and Rapoport as 1.4 × 10^−5^ cm^3^ s^−1^ g^−1^ [261]* ρ* = 1 g cm^−3^ is the density of the region; *A* is the surface area of the perfused portion of the ventricles, taken here (a guess) as 35 cm^2^; *δ* is an equivalent thickness of the layer of parenchyma from which transfer to the blood occurs, which after 45 min will be less than 2 mm [441]; $c_{CSF}$ = 40 mM is the concentration excess in the perfusate; erfc(*y*) is the complement error function [441]; *D* ~ 5 × 10^−6^ cm^2^  s^−1^ is the diffusion constant, and *t* = 45 min is the time after the start of the infusion at which the rate of loss is determined. Inserting values into the expression for loss by steady-state transfer across the blood–brain barrier, 
$$ \begin{aligned} R &\approx 1.4 \times 10^{ - 5} {\text{cm}}^{3} {\text{s}}^{ - 1} {\text{g}}^{ - 1} \times 35\,{\text{cm}}^{2} \times 0.2\,{\text{cm}} \times 1\,{\text{g}}\,{\text{cm}}^{ - 3} \times 40\,\upmu {\text{mol}}\,{\text{cm}}^{ - 3} \\ &\approx 4 \times 10^{ - 3} \,\upmu {\text{mol}}\,{\text{s}}^{ - 1} = 0.24\,\upmu {\text{mol}}\,{ \hbox{min} }^{ - 1} \\ \end{aligned} $$
This is much smaller than the measured value. Inserting values into the expression for loss from the ventricles by diffusion into the parenchyma
$$ \begin{aligned} R &\approx 35\,{\text{cm}}^{2} \times 40\,\upmu {\text{mol}}\,{\text{cm}}^{ - 3} \times \sqrt {{{2 \times 5 \times 10^{ - 6} {\text{cm}}^{2} {\text{s}}^{ - 1} } /{\left( {3.14 \times 2700\,{\text{s}}} \right)}}} \\ &\approx 0.05\,\upmu {\text{mol}}\,{\text{s}}^{ - 1} = 2.9\,\upmu {\text{mol}}\,{ \hbox{min} }^{ - 1} \\ \end{aligned} $$
This is much closer to the measured value. From this comparison it would appear that the measured rate of loss from the ventricular perfusate after 45 min was more than tenfold greater than the rate of transfer across the blood–brain barrier and could be accounted for by accumulation (or depletion) of Cl^−^ in the parenchyma. These numbers shouldn't be taken too seriously. What was needed in the studies by Pappenheimer, Fencl and colleagues was experimental evidence that the rate of loss or gain had reached a steady-state. No such evidence was presented.

30
*Rates of* *HCO*_*3*_^*−*^ *and Cl* ^*−*^ *transport inferred from pH electrode measurements* Ahmad and Loeschcke [442] using surface electrodes investigated changes when plasma [HCO_3_^−^] was abruptly increased from ~16 to ~23.5 mM at nearly constant pCO_2_. ISF [HCO_3_^−^] increased and [Cl^−^] decreased by about 3 mM (pH increase of 0.07) with a half-time of about 20 s. This was interpreted as rapid exchange of HCO_3_^−^ and Cl^−^ between plasma and ISF. Teppema et al. [443, 444] also saw rapid changes in pH. Davies and Nolan [445] using pH sensitive microelectrodes with pH sensitive tips about 1–2 µm diameter and 40 µm in length found that ISF pH responded to isocapnic, iv infusions of HCl or NaHCO_3_ with lags of only a few minutes (ΔpH_ISF_ was 40–80% of ΔpH_arterial_ at the end of 30 min with no change in pH_CSF_). These changes are much faster and larger than those seen at constant pCO_2_ by Javaheri and colleagues [455, 457]. No satisfactory reconciliation of these results has been provided [389]. The rapid changes might be explained if somehow the electrodes were responding, at least partially, to changes in the pH of plasma or peripheral tissue extracellular fluid. However, how this could have occurred is unknown. Presumably worry about such an artefact explains why Javaheri et al. always looked for a positive Rapoport test (see Sect. 6.4.2). The rapid changes in [Cl^−^] reported by Ahmad and Loeschcke [442] are not consistent with the reported tracer permeability to this ion. More generally the permeability of the blood–brain barrier to all small ions (see e.g. [16, 261, 269], and thus by inference to HCO_3_^−^, is difficult to reconcile with the reports of large, rapid changes in ISF pH at constant pCO_2_. Furthermore it is very difficult to understand how under these circumstances, given free movement of small ions across the ependyma, rapid and large changes in ISF could occur without some change with a similar time course in [HCO_3_^−^]_CSF_. All agree that no such variation is observed (see e.g. [185, 386, 413, 414].

31
*Rate of HCO*_*3*_^*−*^ *transport (or lack of) estimated from measurement of total CO*_*2*_ *in the brain* Siesjö asserted that the methods used would have quantified a change corresponding to the same concentration change as in plasma in a volume of 5% of the water content of brain and concluded that only a little HCO_3_^−^, if any, crossed the blood–brain barrier during the experiments. It is now known that the volume of ISF is about 20% of the aqueous volume of the parenchyma, which means a change in [HCO_3_^−^] in ISF of about 25% of that in plasma might not have been seen. The change expected is about 30% of that in plasma if the change in [HCO_3_^−^]_ISF_ is the same as the steady-state change in [HCO_3_^−^]_CSF_ [185].
